# Digital health and patient adherence: A qualitative study in older adults

**DOI:** 10.1177/20552076231223805

**Published:** 2024-01-12

**Authors:** Filipa Ferreira-Brito, Sérgio Alves, Tiago Guerreiro, Osvaldo Santos, Cátia Caneiras, Luís Carriço, Ana Verdelho

**Affiliations:** 1 Instituto de Saúde Ambiental (ISAMB), 37811 Faculdade de Medicina, Universidade de Lisboa, Lisboa, Portugal; 2 LASIGE, 111161 Faculdade de Ciências, Universidade de Lisboa, Lisboa, Portugal; 3 Unbreakable Idea Research, Painho, Portugal; 4 Laboratório de Investigação em Microbiologia na Saúde Ambiental (EnviHealthMicro Lab), Instituto de Saúde Ambiental (ISAMB), Faculdade de Medicina, Universidade de Lisboa, Lisboa, Portugal; 5 Healthcare Department, Nippon Gases Portugal, Lisboa, Portugal; 6 Instituto de Medicina Molecular, Faculdade de Medicina, Universidade de Lisboa, Lisboa, Portugal; 7 Neurology Service, Department of Neurosciences and Mental Health, Hospital de Santa Maria, Centro Hospitalar Universitário Lisboa Norte, Lisboa, Portugal

**Keywords:** Digital divide, digital health, computer confidence, computer self-efficacy, technology adoption

## Abstract

**Introduction:**

Computer confidence and computer self-efficacy can impact an individual's perceived ease of use and usefulness of technology, ultimately determining adherence to digital healthcare services. However, few studies focus on assessing the impact of non-clinical factors on the efficacy and adherence to digital healthcare platforms.

**Objective:**

We aimed to analyse the role of non-clinical factors (i.e. computer confidence and computer self-efficacy) in the interaction experience (IX) and the feasibility of a digital neuropsychological platform called NeuroVRehab.PT in a group of older adults with varying levels of computer confidence.

**Methods:**

Eight older adults (70.63 ± 6.1 years) evaluated the platform, and data was collected using the Think-Aloud method and a semi-structured interview. Sessions were audio-recorded and analysed through an inductive-deductive informed Thematic Analysis protocol. This study was conducted according to the Consolidated Criteria for Reporting Qualitative Research guidelines.

**Results:**

Three main themes were identified (Interaction Experience, Digital Literacy, and Attitudes toward NeuroVRehab.PT). Computer anxiety and fear of making errors were not uncommon, even among older adults who perceive themselves as confident in technology use, and negatively impacted IX. Moreover, some game elements (e.g. three-star system, progression bar) were not intuitive to all participants, leading to misleading interpretations. On the other hand, human support and the platform's realism seemed to impact participants’ IX positively.

**Conclusions:**

This study shed light on the barriers raised by non-clinical factors in adopting and using digital healthcare services by older adults. Furthermore, a critical analysis of the platform's features that promote user adoption is done, and suggestions for overcoming limitations are presented.

## Introduction

The global population is ageing, making it imperative to prioritise public health initiatives that promote cognitive functioning and functional capacity.^1^ Digital health refers to the use of digital, mobile, and wireless technologies to achieve health-related outcomes, and it has become a mainstream way to deliver healthcare services to a broad sector of the population, including older adults.^2,3^

To ensure the development of effective digital healthcare interventions, evaluating their applicability and feasibility is essential, taking into account the specific needs and characteristics of the clinical populations.^4–6^

From a technological perspective, the applicability and adequacy of digital healthcare interventions are usually determined based on participants’ interaction experience.^7^ The concept of interaction experience (IX) includes usability aspects (i.e. the extent to which a system can be used to achieve specific goals with effectiveness and efficiency); user experience (i.e. hedonic experience and user's perceptions and responses that result from the use and/or anticipated use of a system); and accessibility (i.e. the extent to which a system addresses the needs, characteristics, and abilities of a large spectrum of users with or without impairments).^8^ Although these three components are considered the ‘trinity’ of the technological strand of feasibility studies,^7^ issues related to these components when developing digital cognitive platforms are rarely mentioned or just superficially addressed.^9^

Additionally, the impact of non-clinical variables is also overlooked in the development and evaluation process of digital cognitive platforms, with the primary focus being on addressing the barriers and requirements resulting from users’ cognitive impairments with disregard for other crucial user characteristics.^1,9^

Scientific literature has shown that low digital/computer literacy, negative emotions associated with previous unsuccessful experiences with technology, computer anxiety, and low perceived computer self-efficacy can impact the adoption and use of digital healthcare interventions in older adults.^10–12^ For example, according to the eHealth Literacy model, using and adapting to computers and new technologies to solve problems (i.e. digital/computer literacy) is crucial to engage and use digital healthcare services.^13^ Moreover, negative emotions associated with previous unsuccessful experiences also determine users’ perceived ease of use and technology usefulness. For instance, Portz et al. showed that past negative experiences with technology resulted in computer anxiety (i.e. apprehension or even fear of using technology) and low perceived computer self-efficacy (i.e. confidence in the ability to ‘figure things out’ while using technology), which, in turn, negatively impacted the search for and adoption of digital healthcare services by older adults.^14^ These findings align with psychological literature, which shows that an individual's perception of their competency on a specific matter directly influences their resilience when encountering challenges.^15^

The lack of analysis of the impact of non-clinical variables during feasibility testing of digital cognitive platforms can hinder users’ ability and willingness to engage with these services and use them effectively. Therefore, it is essential to adopt a more holistic approach that considers a broader range of user characteristics to create inclusive interventions tailored to individual needs, including the necessities raised by the lack of low familiarity/proficiency in technology use that some users might present.

Grounded on this rationale, in the present study, we aimed to analyse the potential barriers created by non-clinical factors to adopting and using a digital neuropsychological platform called NeuroVRehab.PT. This platform was designed to promote cognitive functioning and functional capacity to perform an instrumental activity of daily living, namely grocery shopping.^16^ Moreover, by gaining insight into the nature of these barriers and their potential impact on IX,^7^ we aimed to contribute to developing more inclusive and effective digital neuropsychological interventions.

## Materials and methods

In this study, we conducted a phenomenological qualitative study^17^ with the objective of gaining a deeper understanding of the experiences and personal perspectives concerning the strengths and challenges associated with interacting with our platform in a group of older adults with varying levels of computer confidence and computer self-efficacy. Moreover, we adhered to the Consolidated Criteria for Reporting Qualitative Research guidelines^18^ to ensure the quality of the study's execution and reporting (see Table S1 in Supplementary Material).

### Recruitment and participants

To recruit participants, we visited two non-profit institutions that offer educational and recreational activities specifically designed for older adults (i.e. senior universities). We presented the details of our project to attendees of two Information and Communication Technologies (ICT) classes. Eight ICT attendees (Table 1) expressed willingness to participate and were screened against the eligibility criteria shown in Table 2.

**Table 1.** Demographic information and self-reported confidence using technology (n = 8).

ID	GN	Age	Education	Marital status	Confidence using technology	Confidence using new devices	Time browsing the internet/week	Play video games/other apps
P1	M	68	High school	Married	Confident	Confident	14 h	No
P2	F	78	High school	Widow	Little confident	Little confident	11 h	Sudoku game and Solitaire Spider Microsoft^®^
P3	F	77	High school	Married	Little confident	Little confident	4 h	No
P4	F	67	Primary education	Married	Confident	Confident	5 h	No
P5	F	68	University	Single	Confident	Confident	5 h	Facebook^®^ math exercises
P6	F	61	High school	Divorced	Little confident	Little confident	8 h	No
P7	M	69	High school	Single	Very confident	Very confident	10 h	No
P8	M	77	University	Widower	Confident	Confident	10 h	No
		70.6 ± 6.1 mean age					8.38 ± 3.50 mean time	

GN: gender.

**Table 2.** Study's eligibility criteria.

Eligibility criteria
(a) 60 years or older
(b) Be fluent in Portuguese (spoken and written)
(c) Live autonomously in the community
(d) Have basic skills or willingness to learn to use a tablet.

### Procedure

Participants who met the eligibility criteria were assigned to an individual session to use and evaluate NeuroVRehab.PT. Sessions took place in a private room at the senior universities’ facilities and were conducted by a neuropsychologist (FFB, PhD student) with previous experience in qualitative research. The second author (SA), a computer scientist and PhD student, was also present at the first two interviews to ensure the sessions went smoothly without technical issues.

A Huawei MediaPad T5 tablet (Android 8) (Huawei Technologies Co. Ltd, Shenzhen, China) was placed horizontally at a 25° using a tablet stand on a table in front of participants.

Before data collection, the objectives, study procedures, and participants’ rights were reviewed once again. Additionally, all participants received an informative sheet detailing the study's objectives, contact information, and the written informed consent form. Participants were encouraged to carefully read both documents and ask any questions they may have had. Demographic data, including age, sex, education level, marital status, digital literacy (self-reported confidence using technology and new technological devices, weekly time browsing the internet), and mhealth use (use of cognitive training mobile apps) were collected. All participants provided written informed consent before the start of the study.

### Instruments

The study employed individual audio-recorded interviews lasting approximately 60 min and comprosing two parts. During the first part, the Think-Aloud method was used to collect participants’ thoughts and feelings regarding their IX^7,8^ with the platform. This method was selected for its capacity to bring to the surface complicated thinking processes and problem-solving strategies that participants experience while performing a task.^19^ These thoughts are verbalised and collected as data, which researchers can analyse.^19^ In parallel with the Think-Aloud exercise, researchers documented participants’ behaviour while interacting with the platform, using field notes from direct observation. Before participants started exploring the platform, the first author (FFB) exemplified how to perform a Think-Aloud exercise using the Gmail website as an example.

The second part of the session consisted of a semi-structured interview conducted to ensure that relevant aspects of platform IX that might be neglected or omitted during the Think-Aloud exercise were collected. An interview script was collaboratively developed and discussed with the research team (see Doc S1 in Supplementary Material). This script was used to guide the interviews and encompasses topics and themes that the research team deemed pertinent for exploring with the participants. The script comprised open-ended questions on the platform's usability, inputs and feedback quality, user experience, intention to use, and the most and least appreciated/enjoyable platform features. Examples of the questions included: ‘What difficulties did you experience while using the platform?’, ‘How would you describe your experience with the platform?’, ‘How precise did you find the instructions provided by the platform?’, ‘If available to the public, will you consider using the platform on a daily/weekly basis? Why?’ and ‘What platform features did you enjoy/value most?’.

### Digital neuropsychological rehabilitation platform

**Figure 1.**
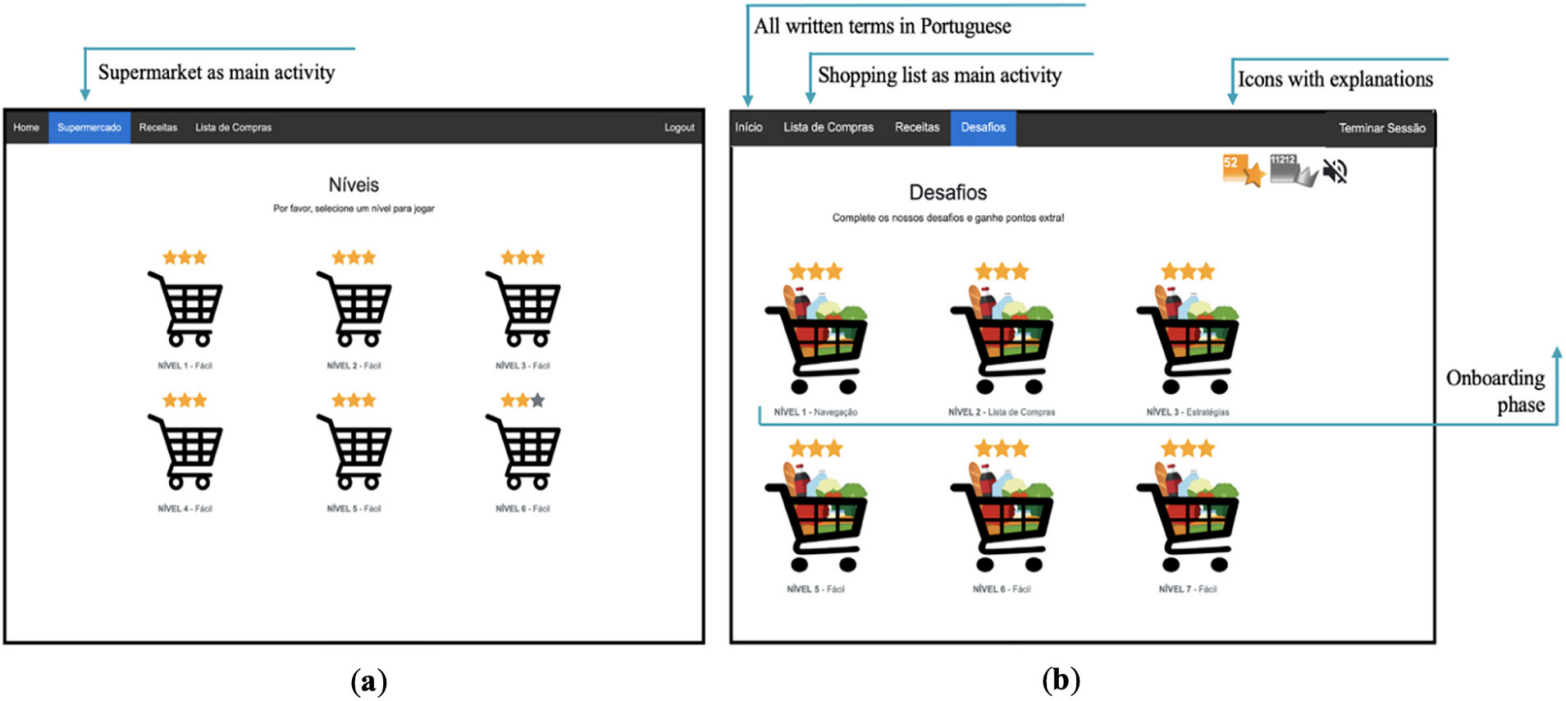
(a) Former supermarket game mode interface. The default game mode was the virtual supermarket with different game levels; (b) challenges interface with the new features. In the latest version (after this qualitative study) default game mode is the shopping list game.

**Figure 2.**
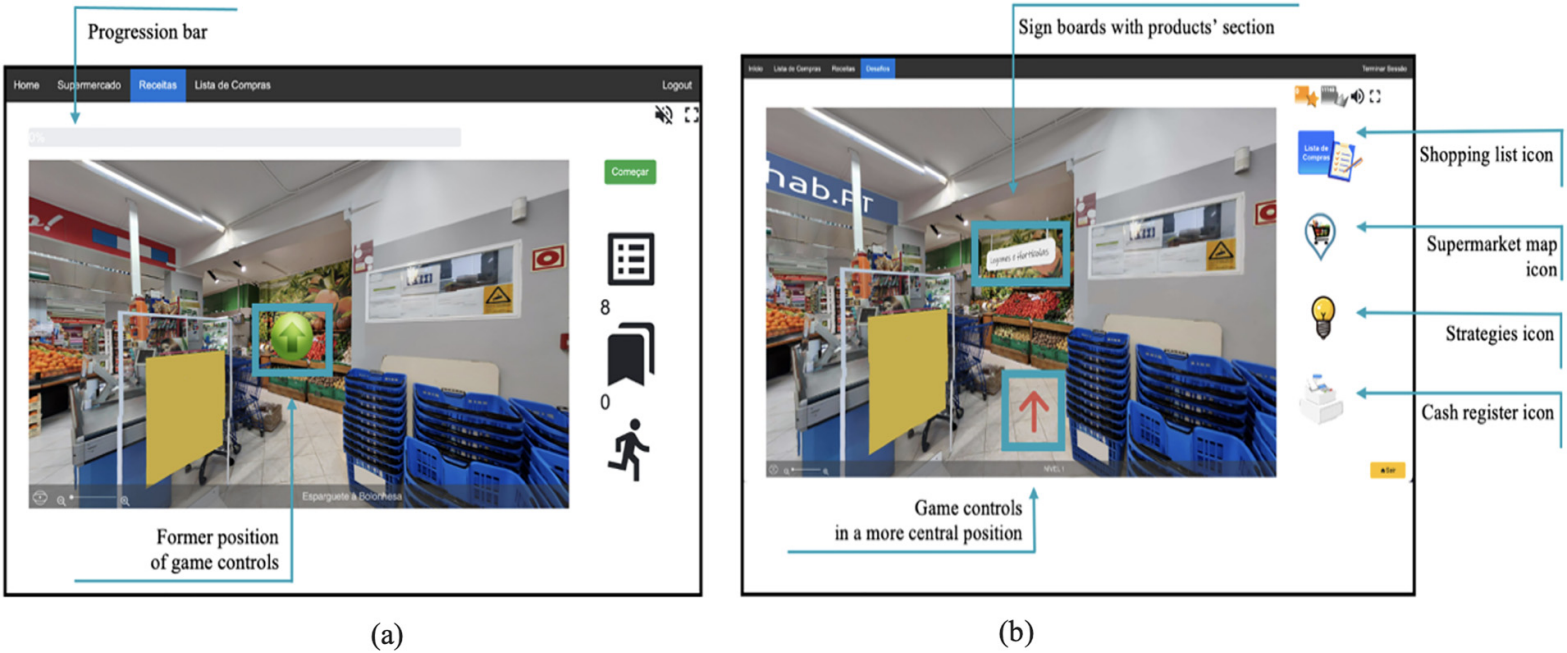
(a) Former supermarket virtual environment and interface; (b) supermarket virtual environment and interface with the (new) features identified through this qualitative study.

**Figure 3.**
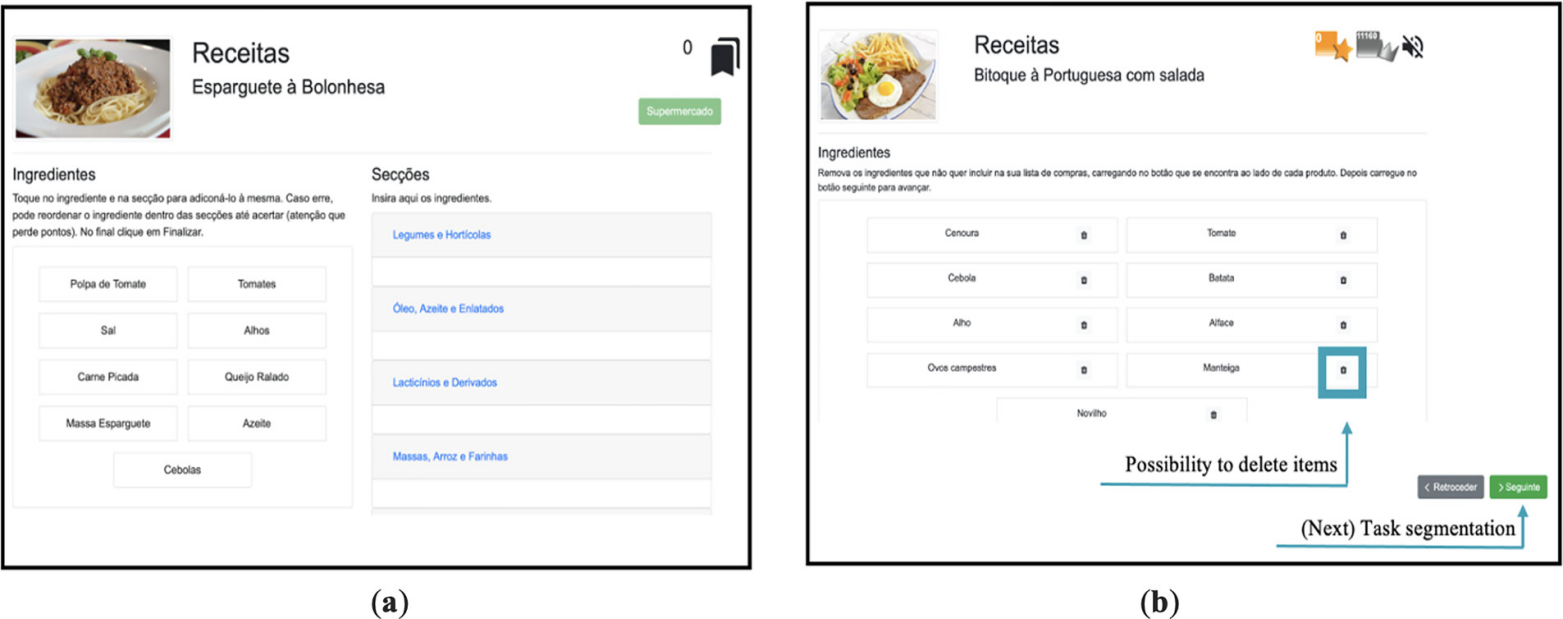
(a) Former recipe game mode interface; (b) the new recipe game mode interface where users can skip subtasks (e.g. task segmentation – organization of the ingredients according to product's category).

**Figure 4.**
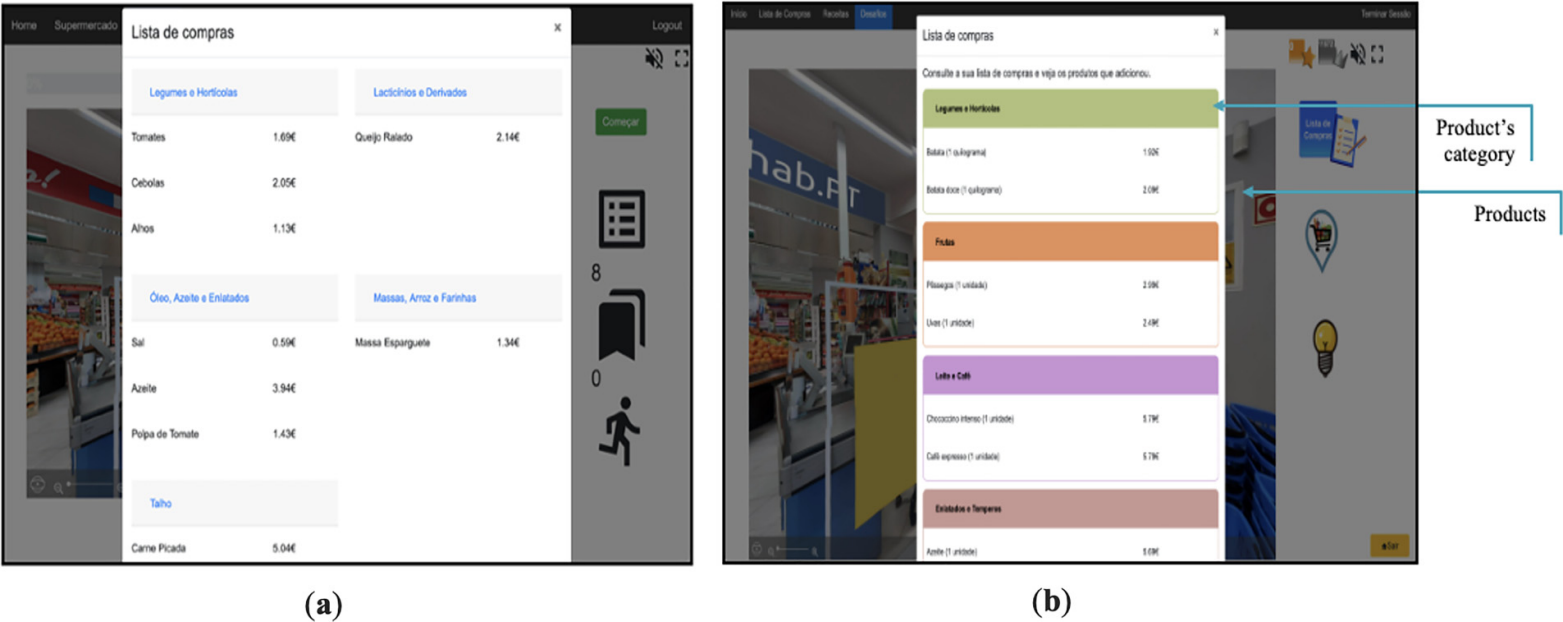
(a) Former shopping list interface with a two-column shopping list; (b) new shopping list interface (after this qualitative study) with 1-column shopping list organised by product's category.

**Figure 5.**
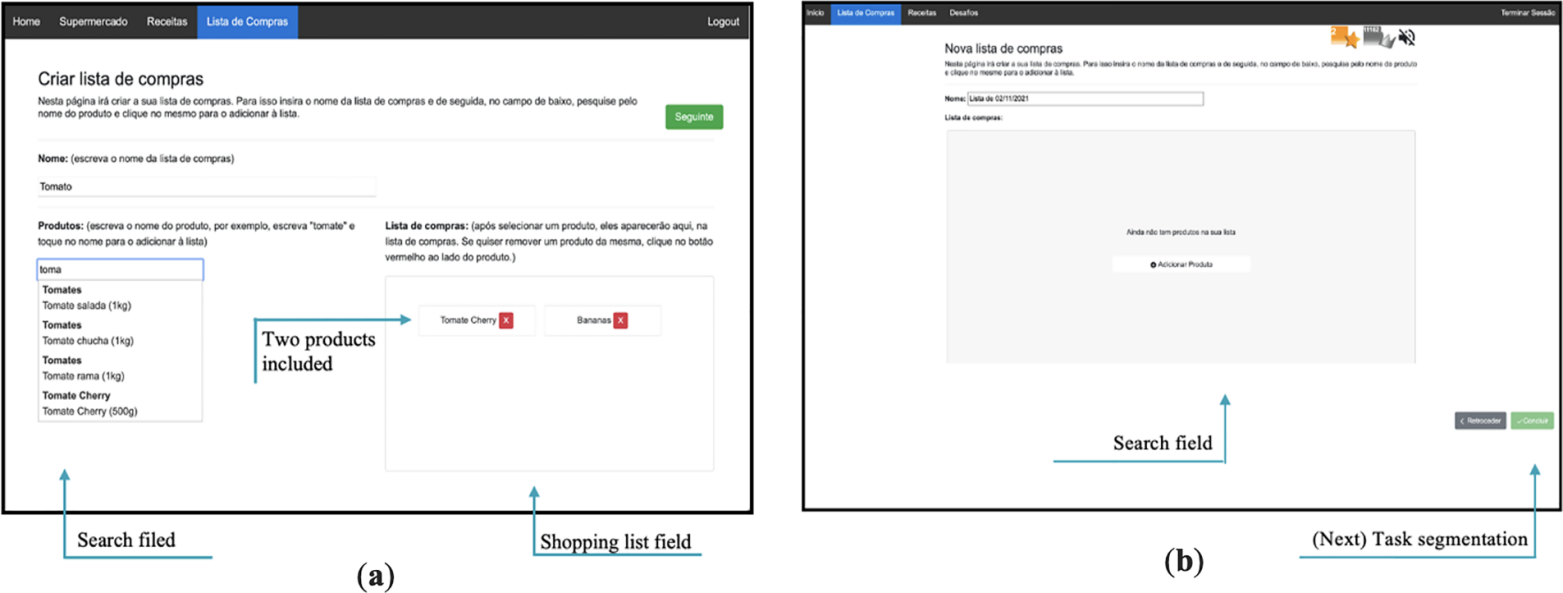
(a) Former shopping list game mode; (b) shopping list game mode with the new features (identified through this qualitative study).

NeuroVRehab.PT is a photo-realistic virtual supermarket designed to promote cognitive functions and functional/behavioural skills involved in shopping activities. The platform was developed using a participatory design approach by a multidisciplinary team of psychologists, neurologists, and computer engineers in collaboration with health professionals and older adults.^16^ The activities incorporated gamification elements such as numeric and non-numeric feedback systems and narrative context to increase users’ engagement and motivation. These activities were grouped into three game modes: supermarket, recipes, and shopping list (see Figures 1(a), 2(a), 3(a), 4(a) and 5(a)).

In NeuroVRehab.PT, users are rewarded with points and positive feedback sounds when they perform correct actions, such as putting a product on the shopping list into the basket. On the other hand, if users perform an incorrect action, such as selecting an item that is not on the shopping list, they receive a negative feedback sound, and no points are awarded. At the end of each level, users are presented with a three-star classification system based on their performance (see Figure 1(a)). For more details about the platform and game modes, see.^16^

### Data analysis

The audio recordings of the interviews were transcribed (see Doc S2 in Supplementary Material) and then cross-checked by the first author (FFB) to ensure the accuracy and removal of any identifiable information (such as participant names, locations, and brands). An inductive-deductive informed Thematic Analysis (TA) was conducted following the six-step approach proposed by Braun and Clarke.^20–22^

Following Braun and Clarke's model, the initial step involves two co-authors (FFB and SA) immersing themselves in the data by carefully reading and re-reading the transcriptions of two randomly selected interviews. An initial set of codes was deductively informed by the research question and interview script. In addition, relevant phrases and recurring ideas were annotated and added to the code list through an inductive process.

For the purpose of analysis, the unit of analysis was defined as blocks of 20 lines within the transcriptions. Codes were modified or added (for all interview transcripts by revisiting the complete set of interviews) as new codes were identified in the remaining transcriptions. Data saturation was achieved during the content analysis, with no new codes identified in the last three interviews. Then, the research team collated text extracts under each potential theme, and codes were organised to fit into the themes’ organising concept. Finally, the research team discussed and refined the final list of themes (and subthemes) and their headings.

## Results

Based on the analysis of the *corpus* of the interviews conducted with eight older adults, three main themes and eight subthemes (Table 3) were identified.

**Table 3.** Themes and subthemes identified based on eight interviews analyzed using a TA protocol.

Interaction experience
Usability and learnability Hedonic experience Confidence, self-efficacy and computer anxiety Reproduction of daily routines
Digital literacy
Computer/tablet skills Gamification and game elements
Attitudes toward NeuroVRehab.PT
Intention to use Utility, usefulness and connectedness

### Theme 1: Interaction experience

*Usability and learnability:* the platform's usability and learnability were determined based on participants’ statements and observed behaviour on their ability to identify the different game modes, follow instructions, and use the game controls. All participants demonstrated the capacity to use the platform independently (to some extent) and could identify and switch between the three game modes. However, some participants initially struggled to navigate inside the virtual supermarket, particularly in understanding the purpose of the arrows as game controls (see Figure 2(a)). For instance, some participants interpreted the arrows as mandatory actions or tips for finding a specific product. After becoming more familiar with the game controls, one participant stated: ‘*… I will not say confusing. But I must train more*” (Participant 5, line 352). To other participants, game controls were ‘*… [not difficult] … as I had the arrows, I would press the arrows, and they would take me where I wanted to go’* (Participant 2, line 412).

Regarding the instructions provided at the beginning of each game mode and throughout the activities, they were considered by participants necessary, important, and easy to follow ‘*… to those who do not know [the platform], it makes sense (…) I think so’* (Participant 2, line 418). Moreover, two participants emphasised the importance of the instructions being in their native language *‘… there were no foreign words at all, it was all in Portuguese’* (Participant 1, line 684), and ‘… *do not put signboards in English (…) I do not know English*…’ (Participant 4, line 753).

*Hedonic experience:* except for one (Participant 5), all participants enjoyed using NeuroVRehab.PT. One of the aspects most valued by participants was the possibility of navigating within a highly realistic virtual supermarket, ‘*… this is interesting, it shows you the supermarket products, the photos of the objects are great…’* (Participant 8, line 419). Another participant echoed this perspective stating,

*… it is interesting the possibility of navigating [within the supermarket] because it motivates you to plan your shopping list in your head. It is interesting. And then you see, I need this [product] while walking through the aisles…. I think it is interesting, yes*!’ (Participant 6, line 650).

On the other hand, Participant 5 claimed, ‘*… to be honest, I did not like it…’* (line 386). To this participant, the absence of ‘people’ in the aisles and checkout counters made the environment look very artificial compared to what happens in the real world.

*… there is no one [in the aisles], the checkout counters are empty (…) maybe [including non-playable characters] would be confusing for the people who will use the platform. But I considered it too artificial; it looked more like a huge freezer than a supermarket*. (Participant 5, line 341), and then added, ‘*… usually, when I go to the supermarket, I think about the time of the day [to go during low affluence periods]. But not having anyone at the checkout counter! … [seems implausible]’* (Participant 5, line 421).

*Confidence, self-efficacy, and computer anxiety:* despite using NeuroVRehab.PT and completing all proposed activities, our results showed that some participants were tense and anxious while using the platform. Some participants had to be constantly encouraged and ensured that everything was all right and that they were not looking bad or doing something wrong: *‘… do not be afraid, you are doing great*.’ (Interviewer speech, Participant 3, line 99) and

*You are doing well, do not worry. You are using something for the first time, and we are all a little like that (…) we are a little unsure, and we have to explore [the platform], we do not know what we are working with, so do not worry.* (Interviewer speech, Participant 1, line 169)

In some cases, the lack of confidence was further compounded by participants’ subjective perception of age-related cognitive decline, fear of memory loss and dementia. As one participant expressed:

*… the problem is me, my memory. If I had a better memory like I used to have in the old days, I would not have forgotten all the details and would have assimilated it all and go straight [to the point].* (Participant 1, line 626)

Moreover, using someone else's device contributed to uneasiness and nervousness for some participants. One participant explained: ‘*[referring to her mobile device] I am not afraid of making mistakes. Okay, that is out, it is not right, let it be (…) I do not cause prejudice to anyone’* (Participant 3, line 432).

*Reproduction of daily routines:* once inside the virtual supermarket, participants’ choices and behaviours seemed to be based on their routines (i.e. real-life behaviours and strategies). For instance, one participant said, ‘*What will I need? Fish, let's follow what I had planned for today [in real life], to buy fish, and then I buy bread…’* (Participant 1, line 36). Other situations where it was possible to observe that participants rely on their usual behaviour patterns to progress in the game were, for instance, when Participant 2 (line 11) immediately tried to pick up a shopping basket upon entering the virtual supermarket without any specific instructions. Another example was when Participant 3 (line 289) was asked how to check the price of a product, and she instinctively reached out to touch the price tag near the item. The similarity between the virtual supermarket and real-world shopping experiences also helped participants monitor the task's progression and determine the following steps to conclude the activity: ‘*I finished the shopping list, right? Now I am going to the checkout counter. I assume this is the same [as in a real-world supermarket]’* (Participant 5, line 235).

However, participants looked puzzled and discontent when the system did not allow them the same freedom of actions and choices (i.e. an accurate reproduction of their shopping-related behaviours). For instance, when specific items were included in a shopping list assigned in one of the Supermarket game levels, one participant exclaimed, ‘*But I do not want bananas…. Ah! Do I have to buy bananas? Oh, okay. Oh, I just wanted the mangos*’ (Participant 4, line 167). A similar situation occurred when participants realised that the system did not allow them to choose the quantity of a product to purchase, ‘*Curry, curry in powder. 50 grams. What if I do not want 50 grams of curry? What if I wanted more or less?’* (Participant 6, line 88) and ‘*bananas … 450 grams, why? What if I want more…?*’ (Participant 6, line 276).

### Theme 2: Digital literacy

*Computer/tablet skills:* despite all participants attending ICT classes, for some of them, this was ‘*… the first time I interacted with a tablet’* (Participant 1, line 625), which presented additional challenges during the evaluation session. The unfamiliarity with tablet devices led to difficulties when specific patterns of interaction were required. For instance, using the keyboard posed a challenge for some participants, as one expressed*,* ‘*… I am not used to this [tablet], if it were on a computer, maybe I would be quicker’* (Participant 4, line 606).

However, the lack of technological proficiency was not limited to tablet use. Participants encountered difficulties with other aspects, such as logging in, closing instruction windows to proceed in the game, and exploring other elements outside the virtual supermarket environment area.

*Gamification and game elements:* our results showed that some participants were not familiar with certain elements commonly found in video games (i.e. game elements; GEs). For instance, when asked about the meaning of the three-star score system (see Figure 1(a)), one participant expressed uncertainty*: ‘That is not décor, for sure! Now, it can be interpreted in two ways … what if there are five stars and I only got three, [and] this orange colour [of the stars] is not good, for sure*…’ (Participant 1, line 348). Other participants had varying interpretations, such as: *‘Three stars is not a lot’* (Participant 5, line 149), ‘*It is sufficient’* (Participant 3, line 399), and ‘*For me, it is [good], I have never done this before, so it is not bad’* (Participant 2, line 372)*.* The lack of familiarity with GEs was also evident when considering the progress bar element (see Figure 2(a)), which was rarely or incorrectly identified: ‘*… this should mean that I already spent 50% [of the budget]’* (Participant 7, line 82).

On the other hand, all participants understood the meaning of the colour-based feedback system used to indicate correct and wrong actions. Participants’ interpretations were consistent, with comments such as, ‘*It turned red (…) It is not right. Something is not right…’* (Participant 2, line 305), and ‘*[when asked if the last action was correct] Yes. (….) It turned green…’* (Participant 1, line 527).

Regarding the auditory feedback system, participants had mixed responses. Some participants correctly identified its purpose, stating, ‘*It tells you [that the product] is already in the basket*’ (Participant 4, line 130), and ‘[it tells you] *It is done!’* (Participant 6, line 284). However, one participant completely missed it: ‘*It went unnoticed … I will tell you why … because this is the computer's sound when (…) an email appears, [when you] do something on the computer*’ (Participant 7, line 395).

### Theme 3: Attitudes toward NeuroVRehab.PT

*Intention to use:* overall, participants present an open and positive attitude towards technology and NeuroVRehab.PT. Regarding NeuroVRehab.PT, participants expressed they were ‘*…curious and want to become more familiar with it’* (Participant 3, line 493). Another participant stated,

*This is interesting … because … how can I explain, this makes us want to explore with the finger, try to find [a product], to rummage [the supermarket], for me, it would be great to be here one or two hours exploring the whole supermarket…’* (Participant 4, line 619).

Moreover, participating in the evaluation session was described as a positive experience, and it motivated them to seek additional opportunities to engage with technology. Another participant expressed ‘…*willingness to buy one of these things [referring to the tablet] …*’ (Participant 3, line 602).

*Utility, usefulness, and connectedness:* It was apparent that some participants were exploring how the use of NeuroVRehab.PT could help them to shop more efficiently. Among the potential practical (utilitarian) benefits identified by participants, one significant advantage was the possibility of completing a pre-existent shopping list. One participant highlighted this aspect by stating, ‘*It could be useful to visit the [virtual] supermarket, to gather ideas (…) maybe I have not even realised that I needed that product. In this aspect, [the platform] is perfect.*’ (Participant 8, line 234). Another participant expressed:

*Being able to create a shopping list with this [while visiting the virtual supermarket], it seems like a good idea. Sometimes, I am at home, [searching for] what I need [to buy], and maybe …, it would come to me [to my mind] easier …. the products that I need (…) The fact that we can visit the aisles… it helps us to identify what we need.* (Participant 6, line 655)

Additionally, one participant further developed this idea by suggesting that the shopping list created in the platform could be e-mailed or printed and then used in a real-life shopping activity: ‘*… this shopping list goes somewhere out? Can I bring it [with me] to the supermarket?’* (Participant 6, line 118).

Another practical aspect identified by participants was the possibility of using the platform to strengthen family bonds, especially with the younger generations. As one participant shared: ‘*Yes. I have grandsons that play (…) they enjoy playing these things. Maybe they would like to play this one too, especially the older one who is 11 years old*’ (Participant 2, line 440).

### Platform improvements and design recommendations

In the previous section, we presented the insights and suggestions provided by participants, which in turn were used to improve NeuroVRehab.PT (see Figures 1(b), 2(b), 3(b), 4(b) and 5(b)). The implemented modifications were thoughtfully selected based on their potential to augment the overall IX with the platform. The changes implemented were categorised into two groups: those directly proposed by participants (i.e. verbally expressed) and those derived from direct observations (e.g. difficulties identified from users’ observed behaviour) (see Table S2 in the Supplementary Material for details).

Based on the analysis of participants’ suggestions and changes implemented, we identified seven design recommendations (Table 4) for developing digital neuropsychological applications targeting low technological proficient populations.

**Table 4.** Design recommendation for low technological proficient populations.

Design recommendations
1. Avoid using technical terms or providing information written in a different language from users’ native language.
2. Ensure users understand that the actions carried out on the platform do not have real-world implications or consequences.
3. The platform's capacity to reproduce the physical, psychological, and cognitive demands of a task when executed in the real world should be considered a crucial aspect during the design and development process.
4. Divide tasks into segments and give users a transparent sense of the task's status, indicating its current phase and whether it has been successfully completed.
5. Introduce new material, tasks, and information periodically to ensure that users continue to feel challenged and engaged.
6. Provide sufficient training on platform/device usage, ideally supported by human guidance before the intervention starts.
7. Whenever feasible, users should have access to the platform on their own devices, rather than on a shared device or a device owned by someone else.

## Discussion

Perceived ease of use and usefulness of technology may be negatively impacted by users’ lack of familiarity with technology and computer anxiety, which consequently hinder the intention to use digital healthcare services.^14,23,24^ In this qualitative study, we sought to explore the feasibility of a digital neuropsychological rehabilitation platform called NeuroVRehab.PT, and the influence of non-clinical factors on IX among a group of older adults with varying levels of technology familiarity and computer confidence.

Our study showed that NeuroVRehab.PT is feasible among older adults with varying computer confidence and computer self-efficacy levels. However, our findings suggest that the continued use of NeuroVRehab.PT may be negatively influenced by participants’ perceived low computer confidence and computer self-efficacy. According to Bandura's Theory of Behavioural Change,^15^ individuals who perceive themselves as less competent in a specific domain are less engaged and more prone to disengage when encountering difficulties. In our study, we observed that participants’ perceptions of their low digital skills and the fear of making mistakes negatively impacted their ability to initiate or complete actions without seeking validation from the researcher. As a result, some participants required continuous encouragement to explore and interact with the platform despite demonstrating the skills and knowledge to do it independently.

After the intervention's initial contact/onboarding phase, continued contact with healthcare professionals has been identified as a promoting factor of adherence to digital interventions.^25^ Together with ours, these findings indicate that human support and guidance are essential for fostering a positive IX and cultivating a sense of patient empowerment when utilising digital healthcare platforms. This notion gains further strength when applied to demographic groups with reduced technological proficiency and low familiarity with digital interfaces.

Our study highlights three other important aspects of designing, implementing, and assessing the feasibility of digital neuropsychological interventions in older adults. First, some of the GEs used in NeuroVRehab.PT were not easily understood by all participants despite being previously identified in other digital neuropsychological interventions.^26^ Video game culture familiarity is gradually increasing among older generations^27,28^ and scientific evidence supports using gamified applications to improve cognitive and other health-related outcomes in older adults.^29^ However, our study calls attention to a different aspect of gamification when applied to the health context, namely the importance of conducting comprehensive analyses to assess gamification elements’ adequacy and differential impact, especially in digital interventions targeting older adults who might be less technologically savvy.

Second, we observed that participants relied on virtual environment (VE) realism to guide their behaviour and progress in the game. The realism of a VE is determined by its ability to simulate both the physical characteristics (i.e. engineering fidelity) and the critical elements that elicit specific behaviours to complete the task (i.e. psychological fidelity).^30^ Based on the reports gathered, we can conclude that NeuroVRehab.PT has a high engineering fidelity derived from its photo-realistic rendered VE. For instance, some participants were naturally compelled to execute specific actions simply by being exposed to virtual stimuli that were sufficiently natural to trigger the usual behaviour (e.g. picking up a basket or checking product prices by clicking on the price tags).

On the other hand, the psychological fidelity of our platform could be enhanced by implementing features that increase the range of routines/behaviours participants usually carry out while shopping. For instance, determining the number and quantity of products included in the shopping list and adding new items to the shopping basket while inside the virtual supermarket were identified as features that would further enrich the IX of our platform.

Finally, we observed a utilitarian use of NeuroVRehab.PT among participants. More specifically, participants demonstrated a strong focus on identifying potential advantages and real-world benefits of using our platform. Notably, participants associated the use of NeuroVRehab.PT with the opportunity to optimise shopping activities by creating or completing their shopping lists before visiting a real-world supermarket. Previous literature has shown that older adults value (and more easily adhere to) technology when it improves and supports daily-life activities.^31^ Although further research is required, these results are particularly encouraging since they suggest NeuroVRehab.PT might contribute to bridging the gap between clinical/experimental context and patients’ daily life routines.

### Limitations and future work

The results of this study provide valuable insights into the impact of nonclinical factors on the IX and feasibility of NeuroVRehab.PT among older adults with varying levels of computer confidence and computer self-efficacy. However, some limitations should be considered when interpreting the findings.

First, it is important to note that the study exclusively enrolled participants attending ICT classes. This may have resulted in a group of individuals more motivated and capable of exploring and interacting with the platform. However, excluding participants lacking basic digital skills allowed us to unmask the influence of computer confidence and self-efficacy on our platform's interaction. This, in turn, contributed to our understanding and awareness of how these factors impact the adoption and adherence to digital healthcare platforms.

On the other hand, for most participants, this study marked their first experience using a tablet device. Consequently, it is challenging to discern whether their motivation stemmed from the platform's ability to engage users or the novelty of interacting with a new mobile device. Future research should explore this aspect by including participants with prior tablet experience or by providing a familiarisation period with the intervention device.

Additionally, the presence of two researchers during the sessions may have contributed to participants’ feelings of nervousness and anxiety. While controlling for this variable is challenging, we attempted to mitigate its potential impact by (1) explaining the role of each of the researchers present, (2) emphasising that it was the platform that was under evaluation rather than the participant, and (3) reinforcing the value of participant's insights regarding their difficulties and doubts in improving the platform.

Lastly, it is important to acknowledge that this study only involved a single session of platform use with a small sample. This limitation hinders our ability to draw conclusions regarding long-term adherence and satisfaction with NeuroVRehab.PT, as well as the influence of individual characteristics on platform use. Future studies should involve recruiting a representative sample and extending the intervention exposure period to assess the feasibility of NeuroVRehab.PT among less technologically-savvy populations.

## Conclusion

The study shows that NeuroVRehab.PT is feasible among older adults with different computer confidence and computer self-efficacy levels. The importance of considering non-clinical factors, such as computer confidence and self-efficacy, when designing and implementing digital healthcare services became evident in our study since these variables seemed to influence participants’ IX negatively. Contact with the healthcare professionals throughout the intervention period, the physical and psychological realism of the VE, and the adequacy of the game elements to the targeted populations seemed to be factors determining the adoption and long-term use of digital healthcare services.

## Supplemental Material

sj-docx-1-dhj-10.1177_20552076231223805 - Supplemental material for Digital health and patient adherence: A qualitative study in older adults Click here for additional data file. Supplemental material, sj-docx-1-dhj-10.1177_20552076231223805 for Digital health and patient adherence: A qualitative study in older adults by Filipa Ferreira-Brito, Sérgio Alves, Tiago Guerreiro, Osvaldo Santos, Cátia Caneiras, Luís Carriço and Ana Verdelho in DIGITAL HEALTH
