# Supplementary material for: Digital health and patient adherence: A qualitative study in older adults
Source: Digit Health. 2024 Jan 12;10:20552076231223805. doi: 10.1177/20552076231223805 (PMC10787534; doi:10.1177/20552076231223805)
Supplement: sj-docx-1-dhj-10.1177_20552076231223805 - Supplemental material for Digital health and patient adherence: A qualitative study in older adults [file sj-docx-1-dhj-10.1177_20552076231223805.docx]

**Supplementary material**

**Table S1.** COREQ (COnsolidated criteria for REporting Qualitative research) checklist.

| **Topic** | **Item No.** | **Guide Questions/Description** | **Reported on**  **Page No.** |
| --- | --- | --- | --- |
| **Domain 1: Research team and reﬂexivity** | | | |
| Personal characteristics | | | |
| Interviewer/facilitator | 1 | Which author/s conducted the interview or focus group? | Page 4 |
| Credentials | 2 | What were the researcher’s credentials? e.g. PhD, MD | Page 4 |
| Occupation | 3 | What was their occupation at the time of the study? | Page 4 |
| Gender | 4 | Was the researcher male or female? | Page 4 |
| Experience and training | 5 | What experience or training did the researcher have? | Page 4 |
| Relationship with participants | | | |
| Relationship established | 6 | Was a relationship established prior to study commencement? | Page 3 |
| Participant knowledge of the interviewer | 7 | What did the participants know about the researcher? e.g. personal goals, reasons for doing the research | Page 4 |
| Interviewer characteristics | 8 | What characteristics were reported about the interviewer/facilitator? e.g. Bias, assumptions, reasons and interests in the research topic | Page 4 |
| **Domain 2: Study design** | | | |
| Theoretical framework | | | |
| Methodological orientation and Theory | 9 | What methodological orientation was stated to underpin the study? e.g. grounded theory, discourse analysis, ethnography, phenomenology, content analysis | Page 3 and 7 |
| Participant selection | | | |
| Sampling | 10 | How were participants selected? e.g. purposive, convenience, consecutive, snowball | Page 3 |
| Method of approach | 11 | How were participants approached? e.g. face-to-face, telephone, mail, email | Page 3 |
| Sample size | 12 | How many participants were in the study? | Page 3 |
| Non-participation | 13 | How many people refused to participate or dropped out? Reasons? | Page 3 |
| Setting | | | |
| Setting of data collection | 14 | Where was the data collected? e.g. home, clinic, workplace | Page 5 |
| Presence of non-participants | 15 | Was anyone else present besides the participants and researchers?F | Page 4 |
| Description of sample | 16 | What are the important characteristics of the sample? e.g. demographic data, date | Page 4 |
| *Data collection* | | | |
| Interview guide | 17 | Were questions, prompts, guides provided by the authors? Was it pilot tested? | No |
| Repeat interviews | 18 | Were repeat interviews carried out? If yes, how many? | No |
| Audio/visual recording | 19 | Did the research use audio or visual recording to collect the data? | Page 5 |
| Field notes | 20 | Were ﬁeld notes made during and/or after the interview or focus group? | Page 5 |
| Duration | 21 | What was the duration of the interviews or focus group? | Page 5 |
| Data saturation | 22 | Was data saturation discussed? | Page 8 |
| Transcripts returned | 23 | Were transcripts returned to participants for comment and/or correction? | No |

| **Domain 3: analysis and ﬁndings** | | | |
| --- | --- | --- | --- |
| *Data analysis* | | | |
| Number of data coders | 24 | How many data coders coded the data? | Page 8 |
| Description of the coding tree | 25 | Did authors provide a description of the coding tree? | No |
| Derivation of themes | 26 | Were themes identiﬁed in advance or derived from the data? | Page 8 |
| Software | 27 | What software, if applicable, was used to manage the data? | n/a |
| Participant checking | 28 | Did participants provide feedback on the ﬁndings? | No |
| Reporting | | | |
| Quotations presented | 29 | Were participant quotations presented to illustrate the themes/ﬁndings?  Was each quotation identiﬁed? e.g. participant number | Page 9 – 14 |
| Data and ﬁndings consistent | 30 | Was there consistency between the data presented and the ﬁndings? | Page 9 – 14 |
| Clarity of major themes | 31 | Were major themes clearly presented in the ﬁndings? | Page 9 – 14 |
| Clarity of minor themes | 32 | Is there a description of diverse cases or discussion of minor themes? | Page 9 – 14 |

*Developed from: Tong A, Sainsbury P, Craig J. Consolidated criteria for reporting qualitative research (COREQ): a 32-item checklist for interviews and focus groups. *International Journal for Quality in Health Care*. 2007. Volume 19, Number 6: pp. 349 – 357

**Doc S1.** Semi-structured interview script

**Interview script^[[1]](#footnote-1)^**

1. **Interaction experience**

- How would you describe your experience with the platform?
- Did you find it easy to understand how the platform functions? What difficulties did you experience while using the platform?
- How clear were the activities presented? Were there times when you felt unsure about the task's objectives or what to do next?
- In terms of entertainment, how would you rate the platform? Is it visually appealing and engaging for users? Can it effectively motivate players to tackle higher difficulty levels?
- Do you believe that the platform's experience closely mirrors the real-life experience of shopping in a supermarket? Are the presented product names, prices, product locations within the supermarket, and the organization of the shopping list realistic? How do you think this experience could be improved? Were there any difficulties identifying products due to brands being out of focus?
- Of the three game modules, which one did you enjoy the most, and which one did you find the most challenging? Why?
- How interesting and enjoyable to play are the time-limited levels in the?
- Do you think the execution time and level of difficulty in different levels are appropriate, or do they need adjustment?
- While using the platform, did you experience any discomfort, such as dizziness, nausea, blurred vision, headaches, or any other symptoms?

1. **Instructions and feedback**

- Regarding the length of the dialogue boxes, the language used, size, and font, do you find them appropriate and readable?
- How precise did you find the instructions provided by the platform? Do you think they should be more detailed?
- How valuable do you find the use of images from a real supermarket, such as the types of products you find in your local store, in terms of the interaction with the platform?
- How is the navigation aspect? Does navigating within the supermarket feels natural to you, or do you feel it should be faster or slower?
- How intuitive are the icons, images, and sounds used in the platform? Can you easily understand the function and meaning of each one?
- How would you describe the visual aspects and organization of the game screen: organized, clear, or confused?
- Do you feel that the feedback provided, both when you get things right and when you make mistakes, is sufficient and clear?
- Is the tab with the product’s information important for you or a potentially destabilizing element?
- How important is the activity of organizing the shopping list?
- How clear and understandable is the feedback given at the end of each level session?

1. **Intention-to-use**

- Could incorporating this platform into your daily routine enhance the efficiency and speed of your shopping?
- If available to the public, will you consider using the platform on a daily/weekly basis? Why?
- How likely and easy would it be for you to use this platform in your home, particularly when you are alone?
- How comfortable would you feel using this platform in public settings, such as with friends and family?
- Would you recommend this platform to a family member or friend?
- If your doctor recommended you use this platform to stay active, would you be inclined to follow their advice? How frequently would you use this platform? Would it be easy to include it in your routine?

1. **Final remarks**

- What platform features did you enjoy/value most?
- What other elements could we introduce to enhance the platform's engagement and bring it closer to the real supermarket shopping experience?
- Are there other aspects or features that you believe are important or interesting to include on the platform? Do you have any specific suggestions you'd like to share with us?

**Doc S2**. Transcription of the Interviews Conducted (n = 8)

*Participant 1*

**Mod. – Está descansado, não é. Pronto, quando quiser, pode começar a mexer, explorar. Não se preocupe, pode carregar em todo o lado que isto não estraga.**

**M. –** É o normal.

**Mod. – Pode começar a andar.**

**M. –** É isso?

**Mod. – Vamos para cima.**

**M. –** é preciso ler ou não é preciso?

**Mod. – Se quiser, senão…**

**M. –** Devia ter trazido óculos…e agora? Lista de Compras.... Abro isto?

**Mod. – Pode ser. Está a ir muito bem. Mas aqui teria de carregar no…**

**M. –** Neste?

**Mod. – Exatamente.**

**M. –** Selecione o nível para jogar. Terá de ser o mais baixo. Mesmo assim terei alguma dificuldade.

**Mod. – Está a ir muito bem.**

**M. –** E agora estou aqui no…

**Mod. – Olhando para o resto…**

**M. –** Estamos aqui no mercado.

**Mod. – Exato.**

**M. –** O que é que eu vou precisar daqui. O peixe, já agora, seguir o meu sentido de hoje, comprar peixe, depois fui comprar pão… não sabia onde estava a prateleira do peixe e do pão.

**Mod. – Olhando para esta imagem, parece-lhe que está aonde?**

**M. –** Numa frutaria, não é? as frutas…

**Mod. – E olhando para o resto do ecrã, vê alguma coisa que lhe permita, que lhe dê indicação… de que…**

**M. –** Que não tem a ver com uma frutaria?

**Mod. – Não, não. No resto do ecrã, por exemplo, se quisesse começar este nível de jogo... vê algum botão que seja indicativo de que tem de carregar aqui para começar a fazer?**

**M. –** Aqui?

**Mod. – Exatamente.**

**M. –** Bem-vindo ao nosso supermercado virtual. O meu nome é Maria, vou ser a sua assistente de compras, para ver a próxima página carregue no botão que aparece em baixo (…) fazer as suas compras (...) Uma seta?

**Mod. – Sim.**

**M. –** Aqui.

**Mod. – Tem de fechar, faça continuar como estava a fazer. E agora, vê alguma seta no ecrã? Exatamente, experimente carregar.**

**M. –** [o entrevistado murmura o texto] O que se pretende? Continuar?

**Mod. – Sim.**

**M. –** Esta setazinha aqui.

**Mod. – Exato.**

**M -** [o entrevistado murmura o texto] vai encontrar legumes.

**Mod. – Terá que fechar primeiro…**

**M. –** Continuar?

**Mod. – O balão, exato. Carregar continuar. Acho que não…**

**M. –** Agora não sei… e ao mesmo tempo deslize para a direita para encontrar a seta. [o entrevistado murmura o texto]

**Mod. – Botão continuar, para fechar.**

**M. –** Pensei que bastava carregar…

**Mod. – Podia ser. ainda se lembra da instrução, que era?**

**M. –** Acho que era Frutas do lado direito, legumes do lado oposto.

**Mod. – Exatamente. Agora para encontrarmos a próxima seta, era carregar no centro do ecrã, e deslizar o dedo para a direita ao mesmo tempo. Exatamente. Até encontrar a outra seta.**

**M. –** Está aqui.

**Mod. – Sim. pode carregar. Mesmo em cima dela. assim.**

**M. –** É a mesma coisa, não é?

**Mod. – Agora experimente carregar nas maçãs.**

**M. –** Um euro e 52, não estão muito caras. Adicionar ao cesto.

**Mod. – Experimente carregar, para ver o que acontece.**

**M -** [o entrevistado murmura o texto] Mas as maçãs não constam na lista?

**Mod. – Não.**

**M. –** Adicionar (…) Mangas do lado esquerdo, bananas do lado direito. Isto está correto. Continuo?

**Mod. – Agora experimente carregar numa?**

**M. –** 450 gramas, 1,36. Não dá para saber quanto custa um quilo, não?

**Mod. – Pois…**

**M. –** É?

**Mod. – Não.**

**M. –** Ah, pensei que fosse. Antigamente fazia-se logo de repente. O meu trabalho foi sempre… agora o que se pretende então?

**Mod. – Experimente carregar no… adicionar ao cesto, para ver o que é que acontece? E agora tinha uma outra…fruta.**

**M. –** Fruta. Que era as mangas do lado esquerdo.

**Mod. – Do lado esquerdo, consegue identificá-las? Experimente novamente.**

**M. –** 2,56… não sei se o objetivo é esse. Continuo?

**Mod. – Sim, sim.**

**M -** [o entrevistado murmura o texto] Continuo?

**Mod. – Já comprou as duas frutas, agora quer… como se fosse um supermercado normal, ir pagar. ir até às caixas, exato.**

**M. –** E agora se calhar ver qual era o preço das coisas, não? Antes de ir à caixa de pagamento… carregue no botão… no canto superior direito. Aqui é o canto superior direito?

**Mod. – Primeiro tem que fechar esse balão, esta caixinha, sim.**

**M. –** Fecho aqui? Aqui no continuar?

**Mod. – Exatamente.**

**M. –** A seguir escolha o modo de pagamento…

**Mod. – Agora temos de entrar na caixa. Temos sorte, não temos ninguém à nossa frente [risos]**

**M. –** Boa, o que é raro. E é mau para eles, não estão a vender bem, coitados.

**Mod. – Também ainda é cedo, ainda é cedo. Agora tem que pagar. E no balão dizia que tinha de carregar no botão pagar que ficava no canto superior direito.**

**M. –** Direito. Então, é aqui agora?

**Mod. – Veja nessa… pensando no *tablet,* todo, o canto superior direito.**

**M. –** Ah. Está aqui o pagar.

**Mod. – Exatamente.**

**M. –** Escolha o modo de pagamento, MB, dinheiro… ainda sou à moda antiga, vou pôr dinheiro [o entrevistado murmura]

**Mod. – Passamos ao próximo nível, jogamos mais um?**

**M. –** Por mim, estão à vontade. Não sei se você tem paciência, reconheço que estou a ser um bocado…

**Mod. – Está a ir bem, não se preocupe. Está a conhecer uma coisa pela primeira vez e todos somos assim. vamos um bocadinho…**

**M. –** Pois.

**Mod. – A medo e temos de ir experimentado, não sabemos com aquilo que estamos a trabalhar, não se preocupe. E está a sair-se lindamente, é precisamente isto que, ah, nós pretendemos. Ver alguém a experimentar a nossa plataforma pela primeira vez.**

**M. –** Portanto, vamos ao nível dois?

**Mod. – Vamos lá.**

**M. –** Começar. Clicar novamente…

**Mod. – Carregar outra vez, ele não reconheceu.**

**M. –** Ora bem, eu vou escolher… está aqui a setazinha… isto se não for diabético, se for é demais.

**Mod. – Pensando na lista de compras, que estava ali no balãozinho e, novamente, olhando para o *tablet* todo. Há algum símbolo que o faça pensar que ali será a lista de compras?**

**M. –** Hmm… hmm… não.

**Mod. – Muito bem, experimente carregar aqui neste.**

**M. –** Não é para memorizar os preços?

**Mod. – Não, não, não se preocupe.**

**M. –** Tangerinas. Saio?

**Mod. – Sim.**

**M. –** Vamos continuar. [o entrevistado murmura o texto] Toco no ecrã?

**Mod. – Exatamente. Mas primeiro temos de fechar o botãozinho. Agora, como há pouco.**

**M. –** Até encontrar… vamos lá ver, não fixei. Ali tudo bem, era deslizar para a direita até encontrar…

**Mod. – Até encontrar a seta…**

**M. –** Até encontrar a seta, ok. [o entrevistado murmura o texto] É clique…

**Mod. – E pode como há pouco, pode mexer na imagem, para ver onde é que elas estão.**

**M. –** E paro a imagem lá?

**Mod. – Para a imagem onde se sentir mais confortável, onde for melhor para si.**

**M. –** Ou na seta ou vou ao produto as mangas, meloas e tangerinas.

**Mod. – Eu vou dar uma ajuda, vamos para aqui, elas estão aqui nesta zona. Agora, novamente, pode mexer à sua vontade.**

**M. –** Até encontrar mangas, meloas e tangerinas.

**Mod. – É como se estivesse a entrar num supermercado novo, não é, e antes de encontrar o produto que quer, está a olhar para ver se o identifica.**

**M. –** Ok. então, à partida está aqui a setazinha, deve ser.

**Mod. – Portanto, quando vê uma seta o pensamento que tem é, ok, se está aqui esta seta é para eu ir nesta direção?**

**M. –** Sim, sim.

**Mod. – OK. não vê nenhuma das frutas que…**

**M. –** Mangas não estou a ver, meloas… meloa sim. e tangerinas. Isto parecem ser tangerinas. É muito pequenina, mas pronto.

**Mod. – Clique aqui mais ao centro. Temos de nos chegar um bocadinho ao pé delas, agora desliza novamente a imagem…**

**M. –** Mangas já estou aqui a vê-las. Tanto ser uma como a outra, umas são do brasil e as outras são de angola, se calhar.

**Mod. – Pode então já colocá-las no seu cesto. Afinal não eram mangas.**

**M. –** Eish, pois não.

**Mod. – Agora se quisesse fechar.**

**M. –** Fechar, supostamente será aqui. Não sendo estas, terão de ser estas.

**Mod. – Experimente para ver o que ele diz.**

**M. –** Mangas, é o único…então, adicione ao cesto.

**Mod. – Exato...acho que ele não identificou. Este som que ouve, o que lhe diz?**

**M. –** Qualquer anomalia.

**Mod. – Qualquer coisa que…**

**M. –** Não está correta. Assim sendo, volto aqui?

**Mod. – Pode voltar.**

**M. –** Manga… já está.

**Mod. – A manga já está.**

**M. –** Agora vou tentar identificar a meloa e a tangerina.

**Mod. – Exatamente.**

**M. –** A meloa deve ser mais fácil. É sim senhora. Meloa, adicionar, tangerina. À falta de outra fruta, parece-me ser esta aqui.

**Mod - Experimente, para ver o que ele nos diz.**

**M. –** [o entrevistado murmura o texto] adicione à lista de compras (…) dirija-se à caixa. Dirija-se à caixa e carregue no botão pagar.

**Mod. – Neste momento qual é, que dúvida lhe está a surgir?**

**M. –** Como é que eu vou para a caixa.

**Mod. – Não está a ver nenhum elemento que o ajude, é isso?**

**M. –** Não.

**Mod. – Eu vou ajudar, vou carregar e virar a imagem.**

**M. –** Altamente…

**Mod. – E agora tem duas setas. Não é?**

**M. –** Sim, tenho de optar por uma…

**Mod. – Exato.**

**M. –** Vamos lá ver.

**Mod. – Aqui é a zona dos legumes, não é isso que nós pretendemos. Se formos para ali, vamos para o sítio de onde viemos, também não queremos. Resta-nos… essa primeira, não é?**

**M. –** Ok. e aqui estamos nós… não está cá a senhora, podemos passar sem pagar [risos]

**Mod. – Aproveitar [risos]**

**M -** [o entrevistado murmura o texto]. Frutas, legumes, hortícolas… [o entrevistado murmura o texto] encontrar caixas (…) [o entrevistado murmura o texto]

**Mod. – está indeciso entre quais?**

**M. –** Entre estas duas últimas. Acho que nós vamos para as frutas… hortícolas acho que não… não foi tanto.

**Mod. – Experimente, vamos ver o que acontece. Experimente novamente.**

**M. –** Entrar nas frutas…

**Mod. – Vendo que o quadrado se iluminou a vermelho, o que acha que lhe está a dizer?**

**M. –** Que não está correto.

**Mod. – Não está correto. Então vamos experimentar a nossa segunda opção. Que era? E este som?**

**M. –** Sim, este som…

**Mod. – Já parece.**

**M. –** Já parece que está correto.

**Mod. – Ok, vamos terminar as nossas compras.**

**M. –** ok, vamos pagar. Exatamente isso fica bem…

**Mod. – E agora sim… tem que pagar.**

**M. –** Pagar. escolha o modo de pagamento, vamos optar por dinheiro.

**Mod. – E este som que está agora a ouvir, é diferente dos anteriores?**

**M. –** Sim.

**Mod. – O que acha que lhe está a tentar dizer?**

**M. –** Se quero continuar, outra opção.

**Mod. – E estas estrelinhas que estão aqui, dizem-lhe alguma coisa ou acha que é apenas um elemento decorativo?**

**M. –** Isso decorativo não é de certeza, não é? agora pode ter duas interpretações, não sei… se o nível são cinco e eu só consegui 3, esta cor alaranjada não é do melhor, de certeza, pelo menos em termos meteorológicos não é.

**Mod. – Então, para ser fantástico, teria de ser assim um verde, era essa?**

**M. –** Sim.

**Mod. – Eu agora vou sair, vou fazer próximo nível. E olhando agora para este ecrã e pensando nas estrelas, já consegue perceber qual era a cotação máxima?**

**M. –** Sim.

**Mod. – No máximo cada nível consegue ter… três estrelas, não é? e tem conseguido…**

**M. –** E com cores diferentes.

**Mod. – Estas aqui, estando…**

**M. –** Ainda não foram usadas.

**Mod. – Os dois níveis que fez conseguiu o máximo da pontuação.**

**M. –** Tem que ser muito fácil para eu conseguir isso.

**Mod. – São os primeiros níveis, é nível de adaptação. Gostava de lhe mostrar mais dois módulos de jogo que esta plataforma tem. Podemos avançar. O segundo, vou-lhe pedir, olhando para o menu de cima, que explore. Ou seja, carregue e veja o que acontece. Se** **tivesse…**

**M. –** Só em cima?

**Mod. – Sim, na barrinha preta.**

**M. –** Mas escolher uma coisa que eu queira daqui?

**Mod. – Sim.**

**M. –** Por exemplo…

**Mod. – Sim.**

**M. –** Mercado é onde nós estamos.

**Mod. – Exato.**

**M. –** Receitas deve ter a ver as coisas das senhoras, os bolos, receitas… ou serão receitas dos comerciantes.

**Mod. – Não, é bolos…**

**M. –** A língua portuguesa é tramada. Vamos à lista de compras

**Mod. – Mas podia ser, receita do final do dia ou do final do mês.**

**M. –** Os comerciantes, e tem de declarar às finanças e há uma preocupação com aquilo, vão fugir.

**Mod. – Experimente, ou as receitas ou a lista de compras.**

**M. –** Vamos ver a lista de compras, se calhar. Selecione a lista de compras para jogar. Esta… onde é que esta está… para aqui? Estar aqui… tem alguma coisa a ver com isto?

**Mod. – isto o 52 é uma coisa… ignore. É só aqui o debaixo, por favor dê um nome à sua lista.**

**M. –** Lista de compras?

**Mod. – à sua lista de compras. Para fechar esta janela teria que… exato.**

**M. –** Ok. escreva um nome na lista de compras. Um nome aleatório?

**Mod. – Pode ser qualquer coisa, o dia de hoje. A data de hoje, por exemplo…**

**M. –** Então...

**Mod. – Exatamente.**

**M. –** Hoje são 20… de janeiro ou 20 janeiro?

**Mod. – Como quiser.**

**M. –** Onde é que está a barra.

**Mod. – Está aqui.**

**M. –** 20, 20.

**Mod. – Agora para continuar a sua lista de compras e começar a escrever os produtos que eventualmente quisesse, como é que faria?**

**M. –** Lista de compras… [o entrevistado murmura o texto] será aqui? Tenho de selecionar os produtos.

**Mod. – Experimente clicar, para ver o que acontece.**

**M. –** [o entrevistado murmura o texto] tomate.

**Mod. – Para apagar é nesse....**

**M. –** Tomates, pronto, ok.

**Mod. – Sim, vamos supor que era isso que queria.**

**M. –** Só tenho 11…

**Mod. – Se quisesse outro produto.**

**M. –** Outro?

**Mod. – Outra coisa, se quisesse fazer uma salada.**

**M. –** Ok, alface por exemplo.

**Mod. – Por exemplo.**

**M. –** Alface… e tenho que…

**Mod. – Tem que lhe dizer, é isto que eu quero.**

**M. –** Mas ele não está a assumir. Está? Não…

**Mod. – Consegue andar um bocadinho para cima no ecrã? Exatamente. Olhando para aqui…**

**M. –** O botão vermelho, produtos…

**Mod. – Vou tirar aqui o teclado para conseguir ler a mensagem completa.**

**M –** [o entrevistado murmura o texto] desapareceu da sua lista de compras.

**Mod. – Pode andar para cima e para baixo no ecrã, para ver se lhe surge.**

**M. –** ok deixe lá ver. Porque está a vermelho?

**Mod. – Vamos experimentar. O que aconteceu?**

**M. –** O tomate saiu.

**Mod. – O tomate saiu. Portanto, o que é que isto, o que é que acha que aconteceu?**

**M. –** Qualquer coisa que eu fiz mal. Ou na seleção ele não assumiu.

**Mod. – Vamos para o tomate… ele aparece aqui novamente.**

**M. –** OK.

**Mod. – Esta cruzinha… vermelha, é quando... imagine que queria ter, comprar tomates, mas, entretanto, lembra-se que afinal ainda tem lá em casa e já não quero. E tira, é da sua lista de compras, não é que tenha feito alguma coisa mal, mas é esta opção de não precisarmos, vamos tirar da lista.**

**M. –** Então… tira.

**Mod. – Pronto. Para a lista…**

**M. –** Alface também não.

**Mod. – Não precisa para hoje.**

**M. –** Pelos vistos, ele está a dizer que tenho em casa.

**Mod. – Não, isto é, caso não queira continuar.**

**M. –** OK.

**Mod. – OK, mas vamos assumir que hoje vamos querer alface, precisamos de alface. Já temos a nossa lista de compras, que… hoje não é preciso uma lista muito grande, é só um item. Se eu quisesse agora ir ao supermercado, como é que eu faria, utilizando a plataforma para ir ao nosso supermercado que há pouco vimos. Teria… experimente andar, exato,** **com o ecrã.**

**M. –** A seguir?

**Mod. – Por exemplo.**

**M. –** Há aqui os corredores. Tenho que os inserir, também?

**Mod. – Agora aqui, isto é um bocadinho diferente. Diz que… experimente tocar num. Ele ficou a tracejado, verde. Isto diz o quê?**

**M. –** Está à espera de mais alguma informação.

**Mod. – E se olhar para a parte… experimente.**

**M. –** OK.

**Mod. – Parece-lhe que ele aceitou?**

**M. –** Sim.

**Mod. – E porque é que acha que aceitou?**

**M. –** Está a verde, qualquer sinal sonoro… supostamente está.

**Mod. – Agora se quiséssemos ir, se isto fosse uma situação real e quiséssemos ir para o supermercado, aqui como é que eu faria para ir para o supermercado?**

**M. –** Estamos…

**Mod. – Vamos só comprar as alfaces para terminar?**

**M. –** Bora.

**Mod. – Então vamos lá.**

**M. –** acho que tenho de ir aqui aos produtos. Não, começar para já, não é?

**Mod. – Exatamente.**

**M. –** Estava a gerar, estava a ver se ia logo… em princípio há de estar aqui deste lado, quer dizer, deveria estar. Se a loja for organizada, se não for, se for dos (informação sensível) está em qualquer lado. aqui… não me ajuda muito não é, tenho de identificá-la.

**Mod. – Tem de andar pelo supermercado à procura dela.**

**M. –** Pois, à procura dela.

**Mod. – Para andar pelo supermercado, como é que… se eu quisesse seguir em frente.** **Tenho esta possibilidade de…**

**M. –** Se quisesse seguir em frente, iria assim, mas eu quero localizar a alface, não é… isso é que não estou a ver. não estou a ver as alfaces, supostamente estariam nesta prateleira, não é? acho eu…

**Mod. – E utilizando a seta.**

**M. –** Já vi…

**Mod. – Já estamos um bocadinho mais perto. Mas ainda assim…**

**M. –** Quer dizer, aqui supostamente não parece que possa…

**Mod. – Vamos ver, vamos chegar mais á frente e vamos ver.**

**M. –** As alfaces…

**Mod. – OK, tudo isto é… é uma possibilidade. Eu aqui vou-lhe dar uma ajuda porque elas de facto estão um bocadinho escondidas. Elas estão ali ao fundo.**

**M. –** Vê-se mal.

**Mod. – Experimente, experimente. Mas como ainda estamos muito longe, temos de nos chegar até elas e só depois vamos conseguir colocá-las no nosso cesto. Se nós quiséssemos voltar à imagem inicial, como é que… certo! Está ótimo. Agora vamos chegar-nos à frente? Utilizando a setinha que estava lá em cima… temos agora que fazer aquela… de, exatamente, de… deslizar. Até encontrarmos as alfaces que vão estar aqui do nosso lado esquerdo.**

**M. –** Alface… adicionar ao cesto. [o entrevistado murmura o texto]

**Mod. – Temos agora de ir até à caixa.**

**M. –** Era isso que agora estava a tentar ver. finalizar? já cliquei no finalizar e ele não assumiu. Ou vou por aqui até ele encontrar a caixa?

**Mod. – Exato. Pode seguir, exatamente, pode seguir em frente. E agora aqui posso-lhe dar uma ajudinha, como ainda não conhece o supermercado…**

**M. –** A setazinha.

**Mod. – Exato.**

**M. –** Não ajudou muito. Cá está ela.

**Mod. – Agora temos de ir mais um bocadinho, temos de entrar mesmo na caixa.**

**M. –** Assim não… assim é a caixa…

**Mod. – Para andar utilizamos sempre as setinhas, não é, entretanto ela escondeu-se aqui…**

**M. –** Já cá estamos.

**Mod. – Ver mais um bocadinho. Agora tem aí outra…**

**M. –** Vamos para esta.

**Mod. – É o bom para este supermercado, não há filas. E agora já estamos na caixa e vamos querer pagar.**

**M. –** OK… pagar… por favor escolha o meio de pagamento. Dinheiro.

**Mod. – Pronto, fez a sua lista de compras, era só um item, mas fez com a pontuação** **máxima. Senhor (informação sensível) agora vou-lhe fazer as tais perguntinhas, está bem?**

**M. –** Está bem.

**Mod. – E, mais uma vez, é dar-nos a sua opinião, de forma mais imparcial possível, de forma a ajudar-nos a identificar o que é que nós podemos e devemos melhorar na nossa plataforma para que ela seja mais fácil de utilizar. E a primeira pergunta que eu gostava de lhe fazer é, que dificuldades é que sentiu? De uma forma geral, o que é que para si foi mais difícil na plataforma?**

**M. –** Olhe, foi a primeira vez que eu trabalhei com um *tablet*. Logo aí, resultou algumas dificuldades. E, depois, um problema que é meu, em termos de memória. Se eu tivesse facilidade maior de memória, não é, como tinha antigamente, facilmente não me teria esquecido daqueles pormenores, teria assimilado todos e iria diretamente.

**Mod. – E onde é que sentiu essas dificuldades de memória?**

**M. –** Quando é que comecei a sentir?

**Mod. – Não, não, aonde, aqui na plataforma?**

**M. –** Deveria ter ido mais rápido aos assuntos. Deveria ter ido logo, e senti alguma dificuldade a encontras as coisas.

**Mod. – E aquela questão de andarmos com… através das setas, este mecanismo de navegação que nós utilizamos aqui, para si foi intuitivo, fácil. Ou de que forma é que poderíamos fazer isto de forma mais fácil, intuitiva.**

**M. –** acho que está bem pensado e não é difícil. Eu é que tive alguma dificuldade em compreender aquilo logo. Agora com continuação seria muito fácil.

**Mod. – Em algum momento, por exemplo, no primeiro nível quando nós tivemos que compras as bananas e as mangas, em algum momento sentiu-se perdido sem saber o que tinha de fazer?**

**M. –** Tive alguma dificuldade em localizar as coisas, portanto…

**Mod. – Mas sempre soube o que era para fazer?**

**M. –** Isso sim.

**Mod. – Era mais a questão de…**

**M. –** Da frutaria, as coisas, localizá-las.

**Mod. – Tal e qual como quando acontece quando vamos a um sítio novo, aquele primeiro momento, podermos perceber onde é que estão as coisas. este supermercado, enquanto estava lá, sentiu que era próximo daquilo que sente quando vai a um supermercado no seu dia a dia? O ambiente é semelhante?**

**M. –** Tenho que dizer a verdade. Eu vou pouco. Normalmente essas tarefas estão, pelo menos, destinadas à minha mulher. Mas pronto, de vez em quando também vou. Mas acho que a organização dele era bom. Estava mais ou menos.

**Mod. – Era um aspeto natural? Era… era algo que esperaria encontrar no seu dia a dia se entrasse num supermercado.**

**M. –** E com frutas e legumes à vista, portanto, agradáveis. Aparentemente frescos, coisas frescas, não está lá o peixe.

**Mod. – Está mais para o fundo [risos]**

**M. –** Se calhar.

**Mod. – e houve esta situação de não ter trazido os óculos. Mas tirando isso, sentiu em algum momento, por exemplo, náuseas, tonturas, quando virava a…**

**M. –** Não, não.

**Mod. – Não sentiu nada disso?**

**M. –** Não, não.

**Mod. – A linguagem, por exemplo, aquelas caixas de texto que apareciam, a dizer o que era para fazer, onde é que estava a lista de compras, elas eram fáceis de entender, a linguagem era adequada ou acha que poderíamos utilizar uma linguagem mais simples?**

**M. –** Penso que sim, por aí… não havia ali estrangeirismos nem nada, era tudo em português.

**Mod. – Era fácil, não acho que os textos fossem muito compridos?**

**M. –** Não, antes pelo contrário.

**Mod. – E esta questão de organizar a lista de compras, como nós fizemos, acha que é uma tarefa útil, quando nós queremos otimizar…**

**M. –** Isso de certeza, de certeza. De certeza.

**Mod. – E o que achou desta situação de nós querermos o produto e depois agarrávamos o produto e inseríamos dentro da categoria. Isto faz-lhe sentido ou seria mais fácil irmos escrevendo a lista, os produtos todos na lista e seguirmos logo para o supermercado?**

**M. –** O supermercado também é muito pequeno não é, não era nenhum (informação sensível). Mas pronto, tudo o que seja organização, é útil, facilita. Pelo menos não nos esqueçamos de algumas coisas. Esta ali, é ir seguindo a lista, ir picando, está tudo, está tudo, não falta nada, pagar.

**Mod. – E é fácil perceber na plataforma quando é que nós já temos aquele produto?**

**M. –** Ah sim, isso basta ver também. Se estiver picado…

**Mod. – Quando diz picado?**

**M. –** Selecionado.

**Mod. – OK. e agora pensando num outro contexto, vamos imaginar que o senhor (informação sensível) queria começar a fazer compras neste supermercado. Sentia-se à vontade para utilizar esta plataforma, por exemplo, em sua casa e ir treinado?**

**M. –** Ir treinando… tinha que treinar muito para ser útil, chegar ali e ser rápido. Senão tinha de andar lá com isto e andava perdido o dobro do tempo.

**Mod. – E…**

**M. –** Teria de treinar.

**Mod. – Mas sentia-se à vontade para o fazer em casa, num tempo…**

**M. –** com tempo sim, explorava isto mais um bocadinho e sim, isso conseguia.

**Mod. – E em público com os seus amigos? Sentia-se à vontade, constrangido?**

**M. –** Não tinha problema quanto a isso. isso… acho que funcionava da mesma maneira.

**Mod. – E, por exemplo…**

**M. –** Preferia, com os meus amigos, andar à procura dum carro ou de um barco ou de uma coisa desse tipo.

**Mod. – Que tipo de tarefas é que gostava de ver assim dentro deste género. Estava agora a falar dos carros, vê carros, era isso?**

**M. –** Por exemplo, é uma coisa que eu gosto. Passo bastante tempo na internet, normalmente é à procura de carros, essas coisas que eu gosto.

**Mod. – E aqui vai ter que me ajudar a mim, eu não percebo muito de carros. O que procura quando anda à procura de carros, é só ver o carro por fora?**

**M. –** Ver tudo, ver preços. Não é que eu faça vida disso, mas já fui buscar 3 ou 4 carritos à Alemanha. E gosto de estar mais ou menos atualizado, com os preços e assim. e vou a um site ou dois onde estão lá 1 milhão e 500 mil carros. E pronto, ali faço a seleção.

**Mod. – e vai vendo.**

**M. –** Escolho a marca. (informação sensível), vai-me dar (informação sensível), modelo, ponho o modelo de carro, o ano, os km até, e depois selecionamos e vemos uma série de carros que lá estão.

**Mod. – Outra tarefa, tem mais alguma tarefa ou atividade que gostasse… ou que goste de ir à internet e assim nos seus tempos livres…**

**M. –** Não, das coisas que eu vejo muito é o tempo. Que ocupa muito o meu tempo, porque eu tenho um hobby que é quase uma profissão, mas enfim, que é a pesca. Sempre que posso, vou para a pesca. E, então, gosto de estar…

**Mod. – Faz sentido saber o tempo.**

**M. –** Faz todo o sentido. Já pesco há muitos, muitos anos, e era frequente irmos à pesca e depois de lá estar, termos de vir embora porque o tempo não deixava ou o mar estava muito mau. Porque faço pesca em barco. Então, tinha de vir embora. E agora, com a informação que nós temos, é raríssimo isso acontecer. ou seja, só vou para o mar quando o tempo deixa, quando não chove, consigo ter essa informação toda. Muita informação que hoje temos. E perco tempo a ver isso, para ver quando devo ir e tal. Perco um bocado de tempo. Perco ou ganho um bocado de tempo. Gosto de estar mais ou menos atualizado.

**Mod. – Uma última pergunta que eu lhe queria fazer. imagine que tem um familiar seu que começa a apresentar algumas dificuldades de memória, esquecimentos, acha que a sua plataforma poderia ajudar com… para exercitar, por exemplo a memória e estas tarefas relacionadas com a ida às compras?**

**M. –** Eu até lhe posso dizer, nem lhe vou relacionar com… tenho um familiar meu, mas comigo mesmo, acho que sim, isto é útil. Isto, acho que… a gente possa exercitar o cérebro, é importante. Para mim e para os meus familiares também. E eu estou nessa fase com alguma preocupação. Isto do Alzheimer toca a todos, uns mais rápido do que outros. Uns mais cedo. E infelizmente ou felizmente, eu contacto frequentemente com lares de idosos (informação sensível)

**Mod. – Já sabia, estava-me a falar tanto de números que eu… hmmm… já estava aqui a desconfiar.**

**M. –** E tenho alguns clientes, uma dúzia, que são lares de idosos. E normalmente o trabalho de exterior passei eu a fazer, de há alguns anos a esta parte, e, portanto, lidava muito com eles. Não é… ia lá… era das coisas que me custava de ver, os velhotes na situação em que estavam… e vejo que estou a caminhar para lá também. Tudo o que a gente possa fazer para exercitar o cérebro, é útil… Agora pergunta-me se eu faço isso? tenho de dizer que não. De facto, não faço. Não consigo arranjar muito tempo para essas coisas. se calhar por falta de organização.

**Mod. – (informação sensível), vamos só interromper a gravação. Esta parte já está concluída.**

*Participant 2*

**Mod. – Ia dizer…**

**F. –** É para entrar no supermercado?

**Mod. – Pode ser. Experimente.**

**F. –** E agora…

**Mod. – O que é que vê?**

**F. –** Vejo um cesto, tenho que ir às compras. E agora terei que escolher as compras, não é? Tenho de pegar no cesto, será que ele vem? Não… não vem, pois não? A lista de compras… não trago lista de compras. Ainda não tem nenhuma lista de compras, pois não? Não tenho. Criar…. Ora, mas eu não tenho lista. Aqui diz para eu adicionar, mas não tenho lista de compras. Não deveria fazê-la primeiro? Criar lista de compras.

**Mod. – OK.**

**F. –** Lista de compras. Criar lista de compras. Eu agora ponho aqui… por exemplo, aqui… peixe, que é uma coisa que eu gosto muito. Aí, o que é que eu fiz… peixe. E, agora, ponho na lista. Só não percebo como é que eu vou pôr…

**Mod. – Diga.**

**F. –** Na lista, eu vou pondo. Agora…

**Mod. – O nosso supermercado, como ainda é novo, abriu há pouco tempo, ainda não temos todos os produtos. Escreveu carne.**

**F. –** Carne e peixe. Portanto, ainda posso pôr mais?

**Mod. – Ele aqui, ele abriu esta janela.**

**F. –** Abriu esta janela com carne picada.

**Mod. – Escrevendo carne, é o único item que temos neste momento. Se quisesse incluí-lo…**

**F. –** Se eu quisesse incluí-lo, punha aonde? Aqui? não. Aqui?

**Mod. – Clique só, nela. Ok…**

**Mod 2. – À esquerda temos…**

**F. –** Escreva o nome do produto. Escrevo tomate, não aparece adicionar à lista. Estão com a ideia do tomate, eu ponho o tomate. Não… está mal. Tomate para salada, tomate chucha, tomate rama, tomate cherry…. Pode ser para salada.

**Mod 2. – Aqui à direita, o que nós…**

**F. –** Carne picada, tomate… está aqui as compras que eu… agora, é para continuar?

**Mod. – Pode escrever mais produtos.**

**F. –** Pode ser fruta.

**Mod. 2 – Que tipo de fruta?**

**F. –** Tipo de fruta… maçã, por exemplo.

**Mod. – Ele aqui…**

**Mod. 2 – Tinha de ser com a cedilha, não sei como se faz aí.**

**F. –** O meu teclado aparece já. Este aqui não tem.

**Mod. – Podemos escolher outra fruta?**

**Mod. 2 – Tem ali mangas, pode ser?**

**F. –** Manga… manga… e agora…

**Mod. 2 – Mas manga, já lá estava.**

**F. –** Este produto já se encontra na lista. Pois, tem agora…

**Mod. – Vamos tentar bananas.**

**F. –** Bananas… saltei sempre qualquer coisa. Bananas, e agora…

**Mod. 2 – Para adicionar à lista, como é que podemos fazer?**

**F. –** Como adiciono à lista? Ainda não percebi.

**Mod. – Agora vai fechar o teclado, bastando para isso carregar no ecrã.**

**F. –** Tenho carne, manga, tomate e bananas. E agora…

**Mod. – Vamos supor que temos a nossa lista para hoje. E vamos querer ir às compras.**

**F. –** Ah, pois. Agora vamos às compras. Seguir…. Aparecem-me mais coisas.

**Mod. 2 – O que diz aí?**

**F. –** Lista de compras.

**Mod. 2 – Já tem a lista criada.**

**F. –** Selecionar?

**Mod. – Sim.**

**F. –** Adicionar… [impercetível] Então, tenho fruta. Vou ao supermercado, não é?

**Mod. – Ainda não consegue ir, correto? O que diz por baixo dos produtos?**

**F. –** Insira aqui o produto.

**Mod. – Por baixo da palavra produtos, diz.**

**F. –** Toque no produto e na secção para adicioná-la à mesma. Isto havia de aparecer… não é aqui? Aqui é insira produtos.

**Mod. – Aconteceu qualquer coisa.**

**F. –** Qualquer coisa…

**Mod. – Consegue dizer-me o que é que aconteceu? Deste lado temos os produtos da nossa lista de compras e aqui temos as secções do supermercado. Aqui diz toque no produto e na secção para adicioná-lo. Está a pedir para organizar os produtos nas várias secções. O** **tomate iria para aonde?**

**F. –** Hortícolas.

**Mod. – Experimente. Acha que aceitou?**

**F. –** Tomate acho que sim.

**Mod. – O que a plataforma tem que indica isso?**

**F. –** Está em verde. Clique na carne picada. Adicioná-la… carne picada vai para talho. E agora…

**Mod. – Inseriu?**

**F. –** Não…

**Mod. – Se quisesse ver se tem alguma coisa na secção do talho, como faria?**

**F. –** Se quisesse o quê?

**Mod. – Se quisesse ver se a carne picada ficou na secção do talho… exatamente.**

**F. –** Carne picada está. Está lá. E então agora são só as mangas. As mangas que é nas frutas…, mas não está verde.

**Mod. – Estão as duas verdes.**

**F. –** Ah, estão as duas verdes. Está bem. Tem as bananas e as mangas, o tomate e a carne, que era o que eu tinha comprado. Aqui a carne picada no talho, os legumes e as frutas. E agora quero… pôr isto aonde, no cesto.

**Mod. – Agora, isto é a nossa lista. Agora vamos ao supermercado. Para ir ao supermercado, como poderíamos ir?**

**F. –** Como poderíamos ir ao supermercado. Temos de escrever aqui supermercado. Coloque o produto na secção para adicioná-la a mesma [a entrevistada continua a ler o texto, murmurando]. E agora… o nível para jogar. Isto é um jogo. Vou para o mais fácil, para este.

**Mod. – Entrou para a secção de supermercado.**

**F. –** E agora clico aqui para… começar. Continuar. Não… ainda não fiz as compras. Não é? tenho de fazer as compras primeiro. Ponho aqui continuar… Não…. Finalizar não. Sair também não, ah, está aqui uma seta. Deixa lá ver uma seta. Chegou à nossa secção de legumes hortícolas. Procure a seta que aparece no ecrã para conhecer…. Aqui não está nada do que eu pedi. Aqui é para adicionar ao cesto. Posso adicionar…, mas não. E posso virar?

**Mod. – Pode, pode.**

**F. –** Se calhar é isso. Então, agora… ah, aqui não tem a carne. Tem. E aquilo que eu pedi. Tenho de clicar naquilo que eu pedi ou não. Não, porque eu tenho uma lista, não é?

**Mod. – Sim.**

**F. –** Bananas. Não tinha lá maçãs… tinha bananas. Tinha bananas e tinha o quê mais?

**Mod. 2 – Mangas.**

**F. –** Manga não tinha. Tinha manga?

**Mod. 2 – Íamos pôr a maçã e pusemos a manga.**

**F. –** Aí foi? Adicionar ao cesto.

**Mod. – Este som diz-lhe alguma coisa?**

**F. –** Está a dizer que está… que está no cesto. Agora é as bananas. Que ainda não estão lá. Ainda não tenho tudo.

**Mod. – Temos, então, de voltar para trás.**

**F. –** Temos de voltar às frutas que era… agora era carne e peixe. É só? Já não me lembro o que é que pus.

**Mod. 2 – Então se calhar vamos pagar e experimentamos outro nível.**

**F. –** Não tenho de ir buscar a carne e o peixe?

**Mod. – Vamos experimentar pagar.**

**Mod. 2 – E depois vamos fazer outro.**

**F. –** Aqui é pesar e aqui é pagar.

**Mod. 2 – A pagar…**

**Mod. – Então vamos… vamos fazer o…**

**F. –** Peguei neste, foi mais fácil. Vamos pagar.

**Mod. – Temos agora de começar de início, sem querer fechou.**

**F. –** Tenho de ir outra vez fazer as compras, não é?

**Mod. – Sim.**

**F. –** Que é… outra vez as frutas, quero ir buscar a manga.

**Mod. 2 – Ainda se lembra de onde estavam as mangas?**

**F. –** Pois… Quer dizer que já está.

**Mod. 2 – E agora as mangas…**

**F. –** Mas eu tinha-a aqui.

**Mod. – Experimente rodar a imagem. Isso…**

**F. –** Aqui é a manga.

**Mod. – Se calhar tem que se aproximar um bocadinho mais dela, usando…**

**Mod. 2 – Aí se calhar já dá.**

**F. –** Não, já não vai.

**Mod. – Temos que nos pôr ao contrário. Aqui um bocadinho… exato.**

**F. –** As bananas já estão. E está aqui a manga. E adicionar ao cesto. E agora pago?

**Mod. 2 – Sim. Agora o pagar…**

**F. –** É aqui.

**Mod. – Fazia sentido carregar na caixa e poder pagar? Ok. Mas ainda não…**

**F. –** Não está tudo.

**Mod. – As coisas ainda não estão dessa forma, tem de carregar num botão específico para conseguir pagar. Consegue identificar onde é que… exato. O que acha que…**

**F. –** Agora, próximo nível.

**Mod. – Sim.**

**F. –** E tenho de arranjar outro cesto, ou é o mesmo.

**Mod. – Não sei se percebi a sua pergunta.**

**F. –** Agora vou para outro nível, tinha pegado neste cesto, agora pego no mesmo ou noutro?

**Mod. – Terá que ser noutro, olhando para o ecrã… se quisesse. Exatamente.**

**F. –** Supermercado, tenho de fazer outra vez outra lista de compras ou começar? Deixe cá ver… aparece no ecrã e dirija-se à nossa secção de frutas. Mas nas frutas, acho que já tinha comprado. Chego à secção de frutas, uma dieta equilibrada, é a frutinha. E, agora… esta lista não tem nada a ver com a outra.

**Mod. – Exatamente.**

**Mod. 2 – Mas há uma forma de consultar a lista aí no ecrã, no cantinho.**

**F. –** Pois, mas eu agora não fiz outra lista. Posso comprar sem lista, também?

**Mod. – Experimente.**

**F. –** Posso ir buscar cenouras, por exemplo. Aí não, está aqui a seta. Pronto, agora está mais para cá. O tomate já foi, pode ser batatas.

**Mod. – Tem de carregar outra vez. Aí experimente selecionar um desses produtos.**

**F. –** Adicionar ao cesto. Este não se encontra na lista de compras, consulte a lista de compras para saber que produtos deve comprar.

**Mod. – Antes de fazer continuar, apresentamos aqui um símbolo. Consegue identificá-lo noutro sítio, no ecrã.**

**F. –** Qual símbolo?

**Mod. – Este aqui. Agora fecha a caixa de diálogo e para consultar a sua lista de compras, como faria?**

**F. –** Aqui, finalizar… tenho as mangas, meloas e tangerinas. E, agora, ponho em algum sítio?

**Mod. – Qual o objetivo que acha, agora…**

**F. –** Agora era ir pagar, ir à caixa?

**Mod. – Poderia ser. Mas ainda estamos na lista de compras.**

**F. –** Ainda estamos na lista, pois. Faltam mais.

**Mod. – Ainda temos de ir comprar.**

**F. –** Mais fruta.

**Mod. – Estas frutas. Exatamente.**

**Mod. 2 – Para comprar as frutas temos de ir procurá-las, como fizemos com a banana, com as bananas.**

**F. –** Era tangerinas.

**Mod. – Experimente novamente. É aí. Mas primeiro tem que…**

**F. –** Continuar.

**Mod. – Novamente, continuar. E, agora, sim. Carregue novamente.**

**F. –** Frutas e legumes, foi o que eu… e agora vamos para a caixa?

**Mod. – Aconteceu ali uma coisa.**

**F. –** Ficou vermelho.

**Mod. – À partida, isto quererá dizer que....**

**F.** – não está bem. Há aqui qualquer coisa que não está bem.

**Mod. – não está bem. Vamos ver as outras, experimente a de baixo para…. Ainda se lembra dos produtos que tem de comprar para a lista de hoje?**

**F. –** Para a lista de hoje faltava-me a carne e o peixe.

**Mod. – Mas estamos agora numa lista diferente.**

**F. –** Mas estamos numa lista diferente, pois é.

**Mod. – Se quisesse consultar a sua lista, como é que faria?**

**F. –** Aqui.

**Mod. – Exato.**

**F. –** Meloa e tangerina. É o que eu tenho de comprar?

**Mod. – Sim.**

**Mod. 2 – Sim.**

**F. –** Portanto…

**Mod. – Posso-lhe dar uma ajuda. O supermercado é novo, é a primeira vez que cá está. Se virarmos assim.**

**F. –** Manga, cesto, tangerina… cesto. E o que era mais? Meloa, tangerina…

**Mod. – Tente ir à sua lista de compras, para ver…**

**F. –** Falta meloa, pois. Meloa, que está aonde… está aqui. Adicionar ao cesto. Acho que já está.

**Mod. – Confirme.**

**F. –** As três.

**Mod. – Parece que já estão as três. Agora temos de…**

**F. –** Pagar.

**Mod. – Exatamente. A parte chata da coisa. então vamos lá.**

**F. –** Vamos lá. como vamos pagar.

**Mod. 2 – Primeiro temos de ir à caixa.**

**F. –** Temos de ir à caixa.

**Mod. – Ainda se lembra do caminho?**

**F. –** Er… aqui. Não, não é assim, pois não? Assim não chego lá. Ok…. Está aqui uma caixa…ali já é a saída. Não deveria ser aqui?

**Mod. – Poderia ser, faria algum sentido?**

**F. –** Pois, carregue no botão, pagar as suas compras. Ah, a pagar, tem lá… pois, é a dinheiro.

**Mod. – Passou com…**

**F. –** Com três.

**Mod. – Acha que esta pontuação é boa?**

**F. –** Para mim é. Nunca fiz isto, não está mal.

**Mod. – Obrigada. Olhando para aqui, para a pontuação e para as estrelas, teve uma boa pontuação?**

**F. –** Tive uma boa pontuação.

**Mod. – Vou agora fazer umas perguntas, depois se quiser podemos voltar à plataforma. de uma forma geral, o que acha da plataforma. que dificuldades é que sentiu?**

**F. –** Não tive assim muita dificuldade, uma vez que nunca tinha entrado neste supermercado. Achei que não era assim muito difícil.

**Mod. – Achou fácil andar pelo supermercado?**

**F. –** Sim.

**Mod. – As setas eram fáceis de utilizar?**

**F. –** Eram fáceis de utilizar.

**Mod. – E o que gostou mais?**

**F. –** Gostei do jogo todo, nunca tinha jogado este jogo. Achei engraçado, achei… claro que tenho um bocadinho de dificuldade na… quero dizer, na velocidade de encontrar as coisas. Sou um bocadinho lenta em encontrar as coisas. Mas não achei muita dificuldade. É mais o procurar as coisas, saber onde irei carregar, essas coisas todas.

**Mod. – Percebi que para a (informação sensível) foi muito fácil virar a imagem, rapidamente percebeu que dava para fazer, rodar a imagem. Quando fazia isso, alguma vez sentiu tonturas, náuseas, sentiu que tinha dificuldade em focar?**

**F. –** Não, não tive. Quando percebi que se eu rodasse iria ter a outro sítio, não me fez assim diferença.

**Mod. – Durante estes dois níveis que jogou, houve alguma altura que se tenha sentido** **perdida, sem saber qual era a tarefa que tinha de fazer?**

**F. –** Sim, algumas vezes senti. Quando queria pagar, como é que havia de pagar. Isso senti, senti alguma dificuldade. Mas depois de uma luzinha vossa, chegava lá com facilidade. Mas… houve algumas alturas que tive dificuldade em como chegar às coisas, chegar lá.

**Mod. – Mas só à caixa de pagamento ou se quisesse ir buscar um determinado produto, também tinha essa dificuldade em saber como havia de lá chegar?**

**F. –** Não, como tinha as setinhas, ia carregando nas setinhas e elas iam-me levando aonde eu queria ir. Não foi assim muito difícil.

**Mod. – Aquelas caixas de texto que aparecem inicialmente, para si aquilo faz-lhe muito sentido, as introduções de início ou acha que não valeria a pena ter esse texto e poderíamos passar logo à parte do supermercado?**

**F. –** Não, acho que para quem não sabe, faz sentido. Estarem lá, acho que sim.

**Mod. – A linguagem é clara?**

**F. –** É clara.

**Mod. – Sentiu que estava num supermercado real.**

**F. –** De verdade. Senti, tinha cá as coisas, senti-me num supermercado.

**Mod. – E, por exemplo, era um jogo que poderia utilizar em casa, nos seus tempos livres?**

**F. –** Sim, gostava. Jogava.

**Mod. – E sentia-se à vontade para utilizar em público. Ao invés de estar em casa, quando fosse beber um cafezinho, estar ali um bocadinho.**

**F. –** Se eu já estivesse dentro do assunto que comecei agora… se eu começasse a jogar o jogo, daqui a um tempo saberia… então em público, já seria mais fácil.

**Mod. – Era algo que poderia falar a uma amiga sua, familiar seu?**

**F. –** Sim. Tenho netos que jogam e que têm estas, gostam de jogar estas coisas. Se calhar isto também gostavam, especialmente a mais velhinha, que tem 11 anos, já joga jogos. O outro é mais pequenito.

**Mod. – Poderia ser uma atividade que pudessem fazer em conjunto?**

**F. –** Exatamente.

**Mod. – A senhora com a sua neta e passarem ali um bocadinho.**

**F. –** Sim.

**Mod. – E, outra coisa, esta plataforma poderia ajudar a fazer as suas compras na vida real de forma mais rápida?**

**F. –** Talvez eu viesse aqui e fosse… fosse ir vendo, como é que… como é que chegava às coisas.

**Mod. – Acha que a organização é semelhante à do supermercado onde costuma ir?**

**F. –** Sim.

**Mod. – E que outras atividades é que gostaria de poder fazer assim num *tablet* e fizessem sentido para o seu dia a dia, outras coisas que gostasse de poder fazer no *tablet*.**

**F. –** Não sei… não tenho assim… ideia, só vendo assim… uma lista de coisas que eu depois olhasse e visse, eu gostava de fazer isto. Assim de momento não me estou a lembrar, talvez cozinhar, fazer cozinhados ou outra tarefa qualquer em casa.

**Mod. – (informação sensível), tens alguma questão que queiras acrescentar?**

**Mod. 2 – Não.**

**Mod. – (informação sensível), resta-me agradecer mais uma vez da sua disponibilidade.**

*Participant 3*

**Mod. – Comece então a explorar a plataforma.**

**F. –** Por isso vou ao supermercado? Vamos lá ver o que nos aparece aqui. O que isto quer dizer?

**Mod. – Isto é o login. Esta parte eu posso fazer.**

**F. –** Deviam oferecer-me uma coisa destas.

**Mod. – Olhando para esta primeiro ecrã. O que é que…**

**F. –** Diz no supermercado, receitas, lista de compras. E depois é para a gente desenvolver. Irmos lá ver.

**Mod. – Experimente mexer no ecrã, ver o que acontece.**

**F. –** Fazer o que fazemos, não é?

**Mod. – Experimente. Exatamente…**

**F. –** Algumas pessoas já fazem isto muito bem. Eu é que não…

**Mod. – Vai começar agora.**

**F. –** Nunca faço lista.

**Mod. – Nunca faz lista de compras?**

**F. –** Chego lá e olho. Nunca faço… nunca faço lista de compras, acho que não vale a pena. Nunca utilizo essas coisas, chego lá e faço. Tiro e…

**Mod. – Então, vamos continuar… neste momento, o que está a pensar, o que está a ver?**

**F. –** Vou experimentar.

**Mod. – OK. Agora, o que está a ver?**

**F. –** Criar... A lista de compras para jogar. Sei lá, criar uma nova lista, talvez?

**Mod. – Experimente. Vamos ver o que acontece.**

**F. –** Posso escrever?

**Mod. – Claro que sim.**

**F. –** Vou meter aqui o tomate. Escrevo mesmo?

**Mod. – Está a ir bem.**

**F. –** Está?

**Mod. – Está.**

**F. –** Mas não apareceu aqui escrito.

**Mod. – Continue a explorar. Se quisesse, onde é que acha que vai escrever o produto?** **Experimente, por exemplo, via este retângulo.**

**F. –** Escrever-lhe?

**Mod. – Experimente tocar-lhe, para ver o que acontece.**

**F. –** Ah, agora já ponho. Pode ser um quilo, não é. Um quilo…

**Mod. – Fugiu, experimente outro produto.**

**F. –** Vou fazer como gosta à minha neta. E agora, carrego…

**Mod. – Agora, quer escrever massas. Mas se… tire só último S, para apagar, seria ali. Aqui vai-nos aparecer um conjunto de opções. O nosso supermercado ainda não tem tudo, está no início, teria de escolher uma dessas opções.**

**F. –** Teria de ser esta, que é o que ela gosta. Agora andamos para diante?

**Mod. – Sim. Neste momento, o que acha que já fez?**

**F. –** Já pedi tomate e a massa.

**Mod. – E sabe isso porque se lembra ou há alguma coisa no ecrã que a ajuda a perceber isso?**

**F. –** Creio que não…

**Mod. – Vê aqui os produtos, não é?**

**F. –** Sim.

**Mod. – OK, seguindo aqui. Temos a nossa lista de compras, temos os dois produtos que escolheu, a atividade seguinte será ir para o supermercado fazer estas compras, não é. Apesar de não utilizar lista de compras. Vamos imaginar que utiliza e agora quer ir para o supermercado que é uma outra parte da plataforma, tente explorar o ecrã, de forma a perceber se existe outra forma de ir ter a esse supermercado. Pode mexer, andar para cima, para baixo.**

**F. –** Isto é muito engraçado. Tenho de ir aqui, não é?

**Mod. – Carregue no botão seguinte. O que é que diz o balãozinho?**

**F. –** Dê um nome à sua lista. Ok…

**Mod. – Sim. identifica algum campo onde seja para dar o nome à lista?**

**F. –** Este.

**Mod. – Exatamente, não tenha medo, está a ir muito bem.**

**F. –** Supermercado?

**Mod. – Por exemplo, pode ser.**

**F. –** Este também, tinha de escrever. Tinha de começar a escrever mais.

**Mod. – Agora, para dizermos ao *tablet* o nome que queremos dar, temos de dar essa instrução, não é? E fazemos isso através deste certinho, não é? Isto já está, exato. Carregue novamente. E agora já temos uma lista, o nome da nossa lista, queremos ir para o supermercado. Consegue identificar no ecrã novamente, explorando, andando para cima e para baixo, o que poderia fazer ir até ao supermercado?**

**F. –** Queria estas… tinha de ir aqui. E aqui, não é?

**Mod. – Experimente ler a descrição.**

**F. –** Está aqui, tenho de carregar aqui.

**Mod. – Toque no produto e na secção para adicioná-lo. Mas primeiro tem de tocar no produto. O que aconteceu ao tocar no primeiro produto?**

**F. –** Ficou sublinhada.

**Mod. – O que acha que isto lhe diz?**

**F. –** Que não me vou esquecer.

**Mod. – E agora tem de colocá-lo na respetiva secção. Que seria qual, nesse caso?**

**F. –** Está cá já no coiso.

**Mod. – E vê mais alguma coisa que lhe diga que essa seleção que fez está correta?**

**F. –** Está, porque está nos legumes e hortícolas. Este carregamos aqui e vamos aqui.

**Mod. – Veja isso.**

**F. –** E já está.

**Mod. – O que fez agora, nesta etapa?**

**F. –** Já está na lista.

**Mod. – Pois. E agora resta-nos ir…**

**F. –** Para o supermercado. Carregamos aqui, não é?

**Mod. – Sim, no botão. E agora, o que vê?**

**F. –** Vejo lá tudo, pegar o cestinho.

**Mod. – Experimente. Ainda não temos essa opção, mas era muito interessante não é. A reação que tem é carregar no cesto das compras…**

**F. –** E depois vir aqui.

**Mod. – Isso, experimente, tocar, mexer.**

**F. –** Vir aqui. O que a gente encontra.

**Mod. – O que está a tentar fazer neste momento?**

**F. –** Ir às hortaliças.

**Mod. – Consegue, explorando o resto do ecrã. Não só a imagem, não só a fotografia, mas o resto do ecrã, alguma coisa que indique, que tem de fazer alguma coisa antes de ir para as hortaliças.**

**F. –** Então, é só o cesto. E depois… caminhar.

**Mod. – Então olhe, tem este botão do lado a dizer começar. Ok…**

**F. –** Carrego no cestinho, mas não vai lá. A gente vai aqui ao tomate. Disse para vires aqui…

**Mod. – Aí onde tinha o dedo, consegue identificar alguma coisa, além das hortaliças?**

**F. –** Está aqui a fruta.

**Mod. – Tem aqui esta seta. Experimente carregar na seta. Consegue ver a seta? Experimente tocar-lhe assim. Exatamente.**

**F. –** Ah…

**Mod. – O que aconteceu?**

**F. –** Já está em ponto grande. Dá vontade de chegar cá e tim tim tim.

**Mod. – E o que escolheu para a sua lista?**

**F. –** O tomate.

**Mod. – Experimente adicionar. O que aconteceu? O que este som lhe diz?**

**F. –** Que já está.

**Mod. – O que tinha de comprar mais?**

**F. –** Era a massa. Agora temos de ir, onde a gente vai buscar a massa. Temos de rodar…sabe uma coisa que tenho dificuldade, é nos supermercados grandes.

**Mod. – Isto não é uma grande superfície, mas já é um supermercado com alguma dimensão.**

**F. –** Mas os outros metem-me uma confusão terrível. Porque… gosto mais de ambientes mais pequenos. Aquilo é muito confuso, barulhento.

**Mod. – E vai sempre ao mesmo supermercado?**

**F. –** Mais ou menos.

**Mod. – Vou só mudar uma coisa. Vamos esquecer a nossa lista de compras e vamos passar a esta secção da plataforma. O que vê aí, o que está a ver nesse ecrã?**

**F. –** Estou a ver os cestos das compras.

**Mod. – E que outras coisas estão na imagem?**

**F. –** As estrelinhas…

**Mod. – Sim. E vê mais alguma coisa?**

**F. –** Não. Ah, vejo a… se é mais fácil.

**Mod. – A descrição em baixo. Se eu lhe pedisse para começar no primeiro nível, onde é que iria carregar?**

**F. –** Aqui.

**Mod. – Ok. Agora, o que estamos a ver?**

**F. –** Estamos a ver o supermercado.

**Mod. – E se quisermos começar neste primeiro nível… exatamente. Se o som estiver muito alto, diga-me.**

**F. –** Só me incomoda é os barulhos secos, pancadas secas. Porque… este ouvido…

**Mod. – Faz-lhe mais confusão, não é?**

**F. –** Agora qualquer barulho não. É os barulhos secos e muito barulho faz-me muita confusão. Isto a continuar…

**Mod. – Carregue novamente.**

**F. –** Este é dos mais pequenos. Os supermercados mais pequenos dão-me mais jeito do que os maiores. Mas vou… gosto de ver gente. Gosto de ver movimento, gosto de ver gente. E vou às vezes para duas ou três coisas, e chego lá sempre há mais.

**Mod. – Acabamos sempre por trazer mais coisas.**

**F. –** Sim, penso muito na família. Isto dá jeito a fulano e dá jeito a sicrano.

**Mod. – Uma mulher prevenida…**

**F. –** E gosto de ajudar. Gosto de ajudar. Nunca me preocupo muito com isso. tenho uma coisa que é, eu nunca… mas isto é um contrassenso, gosto de coisas, não… há pessoas que gostam muito de amealhar dinheiro. O contacto com o dinheiro é muito bom. Eu não. Contacto com coisas. O dinheiro não, não tenho prazer. E faz-me falta, quero ajudar muita gente ao mesmo tempo. E o dinheiro faz falta para ajudar, mas acabo por usar o cartão…

**Mod. – Por falar em cartão, vamos ver como poderia pagar as contas aqui. Ainda se lembra da instrução que foi dada no início? O que dizia?**

**F** – Já não me lembrava.

**Mod. – Vamos recapitular. Um balão dizia que este era um supermercado virtual e que para andar pelo supermercado tem de carregar nas setas verdes. Que vai encontrar… encontra, consegue identificar no ecrã onde é que está a seta?** **Exatamente.**

**F. –** Nós temos de continuar.

**Mod. – E o que é que dizia?**

**F. –** Agora queria as massas, onde é que elas estão. Temos de procurar a massinha. Faz de conta que já fez as compras. Agora vamos para a caixa. Onde é que elas andam…. Estão aqui. Estão aqui…

**Mod. – Exatamente. Mas primeiro… vamos carregar na seta, para andar, conhecer um bocadinho o supermercado. O toque é um pouco mais seco.**

**F. –** Mandei continuar, então… já estou noutra.

**Mod. – Neste momento, o que quer fazer, quer andar em frente ou quer ver…**

**F. –** Até tem os meninos, há de estar um a chorar. Quer alguma coisa.

**Mod. – O que está à procura, neste momento?**

**F. –** Agora queria as massas, que é para completar.

**Mod. – Se quisesse andar em frente, como faria?**

**F. –** Quer que faça…

**Mod. – Não, está a ir bem. Leu o que estava escrito? Naquele balão que apareceu há pouco, estava a dizer para carregar no centro do ecrã e agora deslizar, carregue, toque, e agora desligue-se. Até encontrar a seta junto à ilha das frutas. Exatamente… é isso mesmo. O que é que está a dizer nesse balão?**

**F. –** Carregue na… e descubra quanto custa o quilo de maçãs.

**Mod. – Se quisesse saber o preço dessas maçãs, o que faria? Ok. Portanto, carregando na tabuleta que está lá…**

**F. –** Já nos diz.

**Mod. – E o que diz mais? Ok. Apareceu uma mensagem, o que diz?**

**F. –** Para adicionar ao cesto.

**Mod. – Não, esta mensagem aqui.**

**F. –** Este produto não se encontra na lista de compras. Consulte a lista de compras para saber que produtos deve comprar.

**Mod. – Consegue ver algum símbolo no ecrã que lhe faça sugerir… que lhe sugira que é uma lista de compras? Experimente carregar naquele. Ok.**

**F. –** Ah, já está. É baratinho.

**Mod. – É acessível este supermercado. E o que está a ver neste momento.**

**F. –** Tem lá as frutas, mangas, bananas.

**Mod. – E isto o que lhe diz?**

**F. –** Diz o preço…

**Mod. – Não é a sua… não é a lista que tínhamos feito inicialmente, é uma nova lista.**

**F. –** É uma nova lista.

**Mod. – Vamos deixar a outra lista parada, em standby e vamos agora tratar desta. Tem quantos produtos para comprar?**

**F. –** São dois.

**Mod. – OK. Então vamos tentar comprar esses produtos, como faria? Carregue, não tenha medo. Quer fechar a lista de compras? Para fechar uma janela… exatamente.**

**F. –** E agora, podendo carregar aqui.

**Mod. – Um toque mais seco. E apareceu um novo balão, o que nos diz?**

**F. –** Vai encontrar mangas e bananas. Carregue na fruta e vamos carregar nas mangas. E aqui… não, é aqui. É… Já lá está.

**Mod. – Exato. E, agora, falta-lhe comprar mais uma coisa.**

**F. –** Tinha de ir lá buscar outra vez.

**Mod. – Tinha de ir aonde?**

**F. –** O que me esqueci, mangas e…

**Mod. – Não se preocupe com isso, é uma lista que não foi feita por si. A minha pergunta era outra, tinha um determinado conjunto de produtos na lista de compras, se quisesse voltar a ver esta lista de compras, como faria?**

**F. –** Tinha de vir carregar aqui. Agora falta-me… falta-me as bananas.

**Mod. – Porque acha que faltam as bananas e não as mangas?**

**F. –** Já está o tracinho.

**Mod. – Exatamente.**

**F. –** Esta já estava lá. Pronto.

**Mod. – O que esse balão diz?**

**F. –** Para terminar as compras de hoje, pagar. Vamos continuar e finalizar, não é?

**Mod. – Seria uma hipótese. Mas neste caso ainda estamos no meio do supermercado, temos de ir a uma zona especifica…**

**F. –** Pois. Temos de ir à caixa. Não é?

**Mod. – Exatamente. Consegue identificar onde é que a caixa está?**

**F. –** Está aqui.

**Mod. – E se quiser ir até ela…**

**F. –** Tenho de clicar… está aqui, já está aqui. Já está aqui.

**Mod. – Fazia sentido carregar na caixa e ela aparecer. Mas neste momento tinha de usar a seta. Ok? uma nova… um novo balão.**

**F. –** Está aqui…

**Mod. – Mas isto é uma caixa de diálogo, tem que se fechar primeiro essa caixa. Está? Então vamos andar um bocadinho mais em direção à caixa, tem de clicar. E agora que estamos na caixa, como fazemos para pagar. Qual era a instrução que nos deram lá atrás? Olhando para todo o *tablet* e não só a imagem de supermercado, há alguma coisa que lhe sugira uma possibilidade para poder pagar?**

**F. –** Aqui?

**Mod. – Onde? À lista de compras?**

**F. –** Não… aqui.

**Mod. – Mais alguma coisa? Então, pagar. E agora aparece?**

**F. –** Em multibanco e dinheiro.

**Mod. – O que acha que aconteceu? O que isto lhe diz?**

**F. –** Diz para eu… que já posso pagar?

**Mod. – Exato. Diz-lhe também que, parabéns…**

**F. –** Parabéns, concluiu o seu nível 1 com três estrelas.

**Mod. – Acha que três estrelas é bom, mau?**

**F. –** Suficiente.

**Mod. – Se eu agora, olhando para este ecrã, acharia que é suficiente?**

**F. –** Sim.

**Mod. – OK, está bem. Agora vamos passar à segunda parte, espero que tenha gostado desta primeira experiência com o *tablet.***

**F. –** Não, é giro.

**Mod. – Até às vezes é mais fácil do que no telemóvel.**

**F. –** Sim, até por causa… não sou capaz de conseguir… há tantos anos que ando no inglês, não sou capaz de… os ingleses para mim metem-me confusão porque é tudo ao contrário. É…

**Mod. – São as diferenças. Mas é normal. (informação sensível), diga-me qual é a sua impressão geral** **da plataforma, que dificuldades é que sentiu, o que gostou?**

**F. –** Eu… é falta de hábito, não é. De mexer, tenho sempre dificuldade nas novas tecnologias. Reconheço isso perfeitamente. Com medo de estragar.

**Mod. – Sim.**

**F. –** É isso, o meu receio é sempre esse.

**Mod. – E como acha que se poderia sentir mais segura?**

**F. –** Estou sempre a pensar que vou ter de ter confiança, vou ter que insistir. É sempre o medo de errar, eu não gosto de errar. Por isso é que me custa habituar-me às coisas. O telemóvel já consigo perfeitamente utilizar o telemóvel, posso não ter muito certo, mas por exemplo, já tenho a lista das consultas na agenda. A lista que tinha para vir aqui, já está na agenda.

**Mod. – E o que mudou para conseguir fazer isso?**

**F. –** Aprendi aqui utilizar a agenda. E como o telemóvel é meu, não tenho receio de errar. Pronto, aquilo saiu, não está bem, deixa-te estar, está lá. Mas estão as coisas marcadas, já consigo marcar na agenda aquilo que tenho de fazer.

**Mod. – E o facto de ser um objeto que seja só a (informação sensível) a mexer dá-lhe mais confiança?**

**F. –** Dá-me mais confiança. É meu. Se eu errar. Tenho de pedir a alguém que me dê a volta àquilo.

**Mod. – Mas sente que assim não prejudica ninguém.**

**F. –** Não. Não prejudico ninguém, é meu. Sempre tive esse, é essa falta de confiança que eu noto. Às vezes é assim, porque é que eu sou assim.

**Mod. – São as nossas vivências.**

**F. –** É, mas é, as vivências. Não ter confiança. Quando estou à vontade, então…

**Mod. – A coisa até corre melhor.**

**F. –** É, corre bem, é.

**Mod. – Diga-me outra coisa, pensando nesta plataforma especificamente, o que é que achou mais apelativo, mais bonito, por assim dizer, na plataforma? Se é que achou alguma coisa.**

**F. –** Acho que é muito útil, gostava mesmo. E sempre tive a ideia de comprar… e depois esqueço-me. Mas mesmo até para o inglês, tinha uma colega no inglês, ela já tinha o seu programa e o programa era muito giro, se ela errasse, o programa dizia-lhe que estava errado e dava-lhe a resposta certa. E eu comecei a pensar naquilo, tenho de comprar isto para mim. Mas como eu gosto de ler e de manusear os livros… isso dá-me prazer. E estas coisas passam-me depois ao lado. Porque eu gosto muito de ler. Se não gostasse de ler, tinha que arranjar um hobby diferente. Mas como gosto de ler, estou bem.

**Mod. – Estou a perceber. Então, esta experiência de ir ao supermercado através de um** ***tablet*. O que é que achou?**

**F. –** Era giro, mas como nunca tenho aquilo certo, tenho… chego lá, vejo e compro. Pronto, às vezes vou com a ideia fixa de que aquilo me faz falta. Uma ou duas vezes por mês, faço as compras em conjunto. E, depois, sou ainda muito diferente, porque vou ao talho… vou ao talho, não vou ao supermercado. Vou ao mercado, não vou ao supermercado comprar o peixe, e vou ao lugar das frutas e faço as compras do dia-a-dia.

**Mod. – Vai a sítios diferentes consoante…**

**F. –** Sim. O supermercado vou em conjunto, agora não vou ao supermercado porque preciso de hortaliça, não vou lá. Vou ao lugar onde…

**Mod. – Já conhece e sabe…**

**F. –** E porque vou todos os dias.

**Mod. – Vamos só olhar um bocadinho mais para a plataforma. Vamos andar um bocadinho nela. O que acha deste aspeto, acha que isto tem o aspeto de um supermercado normal, algo que poderia encontrar no seu dia a dia?**

**F. –** Sim, acho normal. Não é muito confuso, muito grande, faço perfeitamente isso. Sim, sim… isso para mim é fácil, porque é pequeno e é fácil de lidar.

**Mod. – E foi fácil para si andar pelo supermercado, perceber como fazia para ir de um sítio** **para o outro?**

**F. –** Sim, é fácil. Porque não é muito extenso, não está confuso, está tudo no seu devido lugar. Não é confuso.

**Mod. – Peço desculpa. E, por exemplo, estes balões que aparecem acha que… ajudam a perceber o que é para fazer, a linguagem é uma linguagem que é fácil de compreender. Ou isto é muito…**

**F. –** Sim, é fácil de compreender, sim senhor.

**Mod. – Se tivesse isto em casa, passava algum do seu tempo a mexer nisto?**

**F. –** Sim. Ia ter curiosidade de familiarizar-me com isso.

**Mod. – E qual seria o seu objetivo, explorar mais este ambiente? Seria só ficar mais confortável a mexer?**

**F. –** Mais confortável e ver as coisas que estavam com o preço mais…

**Mod. – Comparar preços?**

**F. –** E saber o que estava em promoção. E depois iria lá, isso aproveitava.

**Mod. – As imagens para si são naturais, não há aqui nada que…**

**F. –** Não, são ótimas.

**Mod. – Que seja mais difícil de perceber. Vamos aqui para um corredor que tenha mais produtos. Isto é natural, este ambiente?**

**F. –** Sim, é.

**Mod. – Aqui diria que estaria aonde, nesta secção?**

**F. –** Do leite.

**Mod. – Ao fazer, a (informação sensível), muitas vezes virava a imagem. Esta rotação causa-lhe tonturas… sentiu algum desconforto visual?**

**F. –** Não, está bom. Ainda não… esta coisa ainda não me faz confusão.

**Mod. – Não?**

**F. –** Não.

**Mod. – Diga-me uma coisa, era capaz ou sentia-se confortável para usar esta plataforma em público, por exemplo?**

**F. –** Sim.

**Mod. – Não se sentia constrangida?**

**F. –** Não.

**Mod. – Era capaz de a recomendar a um amigo, familiar? Em que situações é que faria sentido dizer a alguém que conhece, há esta plataforma, experimenta.**

**F. –** Tenho pouca gente a quem falar assim. Mas os meus filhos já fazem muito isso. A minha filha faz muito. São diferentes, tiveram outra educação, outra… sobretudo a minha filha, quer a filha, quer o filho, fazem muitas compras. A minha filha perde e gasta dinheiro nisto. Nas compras vai ver o que há para a menina vestir, se há… depois manda vir. Isso ela faz muito isso. A roupa da menina, procura os saldos, se quer ir… ela agora está muito numa de (informação sensível), e se vier alguma coisa com promoção, e faz falta à garota, ela manda vir. E o (informação sensível) também.

**Mod. – Faz as compras pelas marcas.**

**F. –** Eles fazem muito isso.

**Mod. – Sabem as marcas que gostam.**

**F. –** E vão pesquisar. Depois dizem. Eu podia estar mais ao pé deles, mas cada um está na sua casa. Acho sempre interessante, mas eu tenho outra vida.

**Mod. – Aqui esta plataforma, neste momento, isto é mais para treino, não dá para fazer compras. Não gasta dinheiro e nem vão bater à nossa porta com as nossas compras.**

**F. –** Pois, exato.

**Mod. – Acha que isso tira valor à plataforma ou, melhor, iria baixar o seu interesse em mexer na plataforma? O facto de não ser real e o treinar…**

**F. –** O treinar e a curiosidade em ver o que está em promoção. Está isto… não quero que passe. Neste sentido assim.

**Mod. – Ter um correspondente na vida real.**

**F. –** Sim.

**Mod. – Vou só mostrar mais uma coisa, é este separador das receitas. Vou querer que me diga a sua opinião, aqui conseguimos escolher uma receita, esta parte é atrativa, poder ver os ingredientes num creme de cenoura, teríamos os ingredientes deste lado.**

**F. –** Já sabia que não ia comer nada que não estivesse… está giro.

**Mod. – Não sei se percebi o que me disse. Qual era o…**

**F. –** Por exemplo, naquilo dos legumes, no creme… esse creme, ia saber o que está lá e só depois ia comprar.

**Mod. – Aqui o objetivo será termos o azeite, que é um dos ingredientes, colocá-lo na secção, aquilo que fez há pouco. A pimenta nas especiarias, o sal nesta secção, curgetes, cebolas, tudo na secção de legumes e hortícolas e batatas. A seguir íamos para o supermercado e aqui… se abrisse a lista de compras, já estava os ingredientes para esta receita. O que acha desta…**

**F. –** Está ótima. E já se vê o preço. Isto fica barato.

**Mod. – E, portanto, era importante ver o preço?**

**F. –** Sim.

**Mod. – E em termos das receitas, ainda temos poucochinhas, gostava de ter esta possibilidade de poder colocar as suas receitas aqui. Ou preferia ter acesso a receitas que não conhece? O que lhe fazia mais jeito? Mais sentido…**

**F. –** Não sei, porque lá em casa tenho de fazer as coisas… como a senhora gosta. É.

**Mod. – Mas para jogar, para se entreter, gostaria de conhecer novas receitas?**

**F. –** Sim, gostaria de conhecer, sim. Até saber como é que se fazia.

**Mod. – Ter mesmo as instruções. Um livro quase de culinária, é isso?**

**F. –** É giro.

**Mod. – (informação sensível), tenho a agradecer uma vez mais a sua participação. Tem alguma dúvida, ficou com alguma questão desta sessão?**

**F. –** Não, só me deu mais prazer comprar uma coisa destas.

**Mod. – Mais uma vez, muito obrigada.**

**F. –** Gostaria, sim, é muito útil. Estou sempre a dizer, vou começar a fazer isto. Isto é giro. Mas é… e agora custa-me mais, com a idade, vamos deixando outras coisas, e fazer mais o que fazíamos sempre. E não entrar nesta… pronto, nestas… [a entrevista termina].

*Participant 4*

**F. –** Vamos lá ver aqui…

**Mod. – Portanto, neste momento o que é que… o que é que fez?**

**F. –** Ir ao supermercado.

**Mod. – Ir ao supermercado.**

**F. –** Sim.

**Mod. – OK. e agora, neste ecrã…**

**F. –** Ora, neste ecrã… enviar um email? Não… não é. Espere aí…. Será que tenho que fazer, mandar o meu email?

**Mod. – Tem que fazer o seu registo, não é. Aqui não vai ser preciso fazer o seu registo, mas é exatamente isso. Vou pedir que escreva participante…7. Tem que ser tudo…**

**F. –** Pois, espere aí. Deve estar tudo. 7…

**Mod - @**

**F. –** Estava ali também o arroba, não era necessário vir para aqui.

**Mod. – Gmail.**

**F. –** Participante@gmail...

**Mod. – Hmm hmm**

**F. –** Ponto com.

**Mod. – Pronto, aqui, antes de passarmos para baixo. Aqui é um I.**

**F. –** É um I aqui.

**Mod. – Sim. E tire o espaço à frente do 7.**

**F. –** Ah, ok. Não era preciso.

**Mod. – Mais um. E agora, exatamente, o sete…**

**F. –** Aqui.

**Mod. – E falta um L a seguir ao I, no gmail. Agora, o ponto… e em baixo a password é participante7.**

**F. –** Aqui?

**Mod. – Sim.**

**F. –** Ah, não é para ter espaço…

**Mod. – Sem espaço e sete.**

**F. –** Ok.

**Mod. – Sem o ponto. É só retirar… apague só uma vez, ótimo.**

**F. –** Ora, aqui…

**Mod. – O que é que… vá-me dizendo aquilo que está a pensar, o que está a ver.**

**F. –** Temos aqui os carrinhos, vou para os carrinhos. Ok… ora bem, ora bem, bem, bem… primeiro se tenho que lhe dar um lanche mas bem, começo aqui pelas frutas. Espere aí… ah, vamos explorar, ah, o tal supermercado. OK. ora aqui, para aqui não há nada que me interesse, temos as frutas que já foi, aqui… espere aí…Hm… temos aqui as frutas. Agora aqui…

**Mod. – O que fez agora?**

**F. –** Era para continuar. Este supermercado foi criado com o objetivo… não, não… então vamos sair daqui.

**Mod. – Porque quer sair do ecrã onde está?**

**F. –** Porque eu quero-o levar para outras prateleiras. Não tem que sair? Tenho.… espere aí. Então vamos pôr… continuar, não? Espere… não vai, tenho que sair daqui. Carregar na seta… peço-lhe no ecrã para andar… ou sou eu que não estou a ver a seta.

**Mod. – Tem que sair deste balãozinho, daqui. Deixe-me só… Portanto, a sua ideia é ir para um sítio específico do supermercado? É isso que lhe faz sentido?**

**F. –** Sim.

**Mod. – Ok.**

**F. –** Aqui temos os carros e temos a fruta… o cesto… aqui, continuar… ah, está aqui uma seta, agora é que vi a seta.

**Mod. – Este, este balão não lhe diz nada?**

**F. –** Este aqui?

**Mod. – Este aqui, a caixa de texto.**

**F. –** Legumes, hortaliças, a nossa seleção de frutas. Continuar… aqui… continuar… agora… aqui já fui, ah… ok. Repare que no seu lado esquerdo vai encontrar frutas, do seu lado direito vai encontrar legumes. Ok, continuar… devíamos ter aqui uma seta… ah… vamos aqui para a fruta. Ora vamos para aqui, aqui…toque agora no centro do ecrã e ao mesmo tempo deslize com o seu dedo para a direita. Hmmm hmm estamos aqui, vamos para aqui. Vamos aqui, aqui, aqui… aqui e aqui. aqui… temos aqui papas, não vou utilizar papas, mas enfim… não quero papas, quero farinhas, papas, azeites… quero ir para as bebidas, vamos lá embora. Aqui já andei, aqui já andei, vamos à procura, vamos à procura, vamos à procura, frutas e hortaliças, ok, ok, ok. temos aqui uma secção de bolos, porque não… aqui… um chazinho também faz falta. Vamos aqui para os chás. Ora bem… aqui com os chás. Mas eu quero sair daqui, não me interessa andar.

**Mod. – Se quiser sair daí, como é que…**

**F. –** Aqui… agora… com o seu dedo carregue nas maçãs e descubra quanto custa o quilo de maçãs. Características… se eu não consigo, ok, vamos embora.

**Mod. – Leu a mensagem que apareceu?**

**F. –** Aqui… ok. Preço, ok. vamos adicionar ao cesto, ok.

**Mod. – Faça novamente o que fez há pouco. E apareceu essa.**

**F. –** Não vi, é a tal coisa que não vi. Este produto não se encontra na sua lista de compras, consulte a lista de compras para saber que produtos deve comprar.

**Mod. – Consegue identificar onde estaria a lista de compras, no ecrã?**

**F. –** Sim, as primeiras que eu fiz, estavam para aqui.

**Mod. – Não, a lista de compras. O balão dizia consulte a sua lista de compras…**

**F. –** Não…

**Mod. – Então, eu vou-lhe dar aqui uma ajuda. É este aqui…**

**F. –** Manga… ah e as bananas. Eu nem sequer fui aqui. Lista de compras...Mangas e bananas, ok. vamos para as mangas… aqui. Aqui… vamos lá para as mangas, onde é que elas andam… onde é que elas andam aqui. aqui não está, aqui também não está… não as vejo… temos aqui as frutas, deviam estar para aqui. não estão… Ah… no seu lado esquerdo vai encontrar as mangas. Do seu lado direito as bananas. Carregue primeiro numa fruta e depois na outra, adicione ao seu cesto. Do lado esquerdo, aqui, aqui…

**Mod. – O que é que este som…**

**F. –** Quer dizer que já foi para o cesto.

**Mod. – OK.**

**F. –** Então, agora vamos então sair daqui. Onde é que está aqui…

**Mod. – Está à procura de quê?**

**F. –** Mangas, quero ir para os chás… não… antes dos chás quero ir para os bolos, onde é que andam os bolos? Eu já os vi aqui em qualquer lado. aqui… e aqui.

**Mod. – OK, mas na sua lista…**

**F. –** Só apareceu a manga ainda e as bananas.

**Mod. – E já tem todos os produtos da sua lista? Se quisesse saber como é que… exato.**

**F. –** Ah, as frutas. Lista de compras…

**Mod. – Olhando… olhando para a lista de compras…**

**F. –** As mangas já estão.

**Mod. – Falta-lhe então…**

**F. –** Os bolos. Terei que escrever? Não…

**Mod. – Olhando para a lista…**

**F. –** As mangas já estão.

**Mod. – Sim. faltam então…**

**F. –** Os bolos.

**Mod. – As bananas.**

**F. –** Mas eu não quero bananas. Ah, mas tem de levar bananas? Ah, ok. Ah, só queria as mangas.

**Mod. – São umas compras forçadas estas [risos]**

**F. –** Adicionar ao cesto. Sou obrigada a levar o que eles querem? continuar… para terminar às compras de hoje, dirija-se à caixa e carregue no botão pagar. Ah, a caixa. A caixa será aqui? não?

**Mod. – Experimente.**

**F. –** não…

**Mod. – Isso é a sua lista de compras…**

**F. –** A caixa será aqui, salvo erro.

**Mod. – Nesses botões do lado faria-lhe sentido ter aí um botão que…**

**F. –** Finalizar, não?

**Mod. – Experimente.**

**F. –** Já tem todos os produtos da sua lista de compras, dirija-se agora para uma das caixas. Ok, continuar… caixa de pagamento, continuar. Não… deve estar para aqui… não, não é para aqui. Não…

**Mod. – Eu aqui posso-lhe dar uma ajuda, porque é a primeira vez que está neste supermercado, correto?**

**F. –** Ah pois. É esta seta… que eu não vi.. tenho dificuldade…

**Mod. – Tem dificuldade em vê-la.**

**F. –** Em vê-la. Ok…

**Mod. – Devia ser de uma cor mais…**

**F. –** Temos aqui só as caixas. Acho que é as caixas. Espere aí… aqui… e aqui. quando chegar à caixa de pagamento, carregue no botão pagar. Canto superior direito para pagar as suas compras… Aqui. a seguir escolha o modo de pagamento…

**Mod. – Agora fazia-lhe sentido carregar na caixa da imagem para pagar.**

**F. –** Sim

**Mod. – Ok.**

**F. –** Não vejo cá ninguém. Não… pagar … por favor escolha o modo de pagamento, dinheiro ou MB. MB… repetir nível… vamos para o próximo nível?

**Mod. – Vamos para o próximo nível. Este aqui, só voltando aqui atrás…**

**F. –** Já foi.

**Mod. – Exato.**

**F. –** Começar, vamos lá vê-lo. Carregue na seta que aparece no ecrã, dirija-se à nossa secção de frutas. Cá está a seta, que eu não a vejo. Continuar, aqui… esta é a secção de legumes… e hortaliças. Carregue na seta que aparece no ecrã para ir até lá. Cá está… continuar. Onde é que está a seta. A fruta e os legumes… onde é que estão…

**Mod. – Está à procura de...?**

**F. –** Dos legumes, eles pedem-me os legumes. Vou para aqui. Tomate e salada… ok… este produto não se encontra no super… manga, outra vez as mangas, meloa e tangerinas. Ora frutas, manga, meloa… e tangerina. Ok, manga… manga… aquela… aqui… ora, chegou à nossa secção de frutas [a entrevistada murmura o texto] Meloa… falta a meloa, meloa… manga… mas a meloa já foi, não está aqui… acho que isto é meloa.

**Mod. – Se quisesse ver melhor…**

**F. –** Não, não é aquilo que eu quero. É isto que eu quero, manga, meloa e os legumes… isto é a manga…adicionar ao cesto, não... isto não é, abacate, não me saiu abacate, é a manga, é as tangerinas… que devem andar por aqui, adicionar ao cesto… não é… as bananas… meloa, meloa… onde anda a meloa? É a manga que já está, eu quero a meloa… e as tangerinas. Estará para aqui alguma tangerina? Onde é que elas andarão? É aqui…

**Mod. – Carregue novamente na…**

**F. –** É meloa e tangerinas.

**Mod. – OK. e carregando aí…**

**F. –** Então… aqui não estou a ver. deixa lá ver se é aqui… laranjas, não é. por isso, não é…

**Mod. – Se quisesse sair daí, o que é que…**

**F. –** Sair daqui. Pretende sair desta página? Não… vamos lá ver… vamos lá sair daqui, desta página. Mentira, não era.

**Mod. – Saiu do nível, não foi?**

**F. –** Carregue na seta [a entrevistada murmura o texto] dirija-se à nossa secção de frutas. Continuar… [a entrevistada murmura o texto] onde é que está aqui a seta… a seta… bom… aqui. Aqui… chegou à nossa secção de frutas… aqui, aqui… onde é que anda para aqui a seta. Vamos lá ver aqui o que me vai sair, é a manga, novamente a meloa e as tangerinas. Ok. Tenho aqui a meloa… vá lá, aqui a meloa… está aqui. Não é não, não é aqui… manga…

**Mod. – Sim.**

**F. –** Tangerina será esta? Não é, não é. onde é que anda a tangerina… meloa, manga, a meloa… falta a tangerina. Será esta? Tangerinas, ok.

**Mod. – Portanto, fazia-lhe sentido ter aí um botãozinho para a caixa?**

**F. –** Para a caixa. Não… não..

**Mod. – Tem que ir até à caixa.**

**F. –** Pois, é isso que ando à procura.

**Mod. – Ah…**

**F. –** Será aqui? Não, vamos ver aqui. não é…

**Mod. – Posso dar-lhe uma ajuda?**

**F. –** Pode. É para aqui?

**Mod. – Pode seguir esta seta, agora vira para cá, agora um bocadinho mais…**

**F. –** Está a ver porque não gosto do tablet? É por isso… aqui, ah, temos aqui a caixa…

**Mod. – Agora segue em frente…. Usando a seta.**

**F. –** Não, não é aqui. frutas, legumes, caixa…

**Mod. – Agora carrega novamente na setinha…**

**F. –** Não… para pagar, continuar… para pagar as suas compras. Continuar…

**Mod. – Tem que carregar no botão pagar para… exato.**

**F. –** Multibanco…

**Mod. – Agora como falou que gostava de fazer a sua lista de compras, vou-lhe pedir que olhando para o ecrã, hã, tente perceber… hã, onde é que poderia fazer a sua lista de compras. Se há alguma coisa… na plataforma, olhando todo, para todo o ecrã. Por exemplo, aqui em cima… no menu… aqui nesta barra, nesta barra preta…**

**F. –** Lista de compras.

**Mod. – Hmm hmm.**

**F. –** Nova lista de compras. Encontrar… ora bem..

**Mod. – O que vai fazer agora?**

**F. –** Ora bem, frutas… frutas… pão… carnes…

**Mod. – Mas antes de continuar veja que está a escrever os produtos, mas está aqui numa secção que diz nome e à frente do nome diz que escreva o nome da lista de compras.**

**F. –** Sim, escreva o nome da lista de compras.

**Mod. – O nome, como se lhe desse um título…**

**F. –** Então, como é que…

**Mod. – Não é uma coisa muito usual?**

**F. –** Sim..

**Mod. – Dar um nome à lista de compras?**

**F. –** Não, eu não faço lista.

**Mod. – Por norma não usa lista…**

**F. –** Não.

**Mod. – Agora se quisesse escrever os produtos como estava a fazer, em que secção é que iria colocá-los?**

**F. –** Será aqui? frutas… não..

**Mod. – Na nossa plataforma, tem que ser um bocadinho mais especifica em relação àquilo** **que quer comprar. Por exemplo, se quer comprar frutas tem que dizer qual é a fruta que quer comprar.**

**F. –** Ai é?

**Mod. – É. experimente. Não temos todas as frutas, mas…**

**F. –** Ah, não têm. Ok…

**Mod. – Talvez uma mais simples.**

**F. –** Vamos lá ver aqui. vamos aqui… quero laranjas. Um quilo… não seria assim?

**Mod. – Veja.**

**F. –** Seria, acho que era… laranjas… laranjas, um quilo, ok. laranjas, já está aqui. Agora o que é que… espere aí… lista de compras após selecionar um produto eles aparecerão aqui na lista de compras. Laranjas…mau.... apaguei?

**Mod. – O que aconteceu?**

**F. –** Se apareceram as laranjas?

**Mod. – Carregou…**

**F. –** Após selecionar um produto, aparecerão a azul aqui… na lista de compras. Se quiser remover o produto da mesma, clique no botão vermelho. Ah, eu removi-o, ok, escreva o nome do produto.

**Mod. – Hmm hmm.**

**F. –** Por exemplo, escrevo tomate e toco no nome. Mau… espere aí. Tomate… tomate chucha, tomate salada, tomate… tomate rama… tomate cherry, tomate… espere aí. Tomate salada…

**Mod. – Desapareceu, carregue novamente.**

**F. –** Tomate, ok. tomate…

**Mod. – Se calhar tem que apagar a última letra e voltar a escrever para ele reconhecer que está… exato…**

**F. –** Tomate salada. Mau… aí então, mexe… pois. Tomate…

**Mod. – Ele apareceu ali do outro lado.**

**F. –** Tomate, tomate. Pronto, e agora…

**Mod. – Experimente agora escrever outro produto.**

**F. –** tomate… ora… tomate já ali está, tomate, alface… alface… alface… alface..

**Mod. – Se o quisesse selecionar…**

**F. –** Alface... não será aqui? Espere, está ali. Ok. Está tomate, esta a alface… ora mais, vamos lá... batatas… batata…. batata… batata vermelha, ok, deve estar para aqui. está. Agora vamos para o pão. Não levo mais nada de frutas.

**Mod. – O pão ainda não temos.**

**F. –** Aí não têm?

**Mod. – Ainda não.**

**F. –** Ainda não têm. Então vamos para os bolos. Os bolos fazem mal à saúde, também não quero.

**Mod. – Exato.**

**F. –** Vamos para a carne. Eu não sou amiga de bolos. Carne picada não, não gosto.

**Mod. – Aqui na secção do talho…**

**F. –** Não interessa que a gente goste ou não goste, é isso?

**Mod. – Não, não. Aqui pode selecionar o que quer. Mas vamos supor que queria… queria frango…**

**F. –** Aí já não vou…

**Mod. – Pode apagar e experimente. Às vezes é a forma …**

**F. –** Frango.

**Mod. – ... como nós escrevemos as coisas. Há também....**

**F. –** Frango inteiro, com miúdos. Eu vou-lhe ser muito sincera, também não compro frango.

**Mod. – Não? Novilho…**

**F. –** Eu compro frango é caseiro, mas agora não interessa, está o frango, está o frango… pronto. Vamos aqui para o frango.

**Mod. – Vamos supor que já tem a sua lista de compras, não vai querer comprar mais nada. Que passo é que daria a seguir? Aí vai voltar atrás…**

**F. –** Ah, ok…

**Mod. – Eu posso rapidamente pôr aqui os produtos… tomate…**

**F. –** Ah, é o tomate…

**Mod. – O tomate, a alface, não era… era o frango e era mais um…**

**F. –** A batata. Era a batata…

**Mod. – Agora já temos novamente aqui os produtos.**

**F. –** E agora temos de sair daqui. Aqui é que está… agora aqui é que está, será aqui? Aqui não…

**Mod. – Experimente.**

**F. –** Acha. É aqui? Acha? Não é…

**Mod. – Vamos continuar a explorar o ecrã, para cima, para baixo...**

**F. –** Seguir… dê o nome à sua…não

**Mod. – Ele aqui obriga-nos a dar o nome à lista, vamos por exemplo colocar o dia de hoje, 28… no título da lista. Podemos pôr 28/01.**

**F. –** 28 de… 28 de janeiro. Pronto. A seguir… ou será que também querem o ano?

**Mod. – Vamos experimentar o seguir.**

**F. –** Supermercado… vamos ao supermercado… legumes e hortaliças, ao talho… temos aqui a batata, a batata, alface, mentira, não quis alface. Foi tomate, batatas…

**Mod. – Por baixo dos produtos tem uma instrução.**

**F. –** Toque no produto e poderá adicioná-lo à mesma, caso erre, pode ordenar o produto dentro da secção até acertar. Atenção que perde pontos, no final clique finalizar.

**Mod. – Aquilo que ele está a dizer é para clicar no produto e a seguir na categoria a que ele pertence.**

**F. –** Legumes, a batata é legume. Acho que é.

**Mod. – Experimente.**

**F. –** Aqui?

**Mod. – Na categoria legumes.**

**F. –** Nos legumes e hortaliças… será que é legume a batata? É. batata é legume. É… batata… agora vamos para o frango. Ora… vamos para o tomate. Tomate é legume… hortícolas, batata… tomate será legume ou será? Vou para aqui… ok… vamos para o frango. Vamos para o talho. Ok… eu não pedi alface…

**Mod. – mas ela agora está cá.**

**F. –** Mas não pedi. Não…

**Mod. – Isto não era suposto, houve aqui qualquer coisa. Ok... espere aí. Que ele está aqui com umas dificuldades. Ok, vamos organizar…**

**F. –** Aqui…

**Mod. – Quantas categorias é que…**

**F. –** Foi batata, foi a batata, frango e o tomate.

**Mod. – E as categorias, as secções, quantas é que tem no ecrã?**

**F. –** Tenho duas só.

**Mod. – Duas só. Que é? São...**

**F. –** Frango, o talho.. e os legumes e as hortícolas.

**Mod. – OK.**

**F. –** Porque é que o frango não dá no talho?

**Mod. – Experimente ver se ele já lá não está, como é que faria, andando para cima no ecrã.**

**F. –** Ah, está aqui. Agora está aqui a alface, não pedi, mas está bem… está aqui… ok. Mas o frango devia estar no talho.

**Mod. – E acha que não está?**

**F. –** Está… então? Bem... está por baixo do talho.

**Mod. – Há mais alguma coisa…**

**F. –** Quanto a mim o frango devia estar dentro do talho.

**Mod. – E não em baixo?**

**F. –** E não em baixo, porquê?

**Mod. – Aqui a intenção…**

**F. –** Aqui está por baixo os legumes e as hortícolas.

**Mod. – Está por baixo da secção.**

**F. –** É por baixo, ok.

**Mod. – Mas fazia-lhe sentido tocar no talho e o frango ir logo…**

**F. –** Bem, há uma coisa… há frangos que não estão dentro da zona do talho. Estão ao lado.... Devia estar… eu quanto a mim, o frango…

**Mod - Devia estar aonde?**

**F. –** Aqui e não aqui.

**Mod. – Na barrinha cinzenta, é isso?**

**F. –** Sim.

**Mod. – OK, estou a perceber.**

**F. –** Como isto está… os legumes e as hortícolas, isto tem o seu sujeito, está por baixo, mas quanto a mim não, não deveria estar. Mas também compreendo que por vezes a secção do frango ou a secção das carnes está muito desviada do talho.

**Mod. – Hmm hmm**

**F. –** O talho conforme queiram interpretar. Quanto a mim o talho é onde estão os funcionários. E depois há uma secção ao lado, onde há muitas coisas. Talvez isso não tenha nada a ver com o talho. Pode ter, mas pode não ter.

**Mod. – É o talho ou os frigoríficos.**

**F. –** Isso mesmo. Talvez… aqui deve ser o frigorifico. Quanto a mim acho que é isso. O talho não tem nada a ver. Aqui, exatamente… como a secção de legumes e as hortícolas… er… o tomate e a alface, talvez aquela zona… porque há uma parte frigorifica onde há muitos legumes, que a gente tem de abrir as portas para tirar as coisas. E há uma outra parte que não tem. Está mais…

**Mod. – São as ilhas, não é.**

**F. –** Pode ser que seja este caso agora.

**Mod. – E já organizámos a lista toda?**

**F. –** Falta as batatas. Vamos para aqui novamente, porque...

**Mod. – E agora, o que é que faria?**

**F. –** Agora insira aqui os produtos. Alface, batata, tomate, o talho, já está. E vamos… toque no produto, na secção para adicioná-lo… no final clique em finalizar. Espere aí, onde está o finalizar.

**Mod. – Este botão finalizar transformou-se em supermercado. Ainda não alterámos isso. Agora supondo que queria fazer esta lista de compras, iria ao supermercado.**

**F. –** Vamos… sim.

**Mod. – Mas hoje vamos ficar por aqui, está bem?**

**F. –** Ah, não vamos para o supermercado?

**Mod. – Já não vamos para o supermercado…**

**F. –** Então esqueci-me das compras.

**Mod. – Já estamos quase no final da nossa sessão. Eu vou deixar isto aqui e agora vamos passar à segunda fase onde eu lhe vou fazer umas perguntinhas. Vou começar por lhe perguntar o que achou desta plataforma.**

**F. –** Esta plataforma para mim… eu para mim o dia a dia não dá.

**Mod. – porquê?**

**F. –** Porque eu sou uma pessoa que gosto de explorar o supermercado. Eu entro, dou uma grande viravolta… não tem uma grande lista de compras. É assim, vamos lá ver se eu sei explicar. Não tenho o hábito de fazer as compras do mês. Não tenho. Faz-me falta, vou, ontem fui, saí daqui e fui. Fui… comprei aquilo que tenho necessidade de comprar. Não faço lista de compras.

**Mod. – E aqui gostava de entrar no supermercado e poder…**

**F. –** Era isso que andava a fazer, correr o supermercado.

**Mod. – Isso fazia-lhe sentido assim que… porque nós estamos a prever isso, mas é depois da pessoa já estar habituada à plataforma, aí sim ela podia navegar à vontade no supermercado, mas para si faz-lhe sentido é que isto seja possível logo desde o início? Que** **dá para ter uma experiência mais real, é isso?**

**F. –** Sim, porque eu nunca vou com aquele instinto de chegar ali, olhe, vou só fazer isto. Não, porquê… sempre me habituei a ter uma despensa cheia. É verdade… a minha, você vai à minha casa, e diz assim, se houver fome, eu venho aqui. É o que a minha nora diz, se um dia houver fome, se acabarem os supermercados, eu venho logo aqui. E a minha filha vive sozinha… e… se lá for à casa dela, faz o sinal da cruz. Muita gente lá vai e pergunta-lhe, oh (informação sensível) tu vives sozinha ou tens cá alguém em casa, ela tem a despensa recheada, porque foi muito habituada comigo. Faz-lhe falta, vem buscar. Tem sempre stock. E eu também. Eu tenho sempre stock. Nunca peço nada a ninguém. Tenho um quilo de farinha, só tenho um quilo? E se tiver dois. Eu vejo lá pessoas com uma grande lista, eu não… nunca me habituei… se calhar…

**Mod. – E gostou de fazer a sua lista aqui? ou o que é que achou…**

**F. –** Eu gostei de fazer aqui a lista, mas embaralha, não estou habituada. Mas é giro. É um jogo.

**Mod. – Que dificuldades é que sentiu?**

**F. –** As dificuldades que senti foi andar à procura das coisas. Porque, para já não conheço a plataforma, não sei se, isto é, o (informação sensível) ou o (informação sensível). E, segundo, não estou muito habituada, lá está, a trabalhar com isto. Se fosse com o computador, se calhar ia lá mais depressa. Isto a mim não me diz nada, é o que lhe digo. E muito menos os dedos.

**Mod. – Gostava mais…**

**F. –** O rato.

**Mod. – Com o rato. Também há essa possibilidade.**

**F. –** Mas é giro. Digo-lhe já que isto é giro…

**Mod. – Sentiu que estava num supermercado real, que as coisas têm o aspeto…**

**F. –** Não, este supermercado virtual… é engraçado… porque… como hei-de explicar, isto dá-nos a atenção de andarmos a explorá-lo com o dedo, irmos à procura, vasculhar, para mim era bom estar aqui uma hora ou duas e coscuvilhar o supermercado todo com o dedo.

**Mod. – Gostava?**

**F. –** Gostava, como eu às vezes vejo mails que me mandam, de museus, andar com o dedo e exploro o museu todo. Isto é engraçado por isso, uma pessoa andar aqui com o dedo, explorar o supermercado todo e depois ter a noção de onde é que me vir de dirigir, às bebidas? As bebidas estão aqui, a carne está ali… porque é isso que eu faço. Quando vou a um supermercado, que não o conheço, nunca lá entrei, não vou diretamente às prateleiras, não sei onde é que elas estão. Temos que procurá-las. Isso é exatamente… quando a gente vai trabalhar para um hospital, primeiro o hospital é apresentado à gente. Senão depois perde-se lá dentro.

**Mod. – Exato... Conhecer a casa.**

**F. –** Primeiro apresentaram-me a casa quando entrei, depois de conhecer bem a casa, ainda andei dois ou três dias acompanhada. Porque a gente não aprende logo, quando se conhece a casa. Temos de andar acompanhada. E depois comecei a andar sozinha. Isto é exatamente a mesma, quando uma pessoa não conhece o supermercado, primeiro vamos ver, o talho é ali, o peixe aqui… e falta ir ao peixe. Que eu gosto de peixe.

**Mod. – Diga-me uma coisa e aqueles balõezinhos, as caixas de diálogo que aparecia, elas não a ajudaram a perceber o que é que…**

**F. –** Estas caixas que aparecem é a mesma quando a gente vai ao supermercado e vemos os letreiros. Quanto a mim, não. Devia estar os letreiros. Porque nem toda a gente conhece os sinais, os símbolos. Eu não conheço este símbolo, agora já sei qual é este, começar… este aqui acho que deve ser quando a pessoa vai correr quando rouba, não? [risos] O boneco roubou qualquer coisa e vai-se embora. Pronto, lá está, aqui devia estar caixas, entrada… saída… porque nem toda a gente… aliás… eu acho que… metade ou mais da população, ai era bom que fosse, era bom que fosse metade.

**Mod. – Não conhece esses símbolos?**

**F. –** Não, só quem conhece é quem está a trabalhar com isso.

**Mod. – Exatamente.**

**F. –** Dizem que a informática é bonita, a informática está-nos a ajudar, a informática vai dar cabo dos empregos a muita gente.

**Mod. – Há quem defenda essa perspetiva, é verdade. É verdade…**

**F. –** Eu vou-lhe dizer uma coisa, já sei que é doutora, já sei que é doutora, mas fujo dos médicos como o diabo foge da cruz. Porque trabalhei…

**Mod. – Eu sou doutora, mas não esse tipo de doutora, sou psicóloga, não sou médica.**

**F. –** Então ainda bem que não é, porque eu fujo.

**Mod. – Deixe-me fazer-lhe mais umas perguntas.**

**F. –** Faça, minha querida.

**Mod. – Aqui o mexer…**

**F. –** É giro.

**Mod. – Mas isto causa-lhe alguma confusão?**

**F. –** Não... confusão será a primeira vez. Agora se viesse cá, ao fim de um dia ou dois, já explorava mais com o dedito, não é?. Já entrava aqui para as garagens dos carros, já ia para as frutas… lá está, vê… a gente… é assim que nós…

**Mod. – Mas isso não lhe causa tonturas…**

**F. –** Não.

**Mod. – Alguma náusea…**

**F. –** Não…

**Mod. – É normal?**

**F. –** Sim.

**Mod. – É uma manipulação normal, está bem.**

**F. –** Não, é giro.

**Mod. – Há bocado estava-me a dizer que gostava de explorar a plataforma. Sentia-se à vontade para fazer isso em público. Estar… não sei, não sei se costuma ir ao café beber o seu cafezinho…**

**F. –** Não… não frequento cafés. É mau, não é?

**Mod. – Não, de forma alguma. São hábitos.**

**F. –** Não frequento cafés.

**Mod. – A pessoa ou tem ou não tem.**

**F. –** Não frequento cafés porque não os bebo. Porque já tive quase às portas da morte por causa do café. E fiquei… o organismo não aceita desde essa altura. Mas eu não tive a culpa daquilo que aconteceu. A culpa foi o hotel, mas não entramos agora por aí. Já lá vai há muitos anos e há muitos anos que não bebo. Se me vier o cheiro ao nariz, gosto do cheiro, mas se o meter na boca, fico nauseada. Fico… já fiz exames, já fiz tudo e a vesicula não aceita, derivado à carga exagerada que foi naquele dia. E a vesicula não aceita o café.

**Mod. – Vamos imaginar um cenário diferente, estar com o seu marido, numa das suas viagens. Tem ali um tempinho morto…**

**F. –** Vamos passear.

**Mod. – Vamos passear. Sentia-se à vontade para estar num... sentadinha…**

**F. –** Com uma coisa destas na mão…

**Mod. – Para explorar um bocadinho a ver o que é que…**

**F. –** Isso eu levo o meu …

**Mod. – O seu telemóvel.**

**F. –** ou levo o telemóvel ou levo o meu computador e é dentro do quarto do hotel. Quando saímos do hotel não é para explorar nada de mãos, é para andarmos… para vermos…

**Mod. – Para explorar o ambiente. Mas imagina que conhecia alguém que começava a ter algumas dificuldades em ir às compras. Acha que poderia ajudar a pessoa a ganhar algumas estratégias…**

**F. –** Sim, sim, sim, sim. Já tenho ajudado dentro dos supermercados.

**Mod. – Mas pensando na plataforma. A pessoa fazer em casa para…**

**F. –** Bem… eu talvez até pudesse ajudar e talvez ajudasse porque vou conhecendo o mundo da informática, pessoas que não conhecem, talvez fosse por isso. Não é que isto… sim… ajudava porque, consigo andar e consigo… e… esta não está muito explicito, devia haver letreiros, que não há. Pronto, mas aqui temos legumes, aqui temos coiso… falta a charcutaria, devia estar aqui mais alguma coisa, para irmos à charcutaria, por aí fora. Mas sim… não teria problemas em ajudar a pessoa. Mas não.. mas acho que isto não… se isto vai ser para o futuro… não.

**Mod. – É não.**

**F. –** Não, porque não vai encontrar muita gente jovem… gente jovem vem pouco às compras, é os velhos. E os jovens vão comer à casa dos velhos.

**Mod. – À casa dos pais.**

**F. –** Mas eu sei. Falo por mim própria. Já o fiz, noutros anos atrás, também gostava de ir comer a casa dos meus, agora vão comer à minha. Se fosse a minha filha, a minha filha ela explora isso tudo. Porque ela para comprar nunca vi.

**Mod. – Para concluir, quer deixar-nos alguma sugestão em relação à plataforma, o que seria engraçado fazer.**

**F. –** A sugestão que eu dou aqui é que devia haver letreiros. Mais para pessoas que não sabem ler. Por exemplo, não se lembrem de pôr letreiros em inglês, estou cozida com F grande. não sei inglês, já tenho que procurar, não acho correto. Quando vou para Espanha, não se esforçam para perceber a mim. Eu tenho de ir ao encontro deles e é se quiser alguma coisa. Porque é que nós somos obrigados a falar inglês para os que cá vêm. Porque é que eles não se esforçam.

**Mod. – Estou a perceber. Letreiros e tudo em português…**

**F. –** Acho que sim. E produtos portugueses, porque não.

**Mod. – Na verdade nós temos… vou-lhe mostrar aqui rapidamente… isto é um supermercado português. E é tudo… só para ficar com uma ideia.**

**F. –** 35 euros para fazer compras. O que é que eu comprava com 35 euros?

**Mod. – Acha que é pouco?**

**F. –** É. bem, eu comprava, o essencial para tapar a fome. Comprava-se pão… quê, umas frutazinhas, uns legumes e um peixito e....

**Mod. – Está a ver, isto tem os mesmos… entrando aqui… tem os mesmos produtos que…**

**F. –** Temos aqui… os detergentes, temos as setas… não é.

**Mod. – Dona (informação sensível) vou terminar a sessão de hoje. Quero agradecer-lhe a sua participação. Ficou com alguma dúvida?**

**F. –** A dúvida que fiquei é que isto não é explicito. Mais… não é explícito para a era em questão. Mas daqui a 15, nem tanto, daqui a meia dúzia de anos é capaz de ser já muito útil.

**Mod. – Acha que atualmente isto ainda não… porquê?**

**F. –** Porque, isto é, explicito nos meios grandes. Mas se a doutora for com os meios pequenos, nem olhou para aí. Porque eu frequento muito meios pequenos.

**Mod. – Quando diz meios pequenos…**

**F. –** Mais os interiores. Isto é bom é aqui neste meios grandes. E mesmo assim há muita gente que não é.

**Mod. – Sente que alguém que viva nas grandes cidades até se pode relacionar com isto. Quem viver para o interior, acha que é muito diferente…**

**F. –** Toda a minha família não entra.

**Mod. – Estão mais habituados em fazer as compras em que tipo de…**

**F. –** Verem, comprarem…

**Mod. – Mas vão a que sítios?**

**F. –** Ali naquela zona (informação sensível). Até mesmo… temos ali (informação sensível), que já tem umas grandes áreas, temos o (informação sensível), mesmo o (informação sensível) eles vão. É como eu digo, eles vão, exploram primeiro… veem e tal. Mas se houvesse coisas com nomes esquisitos, eles não sabem.

**Mod. – Tudo… tinha de ser tudo muito objetivo com nomes em português, os produtos que as pessoas estão habituadas…**

**F. –** Mas tem que ser. Nós não somos obrigados a comprar nada com nome estrangeiro, gostamos daquilo que é nosso.

**Mod. – Sem dúvida, sem dúvida**

**F. –** É o que eu digo. Eu exploro muito a Espanha. E se eu não compreender, não vale a pena procurar. Tem que ser eu… por umas coisas tiro outras, sim, sim, é verdade. Eles não se esforçam absolutamente nada. Nada. São… não têm nada a ver com o português.

**Mod. – É nosso, esta questão de nos relacionarmos, é muito portuguesa.**

**F. –** Eu quando lá vou, também vou a centros grandes, quando lá vou passeio muito, olhe, este ano….

**Mod. – Deixe-me terminar aqui a… a gravação e continuamos depois. Dona (informação sensível), muito obrigada pela sua participação.**

**F. –** De nada, de nada.

*Participant 5*

**Mod. – De facto… a (informação sensível) é autora destes dados. Não vejo qual seria a utilidade, mas se se sentir mais à vontade.**

**F. –** Isto também demora o quê? Meia-hora, não é?

**Mod. – Então, quando quiser, pode começar. Esteja à vontade, pode explorar.**

**F. –** Isto é só para eu ir brincando aqui?

**Mod. – Exatamente. E fazer o tal pensamento em voz alta, o que é que as coisas lhe sugerem, se tem dúvidas, se não tem, para onde a sua atenção é captada.**

**F. –** Vai para aqui… ah, isto era o outro.

**Mod. – Eu posso fechar esta plataforma.**

**F. –** Isto é mais ou menos o que estava ali…receitas não, lista de compras, devia começar pela lista de compras, não é. Descobri há pouco tempo…

**Mod. – É o que lhe faz sentido?**

**F. –** Sim, porque eu quando faço as compras são coisas pesadas. Quando vou assim… estou a pensar compras online, se for uma coisa leve não é preciso lista. Mas quer que eu vá carregando, é?

**Mod. – Sim, que vá explorando. Está a tentar entrar no supermercado. Aqui vou-lhe pedir que coloque participante 12. @gmail…. Com o espaço, tem que…**

**F. –** Onde está a setinha? Está aqui.

**Mod. – E a password é “participante12”**

**F. –** Onde está a password? Está aqui. Outra vez a mesma coisa?

**Mod. – Sim, “participante12”, só.**

**F. –** Login?

**Mod. – Sim.**

**F. –** Agora posso explorar?

**Mod. – Exatamente.**

**F. –** Ah, isto é um jogo. Tanta coisa…

**Mod. – Não vai ter de fazer tudo.**

**F. –** Eu não costumo jogar, por isso não faço ideia o que é que sai daqui.

**Mod. – Neste momento, o que está a ver?**

**F. –** Os vários níveis. Isto é fácil, difícil? É o tal jogo. Eu estou focada no ir às compras e o objetivo não é esse.

**Mod. – Peço desculpa, vou pedir que comece no primeiro…**

**F. –** Ah… isto é engraçado. Já agora… está bem. Também aumenta. E agora… agora é começar.

**Mod. – Exatamente.**

**F. –** Só ver aqui, se consigo descobrir onde é que é isto. Ah…seta. Ah… está aqui. Este é para eu, sim… Ah, está aqui. Pronto… agora… já me esqueci o que é que estava lá escrito. Pronto, já vi tudo, é só isto?

**Mod. – Vou-lhe pedir que clique na seta.**

**F. –** No lado esquerdo vai encontrar fruta, no lado direita vai encontrar legumes. Está bem. Frutas, legumes, e agora…

**Mod. – Portanto, está a utilizar a seta que aparece no ecrã para andar para a esquerda e para a direita. É isso que lhe sugere a seta?**

**F. –** Ou para a frente e para trás.

**Mod. – Se quisesse andar para a frente, como faria?**

**F. –** Para ali. Para cima.

**Mod. – Iria deslizar o dedo. Vou-lhe pedir que clique na seta…**

**F. –** Toque aqui, vou para a direita. Tenho de encontrar as frutas. Estão ali.

**Mod. – Agora, se quisesse andar em frente, iria clicar.**

**F. –** É maças Fuji… não sei quais são as frutas. Devem ser estas. Características, peso… adicionar ao cesto. Mas eu não fiz lista nenhuma!

**Mod. – Olhando para este balão, há um símbolo. Consegue identificar esse símbolo em outra parte do ecrã ou não é intuitivo onde é que poderá… vou-lhe pedir que feche o balão, esse balão de diálogo.**

**F. –** Para trás?

**Mod. – Não, tem razão. Faz continuar.**

**F. –** E agora vou ali?

**Mod. – Experimente.**

**F. –** Tenho de fazer a lista, não é. Ah, eu gosto de bananas. Sou preguiçosa. Bananas é o que eu mais… ah, tenho de escrever? Não entendo isto… só há estas duas frutas?

**Mod. – Não. Olhando para o ecrã, o que é que vê?**

**F. –** As tais maçãs.

**Mod. – E no título dessa janela?**

**F. –** Ah, lista de compras. Está bem. Tinha de escrever algures. Onde é que isto se escreve?

**Mod. – Só tentando… traduzir um bocadinho o que está a acontecer, para ficar registado em áudio, ao abrir a lista de compras, faria sentido poder escrever a sua própria lista de compras?**

**F. –** Sim, a não ser que tivesse lá… o que está aqui, o que poderia fazer?

**Mod. – A intenção deste balão, desta janela, é já lhe dar uma lista de compras. Ao invés de fazer, teria uma lista de compras especifica para fazer. E, neste caso, seria comprar mangas e bananas.**

**F. –** Mas como eu ativo isto?

**Mod. – Vou pedir que saia, feche a lista de compras. Neste momento teríamos de ir à procura da lista.... das bananas ou das mangas. Vou-lhe pedir que clique na seta.**

**F. –** Ah, tenho de procurar mesmo. Então… lado esquerdo mangas, do lado direito bananas. Pronto, bananas. Ah, tem de ser com a seta?

**Mod. – Não, não. Estava a fazer bem.**

**F. –** Características, meio-quilo mais ou menos. Agora são mangas… deve ser isto aqui, meio disfarçada. Meio quilo de mangas é muito. Se eu quiser menos? Ah, isto é, uma. Já me aconteceu, pacotes de leite…ao invés de pedir um grande, pedi um pequenino. Isto é se eu quisesse adicionar ao cesto, adicionava uma manga?

**Mod. – Exato.**

**F. –** Há bocado só adicionei uma banana?

**Mod. – A pergunta que coloca faz todo o sentido. Neste momento a imagem é exemplificativa do produto que estamos a referir.**

**F. –** E a quantidade?

**Mod. – A quantidade será o meio-quilo conforme as bananas… já não sei qual era o peso** **que lá estava.**

**F. –** Mas meio-quilo para uma manga é um bocado. Para terminar as compras… dirija-se à caixa para pagar. ok. Ah, agora tenho de ir à caixa. Onde é a caixa? Acho que é ali… não é?

**Mod. – Sim, sim, é.**

**F. –** Agora clico aqui, como isto funciona?

**Mod. – Tem de clicar na seta, como estava a fazer.**

**F. –** Para aqui, isto é, com robôs, não tem pessoas. Quando chegar à caixa, carregue no botão pagar. Já estou na caixa. Acho que.... Três estrelas não é muito. Tenho de fazer isto tudo?

**Mod. – Não, vou-lhe pedir que faça o nível dois. E depois passamos para uma coisa** **diferente.**

**F. –** Ah, começar…. Pois, eles bem querem que eu coma fruta. Onde está… ah, está aqui pois, eu sei…fruta, onde está fruta…eu devia ter decorado isto, era?

**Mod. – Fazia-lhe sentido que pudesse consultar a última instrução, o último balão que leu. Ter um botão onde clicasse e aparecesse a instrução. É isso?**

**F. –** Ah, não, achei as frutas, mas foi assim um bocado sem pensar. Do seu lado direito… Outra vez. Ah, agora já tenho… ah, são diferentes, está bem. Já me esqueci de como é que era.

**Mod. – Esqueceu-se de…?**

**F. –** Há as frutas… e agora? Aqui o que eu fazia, já me esqueci…

**Mod. – Na lista de compras. Ok?**

**F. –** Ponho finalizar.

**Mod. – Neste momento, o que está a fazer?**

**F. –** Não sei, já me esqueci o que estava escrito. É asneira… pois, vou começar outra vez. Onde estavam as frutas? Ah, estão aqui. Pronto. OK, agora a partir daqui é que não me lembro do que fiz.

**Mod. – A partir do momento em que abre a lista de compras. Numa situação normal, tendo a sua lista de compras, iria então adicionar. E lembra-se como adiciona produtos ao seu cesto de compras, aqui na plataforma? Nesta? Consegue identificar as tangerinas?**

**F. –** Estão aqui…

**Mod. – Não são bem essas. Há outro sítio no ecrã…**

**F. –** Então são estas. Ah, é assim, está bem.

**Mod. – Como faz então para selecionar?**

**F. –** Tenho de ir lá à fruta, clicar, e depois aparece…

**Mod. – Ok.**

**F. –** Aparece o preço. E agora… ah, faz de conta que sim. Ah, agora é ir lá. Juntar aí…

**Mod. – Neste momento está a tentar fazer o quê?**

**F. –** Encontrar a seta. Toque no centro do ecrã.

**Mod. – Toque e arraste. Mais um bocadinho.**

**F. –** Ah, está bem. E agora consulte o… faltam, pois faltam, eram três. Agora tenho de ir fazer isto… Meloa e manga. Ah, isto tem… está bem. Ah, isto tem uma seta de um lado e outra do outro.

**Mod. – O que isso lhe sugere?**

**F. –** Dois caminhos.

**Mod. – OK.**

**F. –** Está aqui… ou não...

**Mod. – Está a ampliar a imagem, para conseguir ver melhor.**

**F. –** Sim, entretanto já me esqueci outra vez da outra. Porque é que isto não… porquê manga outra vez, as mangas estão ali. Eu já me esqueci outra vez o que é para fazer. Ah… é aqui? Não…

**Mod. – Percebeu o que aconteceu?**

**F. –** Fui para pagar, mas não comprei tudo.

**Mod. – Exato, foi em frente. Vou-lhe dar só uma ajuda…**

**F. –** Tinha de ir para ali. Mas porque é que isto não aparece? Como apareceu há bocado.

**Mod. – Pode ser da perspetiva onde está. Que não tenha acesso... Como está num corredor e as meloas estão noutro corredor. Embora eles apareçam juntos…, mas então fazia-lhe sentido que desta** **perspetiva fosse possível selecionar as meloas, não é? Visto que estão relativamente próximas.**

**F. –** Aqui na plataforma sim. Se estivesse lá, se calhar não chegava lá.

**Mod. – Deixe-me só ver se conseguimos andar para trás. Porque isto tem a ver com… com a distância.**

**F. –** Do corredor, não é.

**Mod. – Exatamente. Experimente agora. Este som é… diz-lhe alguma coisa, é sugestivo de alguma coisa?**

**F. –** Completei a lista, não é? Agora vou à caixa. Estou a partir do princípio de que isto é igual. Não é. Onde é que está a caixa?

**Mod. – A caixa está agora nas suas costas, por assim dizer.**

**F. –** Ah, está nas minhas costas. Está ali. Agora tenho de ir para lá…. Ou não… ah está aqui.

**Mod. – Faz sentido chegar à caixa?**

**F. –** Eu acho que foi esta. Não sei… Não foi?

**Mod. – Conseguiu perceber, que associado a essa, ao selecionar essa resposta, ouve um som? Não deu?**

**F. –** Ah, agora estão a dizer que foi errado.

**Mod. – Apareceu vermelho.**

**F. –** Entrada, eu devo ter entrado. Isto aqui está certo, pagar… Agora, continuo?

**Mod. – Sim. Não vai ter de fazer os níveis todos, mas pensando no que estava a dizer, na lista de compras, há possibilidade de fazer uma lista de compras nesta plataforma. Olhando para ela, consegue perceber onde iria fazer a lista de compras no ecrã?**

**F. –** No início tinha lá a lista.

**Mod. – E agora neste ecrã?**

**F. –** Está aqui. Tenho de escrever? Escreva o nome da lista de compras, pois é, isto também aparece lá. Compras do mês, dias da semana…

**Mod. – Qualquer coisa, pode só pôr…**

**F. –** Semana, nome de produtos. Escreva o nome do produto Por exemplo… b, banana. E agora? Já está…

**Mod. – Escreveu só…**

**F. –** Adicione pelo menos um produto. Tenho de escrever tudo. Oh…

**Mod. – Escreveu bananas, não é?**

**F. –** Está aqui. Não sei se foi para lá ou não foi. Após… ele aparecerá. Ok, apareceu. E agora, onde está o seguinte. Frutas… este aqui não me dá nada. Isto já está, então… ah, este é o supermercado.

**Mod. – Ele aqui está a pedir-lhe, como só escreveu um produto, só aparece um produto. Se na etapa anterior tivesse escrito vários, iriam aparecer os vários produtos. E aqui está a pedir que a cada produto associar uma categoria. Como só escolheu um produto, só aparece uma categoria. Vou pedir que carregue nas bananas e as inclua na categoria das frutas.**

**F. –** E agora continuo, tenho de escrever mais?

**Mod. – Nesta fase já não consegue, mas seria interessante incluir nesta…**

**F. –** Porque as bananas são muito pesadas.

**Mod. – Estou a perceber. Se fosse numa situação, vamos imaginar que queria fazer uma lista maior.**

**F. –** Iria fazer pela internet.

**Mod. – Mas faria sentido nesta fase ter ali um campo que pudesse adicionar mais produtos,** **que se pudesse ter esquecido?**

**F. –** Sim.

**Mod. – Pronto, já fez a lista de compras, desta vez é só bananas. Se agora quiséssemos treinar a compra, neste caso de bananas, como é que faria?**

**F. –** Bananas? Já lá está. Já me esqueci como é que é isto. Começar outra vez…

**Mod. – Exatamente.**

**F. –** Tenho de ir lá? já tenho lá finalizado. Já selecionou? Já… ok, onde está a caixa.

**Mod. – Já selecionou todos os produtos?**

**F. –** Continua a ser só bananas.

**Mod. – Mas ainda assim, nesta fase, já pôs as bananas no seu cesto de compras ou ainda vai colocá-las?**

**F. –** Ela estão ali. Ah, isto é só a lista. Como isto vai para o cesto? Tenho de as descobrir, não é?

**Mod. – Aqui posso dar uma ajuda, é seguir em frente. Exato… agora aqui, vou-lhe pedir que colocando o seu dedo no ecrã, arraste para a direita. Esta instrução é sempre mais complicada. Utilize esta seta, que aparece aqui.**

**F. –** Para andar para a frente?

**Mod. – Sim. Para andar para a frente tem de clicar. Exato. E as bananas vão estar nesse corredor.**

**F. –** Estão ali, não é? Agora tenho de carregar aqui. Ah…continua, finalizar… já estão lá. Ah, a caixa. Isto aqui não se vê as caixas, quais estão a funcionar, quais não estão.

**Mod. – Era interessante? Uma categoria que poderíamos interessar?**

**F. –** Não está cá pessoas. inteligência artificial…

**Mod. – OK. E então… Desta experiência até agora, o que é que achou. Qual é a sua…?**

**F. –** Para ser franca, não gostei muito. Para já não tenho *tablet,* não sei se é algum entrave, entre aspas. Estou sempre a pensar naquilo que não devo. Se eventualmente isto vai ajudar as pessoas a desenvolverem capacidades que tenham perdido, tudo bem. De memória, onde estava e onde não estava.

**Mod. – Da sua experiência a utilizar a plataforma, quais foram as principais dificuldades que sentiu?**

**F. –** Ah, esquecia-me sempre o que tinha de fazer. Eu gosto mais de utilizar as outras que eu faço, chego lá, escrevo as coisas, aparece-me ali. Não me aparece o supermercado, mas uma lista com o que eu quero comprar. Acho isso bem mais fácil. Embora para treinar outras capacidades, talvez não seja o mais correto.

**Mod. – E do ambiente do supermercado que vimos, achou que era realista?**

**F. –** Não, não tem ninguém. Há umas criancinhas assim ao longe. Mas não tem pessoas, as caixas não têm pessoas.

**Mod. – Achou estranho esse…**

**F. –** Sim, um jogo. Até porque só se ouve algum barulho, de crianças, de pessoas não. Agora ver, ver… se calhar ia baralhar aquilo que as pessoas têm de fazer e procurar. Mas achei muito artificial, parece mais um frigorifico gigante do que um supermercado.

**Mod. – Estou a perceber. E a nível de navegação, do andar no supermercado, achou fácil perceber como se andava no supermercado, ou foi confuso perceber como fazia para andar para a frente, ou se só quisesse virar um bocadinho?**

**F. –** Confuso, confuso não direi. Mas tinha de treinar mais. Não é assim logo à primeira.

**Mod. – Sentiu uma dificuldade, não foi uma dificuldade, mas uma estranheza em não poder selecionar a quantidade dos produtos?**

**F. –** Sim, também, exatamente.

**Mod. – E esta distinção que fazemos aqui, entre níveis de jogo e fazer a lista de compras. Isto faz sentido ou fazia mais sentido… começarmos por fazer uma lista de compras, em que a pessoa escolhe o que quer comprar e depois, sim, ir para o supermercado.**

**F. –** Sim, acho que faz mais sentido.

**Mod. – Seria mais realista, mais aproximado do que é uma compra no dia a dia?**

**F. –** Sim, a pessoa perde-se. Anda para trás e para a frente.

**Mod. – Houve algum momento em que se sentia perdida, não sabia para que lado é que havia de ir?**

**F. –** Talvez não soubesse em alguma situação ou outra, mas também andava com a seta, para um lado ou para o outro e descobria.

**Mod. – As setas nesse sentido ajudavam. E em algum momento sentiu que não sabia exatamente o que lhe estava a ser pedido para saber. Que a instrução que lhe era dada, não era clara?**

**F. –** Não, acho que era claro.

**Mod. – Aqueles balões que apareciam, conseguiam explicar-se bem? E quando virávamos a imagem, alguma vez sentiu dificuldades em focar, porque a imagem se estava a mexer, ou tonturas, alguma náusea? Não?**

**F. –** Não.

**Mod. – O que gostou mais e menos desta plataforma?**

**F. –** Não acho que ela seja muito realista. Para ser franca, não gostei muito. Também nunca usei outra, não tenho termo de comparação.

**Mod. – Do que estava à espera, do que estava à espera de ver nesta plataforma?**

**F. –** Pensei que fosse uma maneira de ajudar as pessoas já no próprio supermercado e não a desenvolver capacidades cognitivas.

**Mod. – Portanto, uma plataforma de shopping online?**

**F. –** Online ou presencial. Sim, como eu lhe disse, as pessoas vêm perguntar para ler isto, algumas nem sabem ler. Infelizmente, isso ainda acontece. O que lhe disse, está lá em cima, acontece a muita gente. Eram mais situações práticas que estava a pensar.

**Mod. – Deixe-me só ver se eu consigo concretizar e perceber ao certo a ideia, seria uma plataforma que a pessoa vai ao seu supermercado e através dessa plataforma ela conseguisse ter direções para saber onde o produto está, é isso?**

**F. –** Não necessariamente. Até porque… isso também, agora passou-me o nome pela cabeça. A plataforma poderia ser entregue às pessoas à entrada do supermercado, não sei. Tenho uma ideia… Eu penso que esta plataforma ajudasse a disposição do, das mercadorias, para as pessoas poderem fazer as compras. Outra coisa, que tem a ver e não tem, eu conheço várias pessoas, algumas até são mais crescidinhas do que eu, na casa dos oitenta. Eu sei que a nossa lei nesse aspeto mudou, em termos de caixas prioritárias… acho que atualmente não é obrigatório estar lá caixas prioritárias, alguns supermercados têm, outros não. Por acaso, conheço uma senhora com quem falo bastantes vezes, e a senhora vai muitas vezes às compras, põe tudo no carrinho, mas fica um bocado aflita, acho que tem essa dificuldade…

**Mod. – Fica aflita para?**

**F. –** Não pode esperar.

**Mod. – Ah, sim.**

**F. –** E ela tem prioridade. Não sei como está a lei portuguesa. No outro dia andei a fazer essa pesquisa, mas… A lei existe e acho que as pessoas têm direito, mas as pessoas das caixas não estão informadas sobre isso.

**Mod. – Ok. E que transposição é que poderíamos fazer aqui para esta plataformas?**

**F. –** Uma coisa que me fez muita confusão, foi a esquina. Além, de no supermercado, pode estar vazio, normalmente quando tenho de ir ao supermercado, penso as horas. Agora não ter ninguém nas caixas.

**Mod. – E nos corredores, fez-lhe confusão? Não ter pessoas nos corredores.**

**F. –** Não, isso até é bom. A não ser que fosse alguém para ajudar. Era bom para as pessoas que vão fazer compras e para as pessoas que precisam de emprego. Isso devia haver também. No (informação sensível) tem aquelas meninas de patins e tal, estão sempre a mudar os sítios… acho que faz parte das técnicas de venda.

**Mod. – Provavelmente, não sei, mas…**

**F. –** Faz. Porque uma pessoa vai a um sítio, as bananas estão ali, no outro sítio as bananas já não estão lá. Tenho de dar a volta pelas outras coisas todas. E, entretanto, vai gostando disto e vai comprando. Mas… essas pessoas, às vezes ajudam. Aliás, agora há aí um anúncio na televisão, acho que é do (informação sensível), que é uma senhora que está a perguntar uma coisa a um senhor e o senhor…

**Mod. – Nem trabalha lá.**

**F. –** Exatamente. Mas ajudou a senhora.

**Mod. – E aqui seria interessante termos uma pessoa num dos corredores, em que fosse possível clicar e pedir ajuda?**

**F. –** Sim.

**Mod. – Essa ideia é muito interessante. Muito interessante. Acha que… isto pensando em alguém que… já começa a ter dificuldades em fazer estas compras no seu dia a dia, sozinha. Acha que esta plataforma poderia ser uma ajuda para este treino destas competências, no sentido de exercitar?**

**F. –** Se é sempre a mesma, acho que não.

**Mod. – Se é sempre a mesma…?**

**F. –** As mesmas figuras, as bananas sempre no mesmo sítio. Eles estão sempre a mudar. Isso até poderia baralhar as pessoas. Elas pensavam, as bananas estão à direita, chega lá naquele dia, não estão à direita, estão à esquerda.

**Mod. – Estou a perceber. Ok…estou exatamente, até poderia ser um elemento que confundisse ainda mais a pessoa porque no dia a dia as coisas estão dispostas de uma forma diferente daquela que aparece aqui? Ok. (informação sensível), agradeço imenso o seu tempo. Tem mais alguma sugestão que nos queira deixar?**

**F. –** Para já não. Se eu me lembrar de alguma coisa, mando um email.

**Mod. – Combinado. Muito obrigada.**

**F. –** De nada. Espero ter ajudado de alguma maneira.

**Mod. – Ajudou. Sem dúvida, com ideias muito interessantes e observações bastante pertinentes.**

**F. –** Obrigada também.

*Participant 6*

**Mod. – Quando quiser, pode começar.**

**F. –** Isto é uma plataforma, mas a plataforma pertence ao *Gmail*?

**Mod. – Não, não, são coisas distintas.**

**F. –** Está bem.

**Mod. – O *Gmail* eu até posso fechar o separador.**

**F. –** Não, não, era o que eu ia dizer, está ali…

**Mod. – Foi apenas para exemplificar o que é pretendido nesta primeira fase da sessão.**

**F. –** Ok, posso explorar?

**Mod. – Exatamente, quando quiser.**

**F. –** Eu para ir ao supermercado tenho que me inscrever…

**Mod. – Vou-lhe pedir que aí…**

**F. –** Sim deixe-me ver.… então, eu receitas ou listas de compras só quando tiver certamente lá dentro, será? Porque aqui está desativado…

**Mod. – Hmm.... Aí é participante13.**

**F. –** Escrevo?

**Mod. – Sim.**

**F. –** Elá… seu eu tivesse um… um… participante…

**Mod. – treze.**

**F. –** Sim.

**Mod - @gmail.**

**F. –** Arroba, onde é que estás… não está aqui. Não, pois não?

**Mod. – Não, deve ser… exato… tem ali…**

**F. –** Arroba. Er…

**Mod. – Gmail.com.**

**F. –** Pois, então.

**Mod. – E a password é participante13.**

**F. –** Hmm… não tenho mais nada. E agora? A realidade virtual, fazemos… o que fazemos. Deixe-me ver se posso ir às receitas, ah, agora já posso, já tenho receitas.

**Mod. – Exatamente.**

**F. –** Muito bem. E então, posso mexer?

**Mod. – Pode mexer.**

**F. –** E então… vou tentar fazer caril de frango [risos] Er… a diferença do caril está exatamente na especiaria. Portanto, isto aqui agora, se eu quisesse imprimir, não tenho diretamente… a impressora instalada, não é, tenho que ir procurá-la. É, não é?

**Mod. – Gostaria de imprimir a receita para poder fazer?**

**F. –** Por exemplo, sim.

**Mod. – Ok, neste momento não temos essa funcionalidade.**

**F. –** Ah, porque isto é importante, imprimir a receita. Quer dizer, tem de estar ligada a uma impressora, mas… ou tenho que decorar a receita, se não decorar a receita, tenho que imprimir.

**Mod. – Estou a perceber.**

**F. –** Agora vou voltar para trás, não sei se posso ir diretamente à lista de compras… ah, posso, por favor selecione uma lista de compras. Posso… criar uma lista de compras?

**Mod. – Pode.**

**F. –** Ah… vou criar uma lista de compras… portanto… é o que eu quiser? Escrevo o que eu quiser?

**Mod. – Sim.**

**F. –** Ah, por exemplo…e agora, onde é que eu… faço skip… escreva… produtos. Escreva o nome do produto, por exemplo… ah ok. Agora vou fazer aqui a lista, não é?

**Mod. – Exato. Esta ainda é uma versão inicial da plataforma, não temos todos os produtos, temos um conjunto mais…**

**F. –** Caril, caril em pó. 50 gramas. Então e se eu não quiser caril 50 gramas, se eu quiser mais, menos, mais caril… isto estou a partir do princípio de que há só uma variedade de caril, não é, nós estamos tão habituadas que haja mais caril…

**Mod. – Hmm Hmm**

**F. –** Eu pensava que estava aqui uma coisa do *enter,* mas não está, portanto deve ser esta.

**Mod. – Sim.**

**F. –** É ou não. A lista… a lista não é na vertical, portanto as coisas são seguidas?

**Mod. – Estava a fazer bem como estava. Apague… agora mais um. E se…**

**F. –** Este produto já se encontra na lista.

**Mod. – Consegue perceber onde está a sua lista de compras?**

**F. –** Onde é que está? Não…

**Mod. – Por exemplo, para ver os produtos que…**

**F. –** Aqui diz que já está, lista de compras. Só um momento… ah, se eu quiser remover. Mas eu agora só quero acrescentar.

**Mod. – Neste momento, não temos essa funcionalidade disponível.**

**F. –** Passar para a linha de baixo ou aumentar a quantidade. Aqui o objetivo…

**Mod. – Era a (informação sensível) poder pôr 100 gramas de caril. Mas neste momento a plataforma, nós não tínhamos pensado nessa vertente e a plataforma permite só selecionar produto a produto. Um produto de cada…**

**F. –** Está bem, não está mal pensado. Diga-me só uma coisa, esta lista vai para algum sítio para fazer a compra? ou é para eu levar comigo ao supermercado e ir…

**Mod. – Esta plataforma é um contexto estritamente digital. Não há uma transposição para a vida real.**

**F. –** Então está certo, é suficiente. Eu quero é passar para a linha de baixo, mas não dá.

**Mod. – Experimente apagar...**

**F. –** Sim…

**Mod. – Insira… vou-lhe sugerir que escreva bananas, por exemplo, imagine que queria comprar bananas. E agora pense noutra fruta…**

**F. –** Laranjas?

**Mod. – Por exemplo. Mas tem primeiro que selecionar. Tire mais um espaço…vai aparecer** **o tal menu.**

**F. –** Ah ok… bananas e passa para ali. Ah, eu pensava que ficava visível. Já entendi, bananas… deixe ver então. Laranjas… ok, fica verde, certo. Ok, muito bem.

**Mod. – Quer experimentar colocar mais um ou dois produtos na lista?**

**F. –** Pão…

**Mod. – Pão ainda não…**

**F. –** Arroz. Ah arroz agulha, arroz carolino, arroz agulha, arroz estufado, arroz biológico, arroz selvagem… arroz agulha, eu gosto de arroz agulha. Devia estar *basmati*. Arroz… está ótimo. Arroz agulha, não, não é. Peço desculpa… arroz… então… não me aceita aquele?

**Mod. – Viu o que dizia no balãozinho?**

**F. –** Arroz agulha…

**Mod. – Experimente…**

**F. –** Já se encontra na lista? Ah… ah… ele assume logo. Ok. mas esta… certo… agora, esta lista aqui… devia… a lista do lado direito devia começar a subir, porque senão… era muito mais intuitivo se esta lista começasse a subir. Está a ver, pode ser deficiência minha, mas agora vou colocar outro produto, por exemplo, caril… ah, já lá está. Agora, leite de cocô… não tem…

**Mod. – Temos bebida de cocô ou soja.**

**F. –** Certo.

**Mod. – Temos bebida de cocô.**

**F. –** Certo. Ah… dá logo ali. Muito rápido, pois… eu acho que isto devia aparecer ao lado. É muito fácil a pessoa não se aperceber se já inseriu ou não.

**Mod. – OK.**

**F. –** Se visse do lado direito, se ficasse visível era mais… que dizer...acho eu…

**Mod. – Era mais intuitivo?**

**F. –** Era mais, intuitivo.

**Mod. – Então vamos…**

**F. –** Agora já tenho aqui uma lista.

**Mod. – Tem uma lista de compras e vamos imaginar que quer entrar neste supermercado virtual, para fazer estas compras.**

**F. –** Sim.

**Mod. – Consegue perceber como é que faria?**

**F. –** Como é que eu faço a interligação com o site do supermercado. Pois, onde é que eu tenho guardada esta lista?

**Mod. – Sim…**

**F. –** Por exemplo… sim…

**Mod. – Vou-lhe pedir então que mexa na plataforma e… veja se há alguma coisa…**

**F. –** Eu tenho aqui… isto é um site?

**Mod. – É um site, exatamente. Ande para cima, para baixo.**

**F. –** Lista de compras, não quero criar nova lista. Quero ir àquela lista. Tenho de comprar…

**Mod. – Ele não assumiu, vamos…**

**F. –** Eu não a gravei. não é? Lista de compras, compras de fevereiro, não assumiu mesmo, mas está aqui o seguinte. Posso ir aqui? ah… ok. Desculpe, há bocado tinha ido. Vamos lá, agora já sei. Bananas… laranjas, caril… caril… e era arroz, não era…

**Mod. – Era…**

**F. –** Arroz agulha, ok. está a ver, quando eu falei há bocado. Agora, deixe-me cá ver onde é que isto está. Não sei onde é que está.

**Mod. – Então vou-lhe pedir que carregue no botão seguinte.**

**F. –** Ah, supermercado… produtos, está bem. Devia dizer, carregue, passe ao quadro seguinte.

**Mod. – E onde essa informação poderia estar?**

**F. –** No final da lista das compras, cá em baixo. Portanto, quando eu vejo a lista das compras, devia estar lá o botão a dizer seguinte, guardar ou… ir para o supermercado, não?

**Mod. – Não, não, faz todo o sentido.**

**F. –** Porque os que estavam lá… o seguinte em cima, é o que parece que a gente vai fazer logo a seguir, quando chega cá abaixo… então, insira aqui… isto é o supermercado? Ok…

**Mod. - Portanto, nesta fase aquilo que fez foi...**

F. – Organizar por… pelas diversas secções as compras. E agora…

**Mod. – Experimente…**

**F. –** Ah… fixe. Então… agora diz… hm… começar. Vou começar, não é?

**Mod. – Sim.**

**F. –** Estou no supermercado… esta coisa é gira. Eu não posso ver o que está aqui? por exemplo aqui… ah, os preços… fixe. Ok… então… toda a ação que realize corretamente, ganha um ponto. Tente acumular o máximo de pontos a cada nível. Pontos para quê? Já vou ver, desculpe.

**Mod. – Não, não… todas essas perguntas.**

**F. –** Responder aos nossos desafios. Então vou começar, não é?

**Mod. – Sim.**

**F. –** Já está aqui… não li o que estava ali. Aqui…compras de fevereiro, agora… o que eu disse, bananas e laranjas, compras de fevereiro… procurar… não…à vista… isto aqui é o quê, ah, ok… [risos] ah bananas… não, isto é só para ver a loja.

**Mod. – Então, vou-lhe dar uma ajuda. Porque… a fase, a forma como a plataforma está criada… está feita de forma que a pessoa primeiro vá para o separador supermercado, depois receitas e lista de compras. E nós assumimos que quem já está na lista de compras é porque conhece as etapas anteriores e está familiarizado com a forma de navegar no supermercado. E então… vou ter que… dar aqui uma ajuda. Consegue ver a seta que está no ecrã? Exato, clicar. Conseguiu perceber o que aconteceu ao clicar na seta?**

**F. –** Abriu a loja, não foi?

**Mod. – OK. Andou para a frente.**

**F. –** Ah, eu tenho que ir procurar o que eu quero, é isso?

**Mod. – Sim. E aqui fazia-lhe sentido, da perspetiva onde está, conseguir já selecionar as bananas, correto?**

**F. –** Sim, mas posso ir à lista? Posso ir, bananas, frutas… então…

**Mod. – Feche, pode fechar. Tem que ir até às bananas… se reparar está em corredores separadores, está neste corredor e as bananas estão neste. Se quisesse ir até lá, até às bananas… utilizando as setas… espere aí…**

**F. –** Eu tenho tempo. Ah, é esta. OK... Elas estão aqui.

**Mod. – Agora novamente gire a imagem, à procura das bananas…**

**F. –** Agora tenho esta.

**Mod. – Exato.**

**F. –** Ok, já estou ao pé das bananas. Posso? Ele vai aceitar… ah ok. bananas… 450 gramas, porquê? Se eu quiser mais…

**Mod. – Portanto, era interessante poder aqui regular a quantidade? De momento não temos isso disponível.**

**F. –** OK, esta já está. Agora…

**Mod. – Esse som foi sugestivo de que…**

**F. –** Está feito.

**Mod. – OK.**

**F. –** Agora, laranjas… laranjas, ok… não? Mas era suposto ele aceitar-me as laranjas…não…

**Mod. – Aqui, deixe-me só ver, dependendo da perspetiva onde estamos na plataforma, ainda não assume… experimente agora ver… ok. e já está. Agora…**

**F. –** Eu quero ir à lista outra vez…

**Mod. – Porque precisa de consultar a lista.**

**F. –** Ah... isto já esta. Ok... Agora, caril. Agora esqueci-me, onde procuro o caril? Só um momento… onde é que vou pôr caril... como é que eu sei que está nesta prateleira? Porque o caril é uma coisa pequena.

**Mod. – Mm, mm …**

**F. –** Não é aqui… não posso voltar atrás?

**Mod. – Pode.**

**F. –** Não é sair? Não… estará aqui? não…acho que já me estou a afastar.

**Mod. – Consegue perceber em que secções é que vai passando?**

**F. –** Aqui é bolachas e… aqui é produtos… de dieta, talvez. Mas eu tenho que andar pelo supermercado para encontrar as especiarias, não é? É que as especiarias muitas vezes estão em prateleiras pequenas, que é… que parecem produtos naturais, chás, etc… ah, poderia ser ali…, mas assim andamos a passear pelo supermercado. Aqui não está, aqui parece-me aquela parte inicial, é…

**Mod. – Aqui vou-lhe dar uma ajuda, também não conhece o supermercado. Continue em frente… mais uma…pode ser aqui, não sei… toque e deslize para a direita. Para o outro lado. Agora utiliza a seta, agora desliza para a esquerda.**

**F. –** Continuo?

**Mod. – Continue, assim mais um bocadinho, mais um bocadinho. E agora utiliza essa seta.**

**F. –** Ah, ok…

**Mod. – Aí vai conseguir encontrar também o arroz.**

**F. –** Aqui na… os supermercados normalmente têm placas, enquanto aqui diz paixão pelos alimentos, têm placas a dizer qual é o tipo de comidas que está naquele corredor.

**Mod. – OK.**

**F. –** Ou são os legumes frescos, ou os congelados, ou o arroz e não sei quê, as bebidas, enlatados, aqui não, não diz, pois não?

**Mod. – Não, não. O arroz está do seu lado direito.**

**F. –** Aqui posso ir já ao arroz, não é?

**Mod. – Exatamente.**

**F. –** Não... Mas devia… estou a encontrar várias dificuldades, já me estou a perder no supermercado, é o que eu faço habitualmente, passear pelo supermercado. Peço desculpa.

**Mod. – Não, não. Está a ir bem.**

**F. –** Ah, mas diz… aqui já diz charcutaria. Será que eu vou por aqui, aqui encontro…, portanto aqui é pão… não, não é por aqui que eu quero. Portanto, aqui não… ou por aqui, exato…já deve estar mais próximo… este aqui é os produtos naturais, outra vez… [risos] perdi-me? Não sei…

**Mod. – É normal porque não conhece o supermercado.**

**F. –** O supermercado, desculpe. Mas a gente já teve aqui. Fazer assim…

**Mod. – Vou-lhe pedir que experimente o primeiro nível que aí está.**

**F. –** Ah, este é muito mais giro. Agora não estou a fazer compras, estou só a passear pelo supermercado, não é? Maçãs..., mas eu não tinha, lá está, isto é a técnica dos supermercados. Eu não tinha pensado levar maçãs, mas posso levar. [risos] ah, ele não me deixa comprar o que eu não queria. E se eu quiser? Ok…

**Mod. – Ao ver o símbolo da lista de compras no balão, é intuitivo que pudesse carregar no símbolo e ele automaticamente abrisse. De momento não é possível.**

**F. –** Pois, já que está ali….

**Mod. – Faz sentido. E agora, sim, pode consultar a sua lista.**

**F. –** Pois, mas como é que eu acrescento à lista.

**Mod. – Agora também não....**

**F. –** Deixe-me ver. Adicionar ao cesto**.** Devia poder adicionar. Porque eu podia… posso não me ter… quer dizer, não precisava de maçãs, mas elas são tao bonitas que eu posso querer levar as maçãs. Que é o que acontece nestes sítios, ao invés de levar as bananas que estão muito maduras, levo outra em substituição. Mangas… posso levar mangas também, mas não me deixa também? Adicionar ao cesto… ah adicionou, boa. Porque é que não me deixou adicionar as maçãs?

**Mod. – Não estava na sua lista de compras.**

**F. –** Mas a manga também não estava.

**Mod. – Experimente consultar a lista de compras…**

**F. –** Mas não estava…

**Mod. – Neste nível há dois produtos, são as bananas e as mangas.**

**F. –** Ah… não estava na outra…

**Mod. – Na outra lista.**

**F. –** Certo, certo, certo, certo, certo, certo. Ok, então… agora quero as bananas. Ok… pronto. Para terminar as compras de hoje, dirija-se à caixa e carregue no botão pagar. Continuar…Ainda não… pagar, onde é que está o botão pagar.

**Mod. – Pode fazer o próximo, ou escolher…**

**F. –** [risos] Frutas... Mangas, meloas e tangerinas. Ok… então… para a direita…, mas era aqui… era tangerinas ou toranjas que lá estava? Está a dar-me aqui toranjas… Ops, não paguei...

**Mod. – Não aconteceu nada**

F. – Ah...frutas, legumes…. Acho que era esta...

**Mod. – Foi mais rápida do que o sistema, só isso. [risos] Carregue novamente.**

**F. –** É para continuar, não é.

**Mod. – Podemos fazer mais um ou ir ao nível das receitas para experimentar agora…**

**F. –** O que acha melhor?

**Mod. – Podemos passar às receitas.**

**F. –** Basta ir ali?

**Mod. – Certo e agora pode selecionar o creme da cenoura…**

**F. –** Aí é? hoje é o creme da cenoura? Também é muito bom… ah, agora vou às compras para a receita, é isso?

**Mod. – Exato.**

**F. –** Todos os ingredientes… ora bem… cenouras… curgetes, sal… azeite…eu tenho que… posso não querer mais nada….

**Mod. – De momento o sistema obriga a que todos os produtos sejam organizados, mas fazia sentido, então, que os produtos que não utiliza no seu dia a dia, que não os organizasse, correto? Está a falar da batata, não usa batata na sua sopa…**

**F. –** Não, não, para o creme de cenoura é que eu acho que… eu não colocaria batata nem pimenta preta, mas isso é outra questão. Mas se eu não tiver que os adicionar todos, tudo bem… eu acho bem que eles apareçam, até posso estar eu a esquecer-me de algum, acho bem que eles apareçam, mas eu posso é não os querer todos.

**Mod. – Estou a perceber. Olhando para essa secção das especiarias, há alguma coisa que lhe chame a atenção.**

**F. –** O sal está a vermelho. Quer dizer que não o devo usar, é isso?

**Mod. – Neste momento quer dizer que está na categoria errada. Porque… faz sentido, deveria estar nas especiarias e tem razão, mas nós é que organizámos…as categorias que aparecem são as secções do supermercado onde o produto está e não o produto… e não a categoria do produto. Está a perceber?**

**F. –** Mas sabe uma coisa, os supermercados hoje em dia têm algumas coisas que nos baralham. Por exemplo, os leites, eu não uso leite, o único leite que eu uso é o leite sem lactose. E o leite sem lactose não está nos leites, está junto às bebidas de soja, por exemplo no (informação sensível).

**Mod. – Na parte de saúde…**

**F. –** Sim… exato, mas não deixa de ser leite… portanto, se eu quiser leite sem lactose, onde me vai dar aqui?

**Mod. – Esta questão que nos coloca é muito pertinente e vamos…**

**F. –** Porque hoje em dia os supermercados estão a fazer coisas de maneira a nos chamarem a atenção, não é?

**Mod. – Sim.**

**F. –** E, por exemplo, o (informação sensível) … nós entramos, tem ali do lado esquerdo, tudo o que é pãezinhos, croissants, tudo o que está em pacotes, mas depois se quiser tostas, não está ali, está lá à frente ao pé dos produtos do dia, do momento, os croissants quentes e o pão. As tostas estão aí. Que na minha opinião fazia sentido estar tudo junto. Aqui eu depois não sei como vocês separam isso, porque os supermercados têm as técnicas de vendas, acho que é melhor por categoria de produto do que porque os supermercados… agora não sei, há para aí outra marca de supermercados novos, não sei se têm alguma técnica diferente de apresentar as coisas. Mas estou a deparar-me com essa situação deles separarem produtos da mesma família em sítios diferentes.

**Mod. – Em sítios diferentes, temos de ver, de facto o feedback que temos tido é que faz mais sentido organizar por categoria do produto, precisamente por essa questão que me está a referir. Por vezes eles separaram, mas outras vezes mesmo dentro…do produto, pode haver duas designações, não é, por exemplo, eu posso… não sei aquilo que me estava a dizer, os leites, eu posso ter leite de soja, não é, mas a bem dizer, eu posso colocar como bebida de soja. E aí já não entra na parte dos lacticínios.**

**F. –** Não, mas o leite sem lactose deveria estar junto ao leite.

**Mod. – Exatamente. Mas eles associam à dieta e aos produtos mais saudáveis…**

**F. –** Eu sei porque andei à procura, não acredito que o (informação sensível) não tenha leite sem lactose. Depois é que a senhora me disse, está junto aos produtos da dieta.

**Mod. – De facto faz mais sentido organizar. Neste caso e para conseguir passar à fase seguinte do supermercado teria de colocar o sal na secção do óleo, azeite e enlatados. Neste supermercado…**

**F. –** Como é que eu volto atrás? Ele não arrasta, pois não?

**Mod. – Não, arrastar não arrasta. Mas estava a fazer bem. E agora?**

**F. –** Ops, aqui vai para ali? não, vai para aqui. ah. Fixe.

**Mod. – Agora temos já…**

**F. –** Ah, isto é giro. O estar a vermelho chama-nos a atenção. Eu nunca iria era pôr os ovos junto ao azeite e enlatados, não é. ok… e agora… já estão, a lista de compras está feita, insira aqui… ingredientes… ah…

**Mod. – O finalizar ficou em supermercado.**

**F. –** Onde é que está?

**Mod. – Tem razão, é um erro do…**

**F. –** Ok. E agora o que vou fazer a seguir. Vou às compras?

**Mod. – hmm…**

**F. –** Er…começar…

**Mod. – (informação sensível) diga-me só uma coisa, são 11 horas… vai ter aula agora, não é?**

**F. –** Sim, mas… precisa de mais um bocadinho?

**Mod. – Sim, gostava de lhe fazer umas perguntinhas. Esta secção, depois no final da aula, se quiser passar por cá para concluir, tudo bem. Nesta… agora o que ia acontecer era, ia às compras, como foi anteriormente, mas com a lista…**

**F. –** A lista…

**Mod. – Que organizou.**

**F. –** Ah esta lista é muito interessante assim. e então? Ok…

**Mod. – Mas como lhe queria fazer umas perguntinhas e não lhe quero estar a roubar o** **tempo da sua aula, vou só tirar aqui…**

**F. –** Este processo na minha opinião é muito mais interessante que aquele de… do que o primeiro da lista de compras…

**Mod. – Da lista de compras em que faz a sua própria lista. Essa era uma pergunta que eu lhe queria colocar…**

**F. –** Mas, de qualquer forma, estamos a falar de receitas, mas eu posso querer, perdão, fazer compras que não são de receitas, produtos de higiene, produtos de limpeza, por aí.

**Mod. – De momento, nós, como ainda é uma versão experimental, temos só alguns produtos.**

**F. –** Podem é criar uma… como é que eu hei-de dizer, uma divisão para outros, ou diversos…ou… quando vai às compras, faz as compras todas, faz a lista e depois no fim… pôr outra para diversos, sei lá, que possamos acrescentar.

**Mod. – Mas seria interessante então poder ter acesso a todas as categorias, a todos o tipo de produtos que um supermercado tem, desde as questões da higiene, os champôs, os sais de banho, os produtos de limpeza de casa…**

**F. –** Sim… porque nós não compramos só produtos de consumo, pronto, para comer, para beber, mas também temos esses.

**Mod. – Quais foram as principais dificuldades que sentiu em utilizar a plataforma. o que é que teve mais dificuldade em perceber a utilização? Por exemplo, da navegação…**

**F. –** A navegação depois parece-me bastante fácil.

**Mod. – As setas são visíveis, consegue discriminá-las bem ou seria interessante termos algo mais chamativo?**

**F. –** Hm… acho que no final tem que nos dizer concluído ou… quer dizer, no final de cada fase tem que dizer, passe à fase seguinte, deveria ter uma seta do lado direito para termos a sensação que temos de continuar.

**Mod. – Que seguimos para a seguinte.**

**F. –** Esta parte está interessante. Agora falta a ligação à plataforma do supermercado para fazer a compra.

**Mod. – Íamos fazer agora se tivéssemos mais tempo.**

**F. –** Sim, sim.

**Mod. – Pensando nos níveis dos supermercados, na parte das receitas em que organiza apenas os ingredientes… nas categorias e na lista de compras, em que tem de introduzir os produtos…**

**F. –** Podíamos… não sei se não podemos associar as duas, pois não? São duas compras separadas.

**Mod. – Explique-me a sua ideia, o que seria interessante.**

**F. –** Eu tenho… vou fazer uma comida, mas eu tenho alguns ingredientes em casa. Eu só preciso… eu até me podia dar a lista e eu poder selecionar só alguns, eu não tenho que ir a todos… eu até posso… seleciono só alguns ou que me faltam ou até posso acrescentar, não estava na minha intenção, mas ao ver posso querer acrescentá-los.

**Mod. – O sistema ser flexível o suficiente para introduzir ou…**

**F. –** E ainda mais, quando eu estou a passear pelo supermercado, eu aqui vejo, ah, umas couves tão bonitas…posso querer acrescentar e ele dar-me a possibilidade, de eu ter aqui uma seta do lado direito a dizer mais… e eu… mais… e eu acrescento mais algum produto.

**Mod. – Quando vira a imagem ou mesmo quando avança, esta alteração da imagem… causa-lhe algum desconforto a nível de visão desfocada, sentir alguma náusea, tontura.**

**F. –** Não, até porque ela não vai muito rápido e eu tenho sempre a possibilidade de continuar ou voltar atrás. Não, essa parte parece-me bastante… bastante, lá está, intuitiva aqui.

**Mod. – O supermercado parece-lhe realista?**

**F. –** Bastante, sim.

**Mod. – Ok.**

**F. –** Embora isto seja um supermercado pequeno.

**Mod. – Tipicamente vai a grandes superfícies?**

**F. –** Não, a gente agora aproveita aqueles mais pequenos ao pé de casa.

**Mod. –** Pois... para aquelas compras de…

**F. –** até porque uma pessoa sozinha não precisa de fazer muitas compras. Eu já mudei o meu sistema de compras também, antes ia-se fazer muitas compras, agora é mais o que vai faltando. Porque se faz muitas compras, às tantas não consome e tem desperdício.

**Mod**. – E depois há a questão de ter de levar as compras.

**F.** – Pois, também. Quer dizer, ainda me desloco, vou… mais longe, quando tenho compras mais… mais especificas, vou a uma grande superfície e levo o carro, não é. Não preciso de ir ali ao pé de casa.

**Mod. – Mas ainda assim, há que tirar as compras do carro para levar para casa.**

**F. –** Sim, mas às vezes temos visitas e precisamos de fazer mais compras.

**Mod. – Como se sentiu a utilizar a plataforma? sentiu-se…**

**F. –** É interessante, sim. por exemplo, eu gosto de mexer… na fruta, gosto de mexer. Gosto… as mangas, os legumes, nos legumes, no fundo… nos legumes gosto de mexer. Mas numa situação em que a pessoa não possa sair de casa ou esteja com alguma limitação, ou pronto, alguma limitação… parece-me interessante. Desde que eles depois façam a entrega em casa. devia ter aqui esta possibilidade.

**Mod. – Como lhe disse, sito é uma coisa apenas no digital, não tem uma… não acontece…**

**F. –** Ainda não tem.

**Mod. – Não tem e esse não será o objetivo. Aqui o objetivo da plataforma é treinar esta competência de ir às compras, para quando a pessoa vai à sua mercearia de bairro ou a um supermercado que tipicamente utiliza, ser capaz de ter a sua lista de compras, ir lá compras os produtos que necessita, tentando não se desviar muito do orçamento para aquelas compras e… concluir então esta atividade.**

**F. –** Sabe que também… que nos supermercados mais pequenos já tem uma caixa para as entregas em casa. Normalmente é só uma.

**Mod. – Sim, sim… é algo que nós podemos ver. A questão é que nós aqui não temos, não estamos associados a nenhum supermercado.**

**F. –** Sim, sim, ok.

**Mod. – Imagine ou alguém que conheça que pudesse já ter aqui algumas dificuldades, a nível de memória, atenção, acha que esta plataforma poderia ser um instrumento para que a pessoa treinasse estas competências relacionadas à ida às compras e assim manter-se autónoma até mais tarde. Ou acha que a transposição daquilo que a pessoa faz nesta plataforma para o dia a dia dela, é difícil?**

**F. –** Não, parece-me até interessante, porque pode ser, pode ser estimulante. Ora… deixa-me ver… a pessoa até pode ir fazer aqui só como exercício de compras, olhe, deixa-me cá ver… por isso é que lá está, devia ter a possibilidade de acrescentar coisas. Eu ando a passear, não me lembrava disto, mas eu quero levar. Acho que sim, isto pode ser estimulante para a pessoa. Só tem aqui um problema, as imagens são fixas, se a pessoa muda de supermercado, não sei… imagine que passa para uma grande superfície, isto já fica um bocado limitador. Mas isso acho que não há volta a dar, vocês têm sempre de estar… tem de ter uma base para as imagens.

**Mod. – E falou-me também há pouco que seria interessante poder imprimir.**

**F. –** Sim, a lista das compras, sim.

**Mod. – Seria interessante a pessoa fazer a sua compra, vai ao supermercado e no final poderia imprimir a sua lista de compras para ir depois ao supermercado final.**

**F. –** Isso sim. Mas há uma questão que lhe gostava de lhe colocar, quando põe aqui os preços, como é que atualizam os preços?

**Mod. – A atualização dos preços é relativamente…**

**F. –** Este preço é sempre aproximado? Pode não ser um preço real. Convém escrever isso, porque a pessoa depois pode estar a fazer contas. Isto vai-me dar… assim, mas os preços podem não ser exatamente estes. Há fruta que muda de preço, se for do princípio de época, as frutas de época mudam muito de preço.

**Mod. – E faz sentido. O preço da plataforma é relativamente fácil de atualizar. Nós tentamos não seguir a tabela de preços de nenhuma das superfícies… até para não criar aqui outras questões.**

**F. –** Acho giro. Acho interessante poder navegar por aqui, porque estimula a organizar até a lista na nossa cabeça. É giro. E depois o ver, aí falta-me isto… vou passando… acho interessante, sim.

**Mod. – Essa…**

**F. –** Poder fazer uma lista de compras com isto, parece-me uma boa ideia. Por vezes ando lá em casa, o que me falta, o que me falta, se fosse aqui, se calhar… surgia-me mais facilmente… as faltas.

**Mod. – Ok, portanto… pensando, fazia-lhe sentido nós construirmos, cada pessoa construir a sua lista de compras, ir ao supermercado e à medida que vai andando pelos corredores, falta-me isto e falta-me aquilo, poder adicionar, vai fazendo as compras na plataforma…** **numa perspetiva lúdica...**

**F. –** Porque o facto de poder viajar pelos corredores já nos… lembra do que pode estar em falta.

**Mod. – E depois sim, ter esta possibilidade de a pessoa imprimir a sua lista de compras…**

**F. –** Sim… isso era bom. A gente facilmente associa a uma impressora.

**Mod. – Essa perspetiva é muito interessante e de facto não tínhamos pensado nela.**

**F. –** Até podemos levar… a plataforma ao sítio… qualquer loja agora de… não é só qualquer loja, qualquer loja imprime… acho que imprimir a lista de compras, será boa ideia… para mim. Há pessoas que não precisam.

**Mod. – (informação sensível), tem mais alguma sugestão que gostasse de nos deixar?**

**F. –** Não, para já não, isto para mim foi novidade, acho interessante …

**Mod. – Agradeço-lhe imenso o seu tempo e todas as sugestões que nos deu.**

**F. –** Peço desculpa se não sou mais criativa, mas...

**Mod. – Não, não, não.**

**F. –** Mas acho bastante interessante. Gostei mais deste sistema assim

**Mod. –** **Do supermercado e não da lista de compras. Inicialmente, o começar logo por fazer a lista de compras pode ser um bocadinho confuso**.

**F. –** O ver o supermercado, é engraçado, até parece, vou já começar. Não… é mais intuitivo, parece… como temos esse hábito de levar o carro, parece que está mais de acordo com o que se pretende aqui em termos de compras.

**Mod. – OK.**

*Participant 7*

**Mod. – Esta é a plataforma, pode explorar à vontade e sempre nesta ótica de nos dar a sua opinião e o seu feedback. Neste momento, o que é que…**

**M. –** (…) e mais ou menos… sim senhora. Bom, está explicado aqui o…pronto, e agora o que é que eu faço? É para começar, é para começar por aqui, não é?

**Mod. – Pode começar pelo supermercado, sim?**

**M. –** Eram os três…

**Mod. – Vai ter de puxar um bocadinho mais para cima. Mas então faria-lhe sentido que toda essa secção fosse interativa, não é? Que pudesse clicar logo na imagem do supermercado** **e entrar…**

**M. –** Pois… quer dizer é preciso saber, não é, é preciso saber se é uma coisa ou outra…

**Mod. – Mas o seu instinto foi esse?**

**M. –** Foi, foi.

**Mod. – Aqui vou-lhe pedir que coloque no email participante14. Clicando aqui, exato…**

**M. –** Então…

**Mod. – É no de baixo.**

**M. –** É no de baixo…

**Mod. – Agora, arroba, gmail. Ponto com. E a password é a participante14. No... no… ele não assumiu o r. Deixe-me só confirmar. Ele pode não ter assumido alguma letra.**

**M. –** Seria provável, eu não estou muito familiarizado…

**Mod. – Com este teclado. Mas também não há problema, é a única coisa que vamos precisar.**

**M. –** Mas estava certo?

**Mod. – Estava.**

**M. –** Sim senhora.

**Mod. – OK, aqui o que é que…o que é que vê?**

**M. –** Ora… não estou a perceber.

**Mod. – Qual é a dúvida?**

**M. –** Ah, ok, são os três, está bem.

**Mod. – Vou só voltar aqui atrás um bocadinho. Entrou no separador supermercado, correto. Hum.. e hã, tem aqui vários níveis. Vou-lhe pedir que comece pelo primeiro.**

**M. –** Ah ok, está bem.

**Mod. – Neste momento está à procura da seta, correto?**

**M. –** É, é.

**Mod. – Vou-lhe pedir só que feche o balão, o balão de diálogo, carregando continuar. E agora sim… consegue identificar a seta no…aqui.**

**M. –** Ah.

**Mod. – Ela está um bocadinho camuflada.**

**M. –** Ah, está ali, está bem.

**Mod. – Só com um clique, assim.**

**M. –** (…) se encontrava…

**Mod. – Agora ele está a pedir-lhe para tocar no centro do ecrã e deslizar a mão para a direita. Ok, até encontrar a próxima seta.**

**M. –** Aqui, não percebo. Consulte a lista, mas não me aparece nenhuma lista para consultar.

**Mod. – OK. experimente fechar essa caixa, carregando no continuar.**

**M. –** Ok, ok.

**Mod. – Clique novamente na fruta que estava a selecionar. Ok. Este som é sugestivo de alguma coisa?**

**M. –** É, é, deve ser… eu já gastei 50%.

**Mod. – Vendo na barra em cima?**

**M. –** Sim. vendo aquela barra.

**Mod. – Tinha de comprar dois produtos, correto, ou ele pedia para tocar em duas frutas. Eram as mangas…**

**M. –** As mangas e as bananas. Já toquei nas mangas.

**Mod. – Experimente agora tocar também nas bananas.**

**M. –** Adicionar ao cesto. E se eu não quiser adicionar?

**Mod. – Ok. neste momento o sistema está feito de forma que tenha de comprar esses dois itens para poder concluir.**

**M. –** Ah, vamos lá ver… isto.

**Mod. – Ok. A instrução que nos deram… exato.**

**M. –** Ali… depois há-de passar à caixa. Agora, agora… E… pois, isto é como todos os jogos, não é. er… não é…hmm… é preciso fazer o coiso, para perceber…

**Mod. – Podemos passar ao próximo**

**M. –** Sim senhora.

**Mod. – Então, se quisesse passar para o próximo nível.**

**M. –** Próximo nível…

**Mod. – Para o nível dois.**

**M. –** Esta seta não está uma coisa muito… primeiro porque é verde. Não…

**Mod. – Neste momento o que é que… procura?**

**M. –** O nível dois…

**Mod. – Eu posso-lhe indicar. Nesta fase o que é que acha que está a ser mostrado?**

**M. –** Eu penso que estão aqui… mas eu não comprei nada neste… estão aqui os preços.

**Mod. – OK.**

**M. –** Estão aqui os preços, julgo eu. Mas tinha que estar aqui, não é. Isto são euros, é preços… agora é preços de quê? Do quilo?

**Mod. – Estou a perceber, estou a perceber a sua dúvida. Isto é a nossa lista de compras. Os itens que neste nível teremos de comprar. E aqui está só esta indicação, do que o que tem de comprar, não está ainda o que comprou.**

**M. –** Então o preço, o preço não precisava de estar aqui. Eu vou comprar… eu não vou comprar mangas a este preço, vou comprar mangas ao preço que elas são.

**Mod. – Estou a perceber. Então vamos fechar a lista de compras. Carregou no botão sair. E… deixe-me só… Eu aqui vou-lhe só… voltar à perspetiva onde estávamos. E daqui se quisesse selecionar os produtos que tem na sua lista de compras, como é que faria?**

**M. –** É o primeiro problema. Para já, precisava de ter aqui no cantinho a lista de compras.

**Mod. – Mas pode sempre no item…**

**M. –** Ah, ok. Mangas, melão e tangerinas. Mangas… Ok, 500g adicionar ao cesto. Eu ia comprar meloas…E tangerinas.

**Mod. – Vou-lhe pedir que volte novamente à sua lista de compras. E que me diga que produtos é que já comprou, se já comprou todos, se ainda não comprou, se lhe falta comprar alguma coisa.**

**M. –** Então já estão riscados e tudo. Era essa a minha lista de compras.

**Mod. – Então vamos concluir o nível. Está a tentar aceder à lista de compras, não é? vou-lhe pedir que feche o balão de diálogo, carregando em continuar. E, agora, para irmos até à caixa, vou-lhe pedir que siga essas setas. Qual é…hum… vai escolher a opção.**

**M. –** Não é?

**Mod. – Experimente outra.**

**M. –** Já me posso ir embora.

**Mod. – Até ao momento, o que é que achou?**

**M. –** Er… para quem está habituado a jogos deve ser fácil. Eu penso… não é uma coisa que eu pratique.

**Mod. – O que achou da navegação, da forma de navegar na plataforma?**

**M. –** A cor, aquela cor…

**Mod. – A cor da seta. Que cor é que poderíamos utilizar que fosse assim, que saltasse logo à vista?**

**M. –** Ia para o amarelo.

**Mod. – Amarelo, ok. e a forma de navegar, o ter de utilizar as setas, é fácil ou seria melhor termos outros…**

**M. –** Acho que é fácil. Houve ali uma que eu não percebi, não vi seta nenhuma, só depois de a procurar… mas isso faz mesmo parte. Quer que eu continue?

**Mod. – Vou-lhe pedir que explore agora os outros dois módulos de jogos.**

**M. –** Não é aqui… isto são os níveis, onde é que estão os módulos. Supermercado, receitas, Lista de compras… criar uma nova lista.

**Mod. – Vou-lhe pedir que clique nesse botão. [o entrevistado atende o telefone] Aqui estamos numa atividade diferente, não é?**

**M. –** Sim, sim, começa logo… escreva o nome da lista de compras. Já agora, vou-lhe dizer, eu passo uma ou duas vezes por semana, quando vou às compras, faço uma lista de compras. Nunca pus um nome numa lista de compras…

**Mod. – É uma…**

**M. –** Nem sei que nome lhe havia de pôr. Lista de compras? Não preciso… sabe como eu uso? Uso aqueles post-its, tenho um post-it, escrevo aí, colo na coisa do carrinho, a caneta na mão…

**Mod. – E vai riscando à medida…**

**M. –** Vou riscando e vou metendo outras coisas que me apetece.

**Mod. – Que vão surgindo na altura, não é?**

**M. –** Os chocolates… cada vez que passo lá, mesmo que não tenho na lista.

**Mod. – Sabe sempre bem, um bocadinho.**

**M. –** Não, repare… agora como pediu, nome da lista… eu não sei que nome havia de chamar. Lista de compras, é o único nome possível.

**Mod. – Ou compras1, só para termos aqui uma…**

**M. –** Er…

**Mod. – Por exemplo…**

**M. –** Por exemplo…

**Mod. – Portanto, carregou seguinte. Fazia-lhe sentido que ao clicar, ao escrever… ok. Neste momento, para adicionar os produtos é aqui em baixo, nesta secção. E o nosso supermercado ainda não tem tudo, mas temos algumas frutas, massas, arroz, temos leite. Portanto, dentro destes produtos… temos legumes também.**

**M. –** O que é que eu vou comprar…

**Mod. – Não temos, ainda só temos queijo ralado.**

**M. –** Só queijo ralado. Ok, eu ia colocar o queijo que mais gosto.

**Mod. – Se quisesse selecionar queijo ralado, como faria? Ok…**

**M. –** Tenho de pôr aqui queijo outra vez.

**Mod. – Ele aparece deste lado, queijo ralado.**

**M. –** O que quer dizer isto…

**Mod. – Coloque novamente queijo ralado. Falta um U... a seguir ao Q… clique nele. E… ele vai aparecer ali daquele lado.**

**M. –** Pronto.

**Mod. – Vamos experimentar introduzir mais um produto, uma fruta por exemplo. O que é que não… o que está a ser estranho neste momento?**

**M. –** Ter aparecido aqui… aqui com um, dentro de uma bola encarnada, com uma seta, para eu poder. Não é difícil… para eu poder eliminar, se eu depois não quiser, quiser trocar por outro. Quantos produtos são… são mais?

**Mod. – Podemos pôr as bananas e terminamos esta lista. Tire mais um espaço... ok, todos os produtos que introduziu, não é, vamos passar à fase seguinte.**

**M. –** Das compras

**Mod. – E agora aí… o que é que lhe sugere que tenha de fazer?**

**M. –** Tenho que ir… tenho que ir para as secções, primeiro das frutas… Supermercado… devia ir para a secção das frutas.

**Mod. – Clicando no nome da secção, iria aparecer na secção. Neste momento é para organizar os produtos dentro de cada uma das secções, como se estivesse a organizar a sua lista de compras. Se quisesse, por exemplo, colocar as bananas dentro da secção das frutas… clique nas bananas… e agora clicando na secção das frutas…**

**M. –** As laranjas fugiram. Ah, as pequenas laranjas. Ok… não estava a… então…

**Mod. – Já temos então a lista organizada. E, agora, sim, iriamos para o supermercado. Ok… exato.**

**M. –** Há bocadinho eram tangerinas, agora devem ser laranjas.

**Mod. – Experimente. Pois, mas se reparar, está num corredor e elas estão noutro. Teria de ir até elas… Aí terá que se chegar um bocadinho mais à frente. Exato… e agora virando a imagem, rodando-a. Mais um bocadinho… mais… ok.**

**M. –** Isto não permite andar para trás…

**Mod. – Experimente virar a imagem. E neste momento está… o que está a fazer?**

**M. –** Estou à procura da secção do queijo. Deve ter achado… são lacticínios. Já vi os iogurtes, conheço bem. E agora o queijo…

**Mod. – Carregou na lista de compras sem querer. Pode fechar. Eu aqui posso-lhe dar uma ajuda, porque não conhece o supermercado. Anda em frente…**

**M. –** Este não conheço não.

**Mod. – Penso que tem de andar mais uma vez em frente. E agora rodando a imagem… mais um bocadinho. Vai ter aqui nesta prateleira…Este está… esta…**

**M. –** Esta não conheço…

**Mod. – Portanto, eles deveriam…**

**M. –** Ah, certo…

**Mod. – Daria para aumentar. Por algum motivo o sistema não está a permitir selecionar mas…**

**M. –** Estaria algures aqui. Eu também não… queria comprar já…

**Mod. – Vamos agora sair daí, se quisesse sair…**

**M. –** Não achei o queijo. Acho que sim…

**Mod. – Agora se quiséssemos explorar o último módulo de jogo.**

**M. –** É este?

**Mod. – As receitas…**

**M. –** Ah…

**Mod. – Portanto, com o dedo está a deslizar nas imagens para escolher uma. Vamos experimentar o creme de cenoura. Ok…**

**M. –** Creme de cenoura…

**Mod. – O que é que acha que tem para fazer nesta fase?**

**M. –** Tenho que… são os ingredientes, tenho de escolher os ingredientes para o creme de cenoura.

**Mod. – Vou-lhe pedir só que leia a legenda por baixo dos ingredientes.**

**M. –** O que é que preciso mais… tenho os ingredientes todos.

**Mod. – Está a selecionar aqueles que precisaria. Neste momento a plataforma precisa que organize todos os ingredientes.**

**M. –** Pela ordem de entrada?

**Mod. – Pela mesma lógica que organizou esses. Esteve a colocá-los dentro de cada uma das categorias, correto? É fazer o mesmo para os restantes. Mas aqui o seu instinto é selecionar apenas aqueles que lhe faziam falta, ou que lhe fariam falta se fosse uma situação normal. Já está, mas… vou só incluir…**

**M. –** Ah, tem de ser todos?

**Mod. – Todos.**

**M. –** Ah, ok, ok. Não tinha percebido. Estava só a pôr os que eu levaria. Sal…porque é que sai a vermelho?

**Mod. – O que é que lhe sugere isto?**

**M. –** Que não é especiarias e condimentos.

**Mod. – Se nós o quiséssemos incluir por exemplo na secção de óleos, azeite e enlatados, como é que faria?**

**M. –** Acho que faria mal.

**Mod. – Mas se quisesse fazer a ação, como é que faria?**

**M. –** Ah…

**Mod. – Exato. Deixe-me só explicar-lhe esta parte, as categorias que nós apresentamos, são as categorias ou as secções do supermercado, onde o produto está e não a categoria do produto. E, portanto, pelo aquilo que percebi, fazia-lhe mais sentido que fosse a categoria do produto, não é? o sal fosse efetivamente para as especiarias…**

**M. –** Para já não punha sal. Mas o sal está mais ao nível das especiarias do que o óleo e o azeite.

**Mod. – Dois níveis, em primeiro lugar, só ter de organizar os ingredientes que usasse e por outro lado ter as categorias do produto e não as secções do supermercado.**

**M. –** Sim, sim. até porque…o raciocínio sempre é que… quer dizer, vou àquele supermercado, não queria ir a outro. Fazia a lista, faço a lista, por acaso vou sempre ao mesmo, exatamente por causa… sei onde estão as coisas. E quando não estão no sítio delas… mas… mas vou lá, eu… o que eu faço, faço a lista.

**Mod. – Se calhar vamos passar à fase seguinte. Vou-lhe pedir qual é a sua impressão geral da plataforma, o que é que achou, o que gostou, gostou menos, coisas que acha que deveriam ser melhoradas.**

**M. –** Eu penso que a plataforma…o jogo… depois de usado ia ter uma coisa particular, que não tivemos aqui oportunidade de ver, que são os vários níveis. É uma plataforma interessante, um jogo interessante. Mas por isso, tem vários níveis, imagino que se começa no princípio e que se permite progredir o que é para mim importante.

**Mod. – Esta noção de progressão, de evolução.**

**M. –** Isso, isso. Como eu não jogo… não pratico estas coisas nem no computador nem no… Como é que se chama?

**Mod. – No tablet?**

**M. –** No tablet. Não pratico não… não acho graça aos jogos. Procuro outras coisas. Aquelas pessoas que vejo no barco, ali… [risos]

**Mod. – E a navegação, por exemplo, enquanto andava pelo supermercado?**

**M. –** Mudarmos a seta…

**Mod. – A seta amarela.**

**M. –** Não sei se é amarela, mas tem que experimentar. O verde de vez em quando confunde-se. Tem que ser uma coisa salte à vista mais… e o verde de vez em quando confunde-se com a hortaliça.

**Mod. – E quando mudava de imagem sentia dificuldade em focar, tonturas, náuseas?**

**M. –** Não, não, isso está muito bom. Focar… aquele processo… é perceber o processo de andar, carrega na seta e depois aparece o círculo castanho. Tem que esperar que o círculo castanho coloque as coisas no sítio. O que é que é preciso… perceber essas coisas.

**Mod. – E a nível dos balões de diálogo, daquelas caixinhas de texto que apareciam. Acha que a seguir…**

**M. –** Essa parte parece-me bem.

**Mod. – E a nível dos sons, era claro quando os sons… quando acertava, quando errava, quando colocava os produtos, não sei se se está a lembrar, surgia um som…**

**M. –** Não sei de som nenhum. Sou um bocado surdo.

**Mod. – Entre aqui... que penso que… as cebolas. Só para não ficar com a dúvida. Este som. Portanto, passou-lhe despercebido?**

**M. –** Passou despercebido…porquê, vou-lhe dizer porquê, porque isto é o som do computador quando desaparece, aparece um email, faz qualquer coisa no computador, aparece este som. É um som…

**Mod. – É um som normal…**

**M. –** É o som corrente, não é o som do jogo.

**Mod. – Estou a perceber. Essa informação…**

**M. –** Agora que me pôs o som, tenho o computador ligado, estou ali a fazer qualquer coisa, a escrever, por exemplo, entra um mail e ele faz-me este som. O mail ou outras coisas. Normalmente é uma coisa desse género.

**Mod. – Vamos pensar num som que fosse mais distinto…**

**M. –** Eu estou a falar por mim. Eu estou um bocado surdo, ouvi o som, ouvi, mas não liguei.

**Mod. – Na altura não associou a que fosse alguma coisa do jogo.**

**M. –** Não associei ao jogo. Como estava com o computador à frente não associei, para mim este é o som que está sempre no computador. Eu tenho o computador aqui, estou a escrever…e o computador está sempre a cantar este som.

**Mod. – Estou a perceber. Algo que fosse mesmo…**

**M. –** Para mim ignorei-o… quando fiquei assim, som, mas qual som? Não ouvi som nenhum. Claro que ouvi, sou um bocado surdo, deste ouvido não oiço, deste oiço assim assim. O som ouvi perfeitamente, não interiorizei, não liguei nenhuma ao som. Para mim era um som de estar a bater teclas…

**Mod. – E em relação à lista de compras, o que achou da forma como apresentamos a lista de compras?**

**M. –** Eu faria de forma diferente, que era pôr a lista de compras, todas umas por baixo das outras.

**Mod. – Os produtos, não é?**

**M. –** Produtos…

**Mod. – Não, não.**

**M. –** Os produtos uns por baixo dos outros e depois associá-los. Isso é curioso, quando faço a lista de compras, procuro pôr associados à localização, e a localização está ligado ao tipo de produto. As frutas ponho-as todas juntas, o queijo, o queijo, queijo… queijo roquefort, queijo de azeitão, queijo fresco, e requeijão. Isto não… roquefort e de azeitão não é todos os dias, nem todas as semanas. Agora os outros dois é todas as semanas. E o outro é quando falta. É assim que faço a lista, queijos, não ponho lá queijos, mas ponho na lista queijo de azeitão, roquefort, requeijão, queijo fresco.

**Mod. – Organiza por secção, não é?**

**M. –** Por secção.

**Mod. – E acha que este exercício ao nível, da… que nós tivemos nas receitas. Assemelha-se a esse exercício que faz em casa. Colocar as cenouras, batatas, curgetes, cebolas, tudo na secção, acha que isto faz sentido?**

**M. –** Sim, sim, sim. Agora eu faria… não faria dessa forma, pondo um aqui e um ao lado. Mas ai à lista assim, fazia a lista como lista.

**Mod. – Estou a perceber.**

**M. –** Então, se vou fazer a lista num papel, é assim que faço. Quer dizer, ok, não escrevo secções, mas não faz mal nenhum estar aqui as secções… é um passo curioso. Realmente, você está a fazer uma lista, ter aqui as secções e pôr os produtos nas secções. Agora, não os punha era assim em triângulo.

**Mod. – Um ao lado do outro?**

**M. –** Fazia a lista assim.

**Mod. – Ok, estou a perceber. Diga-me uma coisa, tem mais alguma sugestão que gostasse de nos deixar? Pode mexer, pode ver mais alguma…**

**M. –** De momento… pois aqui está bem, os ingredientes, podem estar, até podiam estar… vou colocá-los aqui… havia de ser o que eu estava à espera. Estava à espera de secções, se quiser pôr aqui uma complicação adicional, é por ordem de entrada

**Mod - No supermercado?**

**M. –** Pois, no supermercado. Não, eu estava a pensar na ordem de entrada, estou a fazer uma sopa de cenoura, a ordem de entrada da cenoura.

**Mod. – Ah, já percebi.**

**M. –** A hora de entrada…

**Mod. – Na receita, na confeção.**

**M. –** Vou buscar primeiro aquele para cozer, vou buscar o outro para temperar. Vou buscar…isto tem uma ordem.

**Mod. – Era uma atividade que…**

**M. –** Era uma complicação adicional.

**Mod. – Organizar dependendo da ordem como… como utilizamos para confecionar a receita.**

**M. –** Estamos a falar nesta culinária… o outro parece-me bem. A lista de compras, eu fazia a lista de compras… ponha secções ou não, pode pôr as secções, mas punha a lista numa única coluna…

**Mod. – Assim…**

**M. –** Não tem aplicações para jogos, não tem…

**Mod. – Mas pretendemos isso, que esta experiência se assemelhe aquilo que a pessoa faz na vida real. Essa experiência que faz a lista de compras numa única coluna e não põe um ao lado do outro. Nesse sentido é um input valioso.**

**M. –** Tenho lá um blocozito de post its. Com ele não mão, vou à cozinha, abro os armários… vou escrevendo, normalmente costumo colar nas costas do telefone… quando chego assim, pego no carrinho, pumba, ponho logo ali à frente. Coloco a caneta na mão, risco. Às vezes tiro dois ou três e é assim que faço normalmente. Uma vez por semana… às vezes… venho aqui, venho para a livraria, venho sempre à procura de novidades. E cada vez que eu venho aqui, já que estou aqui… falta-me um… depois quando chego a casa, há sempre coisas… normalmente é na lista. Mas às vezes, até a parte… já não ligo à lista, chego a casa e faltam-me coisas. Não é nada de importante. Mas durante a semana… além das compras, venho… venho assim… ao cinema, cada vez que há um filme apetecível. E venho com mais regularidade ao… à (informação sensível) ou à… como é que se chama…

**Mod. –** (informação sensível)

**M. –** A (informação sensível), normalmente à (informação sensível), depois vou comprar à (informação sensível), porque gosto mais.

**Mod. – E vai assim a estes sítios para se manter atualizado, manter as leituras atualizadas?**

**M. –** Tenho o panorama das coisas que saíram… pego numa mão cheia de livros e vou… com uma bica, um pastel de nata, é a minha receita, vou folheando livros, vejo livros, os autores… e às vezes como tenho uma data de livros para ler ainda, compro mais do que o que sou capaz de ler.

**Mod. – É o apetite, não é?**

**M. –** É.

**Mod. – Não temos só apetite por comida, temos também apetite por leitura…**

**M. –** Tenho lá mais livros do que sou capaz de ler.

**Mod. – Está bem senhor (informação sensível), mais uma vez muito obrigado pela sua participação. E se tiver alguma dúvida…**

**M. –** Espero que tenha sido útil.

*Participant 8*

**Mod. – Pode começar.**

**M. –** Não…

**Mod. – Pode… mexer a seta para cima e para baixo.**

**M. –** OK. Eu tenho a possibilidade de… de ver um pouco melhor o que é que isto faz, ou qual é o propósito, antes de fazer login?

**Mod. – Neste momento essa é a nossa página de apresentação?**

**M. –** Ah, ok.

**Mod. – Mas gostaria de ter mais informação?**

**M. –** Depende, depende, se eu, se eu já soubesse o que é que… ao que venho, iria progredir. Se a coisa fosse desconhecida para mim, queria saber um pouco mais do que é antes de aderir ou não.

**Mod. – Estou a perceber. Portanto, gostava de ter mais informação antes de se registar na plataforma?**

**M. –** Saber se é coisa que eu queira-me registar, que eu tenha interesse ou não.

**Mod. – E como é que nós poderíamos fazer isso?**

**M. –** Como é que poderíamos fazer isso…

**Mod. – Um vídeo?**

**M. –** Também depende, depende um bocado… de, eu não sei se estou, pode ser que eu não esteja a ver bem a coisa. Neste momento, a minha primeira, primeira reação é… isto aparece-me porque voluntariamente quero chegar aqui ou aparece-me e eu ainda não sei nada?

**Mod. – Ok.**

**M. –** E, dependendo desses estados, que podem ser pertinentes ou não…

**Mod. – Sim, sim, sim.**

**M. –** Eu já sei o que é e entro. Ou ainda não sei o que é, quero saber o que é antes de entrar.

**Mod. – Estou a perceber.**

**M. –** Antes de abrir.

**Mod. – Estou a perceber.**

**M. –** Não sei se é pertinente que me aconteça isto me chegar sem eu saber o que é. Se não é o caso, isso é suposto… quando as pessoas chegam a isto, já saibam ao que vão, este meu ponto de vista não faz sentido.

**Mod. – Hmm hmm.**

**M. –** Se for o caso…

**Mod. – Se estiver disponível na internet e alguém possa…**

**M. –** Disponível na internet e alguém me mandou uma sugestão, acredito nas sugestões, mas gostava de saber antes de…

**Mod. – Ter uma ideia mais concreta.**

**M. –** Porque normalmente a gente assimila inscrições… a receber montes de mensagens, que depois se calhar não quer, tem que ter o trabalho de estar a pagar, por vezes não é fácil declinar, tenho de estar a receber coisas que podem ser incomodativas ou não. Não sei se este meu ponto de vista inicial é pertinente, ou senão é.

**Mod. – É sim senhora.**

**M. –** Se fosse, eu talvez gostasse de ver um pouco, o que é que isto… o que é que isto faz, o que é que isto me vai dar antes de aderir.

**Mod. – Se registar. Estou a perceber.**

**M. –** Pronto… primeiro ponto de vista. Se estamos em condições de eu poder fazer isso, progredimos. Não é grave. Agora aqui, progredir, será fazer login.

**Mod. – Exatamente. Aqui vou-lhe pedir que escreva participante14, não fará um registo verdadeiro, é só para os efeitos desta sessão.**

**M. –** Sim. 14?

**Mod. – 14@gmail. E a password é participante14.**

**M. –** Não dá para ver os caracteres?

**Mod. – De momento não.**

**M. –** Pronto, vamos pensar que não me enganei. Vamos pensar que não me enganei… estamos dentro.

**Mod. – Exatamente.**

**M. –** Deixa-me espreitar aqui… ok… aqui?

**Mod. – Aqui ainda temos que… faria sentido que toda esta secção fosse clicável?**

**M. –** Sim, pois, às vezes facilita a gente clicar em cima da imagem como forma de progredir. Mas pronto. Este nível, estes níveis… pois, ok, não sei qual é a dificuldade disto. Vou para o nível médio…

**Mod. – Comece pelo primeiro.**

**M. –** Primeiro… portanto, agora aqui… começar… como não sei o que isto vai sair daqui, vou clicar em começar.

**Mod. – Exato. Ver se não desligo aqui nada.**

**M. –** Tenho que dar atenção ao que se está a falar?

**Mod. – Não, só aos balões de diálogo. Podemos também tirar o som se preferir.**

**M. –** Seta… aqui uma seta, mas não vejo uma seta.

**Mod -Vou pedir que feche o balão, esse balão… de diálogo.**

**M. –** Ah, ok.

**Mod. – Conseguiu identificar onde estava a seta?**

**M. –** Não.

**Mod - Ela está, um bocadinho disfarçada.**

**M. –** Ah, ok, ok.

**Mod. – Confunde-se um bocadinho com as cores.**

**M. –** Ok. A seta estava no sítio dos legumes, se não fosse do meu interesse legumes, se fosse o meu interesse conservas, eu podia mover a seta?

**Mod. – Aqui a seta é um elemento que permite ou pretende dar a indicação de como a pessoa navega. Neste caso, clica-se na seta para andar para trás ou para a frente, ou para os lados. Ah… pensando na sua questão, aqui o objetivo é simular um ambiente normal de supermercado, portanto teria de utilizar as setas passando pelas várias secções até chegar à secção que deseja.**

**M. –** Pronto, aqui estava uma seta junto aos legumes, se eu não tivesse interesse em legumes, mas em conservas ou peixes, eu teria que passar pelas várias?

**Mod. – Sim.**

**M. –** Ok.

**Mod. – Como um supermercado normal, não é, pensando no talho, costuma estar sempre ao fundo, raramente está na entrada dos supermercados, tem que passar pelas várias secções até chegar ao talho e é isso que estamos a tentar simular também. Ao invés da pessoa clicar num botão e aparecer automaticamente na secção onde está, tem que passar pelas várias secções.**

**M. –** Ok. Portanto, e agora tenho que ir andando, se este não fosse o meu… não fosse do meu interesse, mas se calhar tenho de explorar, interessa que eu explore isto. Mas se eu parar… se este não fosse do meu interesse, tinha de ir clicar na seta até chegar onde eu quero, e aí parava?

**Mod. – Exatamente.**

**M. –** Fazia as compras nesse… as compras, pressuponho que isto é para fazer compras.

**Mod. – Exatamente.**

**M. –** Então vamos fazer esse exercício. Vamos imaginar que eu quero ir diretamente a correr às conservas. Eu penso…

**Mod. – Hmm Hmm**

**M. –** Eu penso... Que… a mim… se eu quisesse utilizar esta plataforma para comprar qualquer coisa.

**Mod - Hmm Hmm**

**M. –** Começaria, digamos, facilitar-me-ia a vida, não ter de correr o supermercado para chegar onde eu quero. Tivesse uma secção onde eu escolhesse as várias áreas.

**Mod. – Estou a perceber.**

**M. –** E poder clicar em duas ou três e não ter que percorrer o supermercado todo. Isto para mim seria uma das vantagens e de não ir pessoalmente ao supermercado. Porque eu pessoalmente tenho de percorrer aquilo tudo, aqui comprava massa… er… conservas, bacalhau e por hoje é o que eu quero. Se é que este é o propósito do nosso…

**Mod. – Esta plataforma não serve para shopping online.**

**M. –** Ah ok.

**Mod. – ou seja, não é possível através dela fazer compras na vida real. Isto é para treino da atividade das compras, imagine alguém que… por algum motivo…**

**M. –** Quer fazer compras online.

**Mod. – Não, imagine que alguém na sua vida real já tem muitas dificuldades em fazer as compras, já se atrapalha com o dinheiro, já não sabe bem quanto dinheiro gastou, quanto dinheiro é que tem de receber, ou já tem dificuldade em organizar uma lista de compras, perde-se no supermercado, porque não se consegue orientar no espaço, não consegue perceber… onde é que já esteve e aonde tem que ir, é para o treino desse tipo de competências.**

**M. –** Ah, ok.

**Mod. – Não é uma plataforma de shopping online.**

**M. –** OK.

**Mod. – Isto não tem uma repercussão na vida real em termos das compras. Ah…**

**M. –** OK.

**Mod. – Tem uma vertente terapêutica e clínica exclusiva. Não há aqui nenhuma vertente comercial ou de marketing associada.**

**M. –** OK. E, então, deixe-me perguntar porque é que utiliza este exemplo do supermercado quando o propósito não é compras de supermercado?

**Mod. – Nós avaliamos…**

**M. –** É só para eu perceber o que é que estou aqui a fazer.

**Mod. – Nós, nós, há um conjunto de atividades estabelecidas, que são usadas como referência para estabelecer a funcionalidade da pessoa. E uma delas é a capacidade de ir às compras, existem outras, existe a questão da gestão financeira, o ser capaz de fazer a lide doméstica, o ser capaz de utilizar um telefone, portanto há um conjunto de tarefas que são estabelecidas como as tarefas básicas para determinar se a pessoa é ainda funcional ou não. Existem três níveis de funcionalidade, por assim dizer, aquelas que se referem à ida às compras, chamam-me atividades instrumentais de vida diária. Há as básicas, como por exemplo, ser capaz de tratar da própria higiene, há estas instrumentais que referi há pouco, o telefone, as compras, a lide doméstica, e depois há as complexas, em que cognitivamente são mais exigentes, portanto requerem mais etapas para conseguir executar bem a tarefa. E este é o motivo pelo qual apresentamos o supermercado, por um lado é uma tarefa comum à maior parte das pessoas, temos todos que ir às compras, ou pelo menos a maioria de nós tem que fazer esta atividade, não tem quem faça por eles. Por outro lado, em termos cognitivos, intelectuais, é uma tarefa muito rica porque nos permite trabalhar com muitas coisas, permite-nos trabalhar com as questões do cálculo, a pessoa ser capaz de estabelecer um orçamento, ser capaz de o cumprir ou não, há a questão da gestão da própria lista de compras e planeamento, o que me falta, o que não me falta, a organização da lista de compras, porque já envolve outro tipo de competências. Esta capacidade de orientação no espaço, quando pensamos em síndromas demenciais, está afetada, a pessoa não se consegue orientar no espaço. E, portanto, aqui o nosso objetivo é, em pessoas, que já começam a apresentar estes défices, poder criar um instrumento que os estimule estas competências para que eles consigam até mais tarde manter-se funcionais. A sua presença aqui é como, como eu disse há pouco, um aliado, no sentido de… ok, se isto para uma pessoa saudável apresenta determinado tipo de dificuldades, para com uma pessoa com dificuldade vai-lhe apresentar muito mais. Portanto, queremos assegurar que esta plataforma é usada por utilizadores saudáveis, para depois podermos passar a uma fase seguinte.**

**M. –** Ok.

**Mod. – Portanto, o senhor (informação sensível) aparece aqui nesta fase intermédia, de pessoas saudáveis, a viver na comunidade, que nos vão ajudar a identificar aqueles pontos que até nesta fase já podem constituir dificuldades à utilização. Como o feedback que me deu há pouco de… se eu entrar aqui no vosso site, eu gostava de ter mais informação antes de me registar. Isto é uma informação importante para nós. Não é… se alguém já sente esta dificuldade, se pensarmos nesta pessoa com mais fragilidades, menos à vontade com a tecnologia, então temos de ser mais explícitos naquele que é o âmbito desta aplicação. E tornar claro que isto não é uma plataforma de shopping online, mas sim uma coisa para estimulação cognitiva de âmbito terapêutico e clínico.**

**M. –** OK…, mas… seja como for, é uma…é um shopping online que estamos aqui a jogar. E de algum modo sinto-me…no dever de dar os inputs relativamente a esta aplicação e aos meus interesses pessoais. Poderá ser-me útil fazer uma passagem pelo supermercado, como recolha de ideias daquilo que me vai sendo apresentado. Se calhar nem sabia que precisava disto e até preciso. Nesse aspeto está perfeito. Se eu já tiver uma noção do que eu preciso de compras, ou se já tiver feito uma lista, para mim seria mais produtivo chegar aqui e clicar, peixaria…

**Mod. – Lacticínios, frescos…**

**M. –** Frescos, pica, pica, pica, no fim vejo quanto é que a fatura já vai, valido ou não valido, corrijo, acrescento mais qualquer coisa, tiro alguma coisa e toca a andar. Seria para mim o mais adequado.

**Mod. – Fazia-lhe mais sentido.**

**M. –** Mas também não é mau a gente ter que passar pelo supermercado, às vezes vem e isto acontece muito na vida, ao passar por certos sítios nem se lembrava que precisava daquilo e está aqui uma coisa que precisa. Pronto, mas aqui estamos numa secção em que não vejo seta para mudar…

**Mod. – Vou só dar uma ajuda.**

**M. –** OK, ok. portanto, admitindo que eu afinal preciso de fruta… carrego no círculo da fruta ou da seta?

**Mod. – Experimente.**

**M. –** Ok. portanto, aqui não funciona. ah, ok… já me pediu a fruta. Agora quereria… por exemplo, estas maçãs. É assim o propósito do jogo, não é. O que é que eu faço?

**Mod. – Não reparou no balãozinho que apareceu, quando chegou aqui a esta secção? Ok, no balão dizia para clicar nas frutas, nas maçãs neste caso. Experimente clicar.**

**M. –** Ah, ok. adicionar ao cesto… não estamos a falar de quantidades, mas de artigos.

**Mod. – Hm, Hm....Seria interessante.**

**M. –** Penso que quando se adiciono ao cesto, quando vou às compras online, adiciono aquilo que eu vou comprar, ao cesto.

**Mod. – OK**

**M. –** Não o produto, mas uma coisa com uma quantidade certa, duas, três, quatro, cinco canetas.

**Mod. – Ou então o peso, não é?**

**M. –** Pois. E não canetas… pressupõe que eu depois vou dizer quantas canetas é que eu quero. Estou habituado a que… ou sinto-me mais cómodo, quando adiciono ao cesto, adiciono já três quilos de maçãs.

**Mod. – Estou a perceber.**

**M. –** Portanto, aqui seria maçãs. O que vai acontecer aqui depois é que a gente vai ao cesto e vai dizer quantos quilos de maçãs.

**Mod. – Experimente.**

**M. –** Antes de finalizar a compra vamos juntar mais qualquer coisa. Para onde é que vamos… ok, bananas, vamos às bananas… adicionar ao cesto, ok. Portanto, de frutas… vamos dizer que por hoje está. Vamos a outro sítio.

**Mod. – Está a fazer a sua, como se estivesse num supermercado. Ok…**

**M. –** Suponho que o propósito é exercitar o jogo.

**Mod. – É esse mesmo. Mas no balão que apareceu anteriormente, ele pedia-lhe para fazer duas ações, selecionar as mangas e uma outra fruta. Lembra-se?**

**M. –** Selecionei duas. Mangas e maçãs. No cesto devem estar… mangas e maçãs para eu ainda dizer quantos quilos de maçãs e quantos quilos…

**Mod. – Se quisesses arrumar essa parte, no ecrã o que é que… há algum símbolo que o leve a pensar que aqui pode perceber o que tem no seu cesto?**

**M. –** Seria útil… e funcionar com este jogo, que eu pudesse algures ir visualizando o que está no cesto. Er… daqui a pouco posso estar a esquecer se afinal pus bananas ou não pus bananas, se tivesse a visualizar com um desenho ou com uma lista. Eu já sabia que bananas já lá estavam.

**Mod. – Vou pedir que clique nesse primeiro símbolo. Nesse.**

**M. –** Ah, ok, está certo, está certo. Está aqui.

**Mod. – E, neste momento, o que é que já tem no seu cesto e o que lhe falta comprar?**

**M. –** Tenho bananas e mangas, como lhe tinha dito.

**Mod. – Falta-lhe comprar algum dos dois?**

**M. –** Algum dos dois? Não, estes dois estão na lista do que eu vou comprar. Estou a assumir que é isso. Só me falta definir que quantidades é que compro, quantos quilos de mangas e quantos quilos de bananas.

**Mod. – Se reparar na imagem, as mangas estão riscadas. Portanto…**

**M. –** Se calhar eu cliquei duas vezes, ou qualquer coisa...

**Mod. – Ao adicionar aparece na lista de compras riscado, com um indicativo que já tem isto no seu cesto. Esta é a lista de compras, os produtos que já selecionou aparecem riscados, como nós fazíamos, quando tínhamos a lista, comprávamos, riscávamos…**

**M. –** Ah, este risco é sinal de que já está tratado.

**Mod. – Exato. E neste caso falta-nos ainda comprar uma das frutas, que estão na nossa** **lista de hoje. Que são as bananas que ainda não aparecem riscadas.**

**M. –** E porque é que as mangas aparecem riscadas e as bananas não aparecem riscadas, eu não fiz…

**Mod. – Selecionou as mangas há pouco. Experimente clicar nas bananas. Ok… este som não está associado. Agora, adicionar ao cesto. Ouviu este som?**

**M. –** Sim.

**Mod. – OK.**

**M. –** É um som de que a coisa foi feita.

**Mod. – Exatamente.**

**M. –** Estão aqui dois. Este riscado é sinal de que ele… risquei da minha lista, como se tivesse riscado da minha lista, já tivesse tratado.

**Mod. – Exatamente, se já tivesse tratado, exatamente, exatamente. Já tratámos da lista dois, desta lista que nos foi dada neste nível, temos então agora de ir pagar.**

**M. –** Ou comprar, ou comprar ainda mais coisas.

**Mod. – Seria uma possibilidade, de momento o sistema só permite ir para as compras. Pagar, desculpe. Mas posso-lhe mostrar mais à frente um outro módulo em que cada pessoa pode fazer a sua própria lista de compras.**

**M. –** Então vamos finalizar.

**Mod. – OK. Seria o que…**

**M. –** Se não posso nesta fase juntar, sei lá... cerveja ou qualquer coisa assim, vamos pensar que isto são as compras de hoje.

**Mod. – Ok.**

**M. –** Eu se… se… ao estarmos com este jogo de compras de frutas e neste supermercado, penso que está por definir quantos quilos bananas ou… para si não é relevante, é só… dizer o que se compra e não interessa?

**Mod. – O sistema de momento não permite. Mas é um feedback que tem existido.**

**M. –** Para mim seria relevante, ninguém compra bananas, as quantidades que eles quiserem, é a quantidade que eu quiser.

**Mod. – Exato. É uma das sugestões que nos tem sido dada. É poder… manipular a quantidade dos produtos.**

**M. –** Se calhar, se calhar, para si não é propósito, eu estou a valorizar isto, se calhar é para valorizar… estamos a jogar aqui uma coisa de compras, de supermercado…, portanto, se o jogo é este, estes são os inputs pertinentes. Se o seu propósito não é tanto esse, se é outro, ok…, mas… como estamos a jogar em compras, teria todo o gosto, interesse, em definir a quantidade, que idealmente seria no momento de juntar ao cesto das compras, 3 quilos de bananas… daquelas bananas que eu vi àquele preço, porque estas são da madeira, estas são não sei de onde, os preços são diferentes. Eu optaria por uma delas, com preços diferentes e quantidades diferentes. Um quilo destas e meio quilo destas. Mas então, vamos finalizar. Uma das caixas, sim senhor. Então… aonde é que me aparece a caixa? Será aqui?

**Mod. – Experimente.**

**M. –** Aqui já percebi que é a lista. Esta agora não estou a perceber… a relação disto com… com as compras.

**Mod. – Os pontos. Então, eu aqui posso-lhe dizer que as caixas ficam em frente.**

**M. –** Ah, eu tenho de clicar aqui para irmos em direção. Às caixas? Certo?

**Mod. – Sim**

**M. –** Eu continuando a falar no jogo. Eu… teria acabado as compras e clicava logo na caixa. Er…

**Mod. – É um botãozinho que clicava e aparecia logo na caixa.**

**M. –** Por acaso a seta estava aqui perto das caixas, mas poderia não estar.

**Mod. – Sim.**

**M. –** Não devia andar a percorrer… para si é mais confortável apanhar aqui um botão para concluir e pagar.

**Mod. – OK.**

**M. –** Neste caso…eu devo clicar no boneco para sair ou aqui para ir à caixa? Eu fico…

**Mod. – Esse boneco dá-lhe a sensação que é para sair?**

**M. –** Sair…

**Mod. – Sair do jogo ou sair… ah, concluir o nível?**

**M. –** Concluir as compras. À falta de um que tenha a caixa, já cliquei em finalizar as compras, agora queria pagar. À falta de um objetivo que me fala em pagar ou em… em caixa… eu o que vejo mais perto desse ato é este. E para mim seria mais eficiente haver esse botão do que andar aqui à procura da caixa.

**Mod. – Neste momento…**

**M. –** Eu continuo…eu se calhar estou demasiado focado no jogo das compras online. E não devia estar tanto.

**Mod. – Todo o input que nos der é… são sugestões valiosas.**

**M. –** Estou um bocado focado, fui às compras online, está muito engraçado, mostra-nos as coisas, supermercado para comprar, as fotografias dos objetos está ótimo… o ter que passar o supermercado todo, poderia ser uma opção que eu escolheria ou não. A opção de passar pelo supermercado pode ser pertinente em determinados momentos, hoje eu não sei bem o que é que preciso, deixa-me cá passar pelo supermercado todo a ver, ou eu objetivamente tenho uma lista. E se tenho a lista queria clicar logo nos sítios, nas coisas que eu preciso. Cesto das compras… com as quantidades, respetivos preços…e no fim uma opção de pagar e sair.

**Mod. – Ok… para ver como nós temos essa parte do pagamento, vou-lhe pedir que clique na seta.**

**M. –** Sim… ok…

**Mod. – Assim dá-nos a sua opinião em relação a esta parte.**

**M. –** Não vejo aqui para trás.

**Mod. – Tem que se chegar mais para a frente, ainda não está bem nas caixas.**

**M. –** Ah… agora sim. se o jogo…é compras online, de supermercado… e aquilo que me deu para jogar, eu não gostava de ter este passo de me chegar mais à frente. Eu gostava que me aparecesse aqui a opção de pagar… porque isto, pá, não sei se calhar há pessoas que vão precisar disso, eu não precisaria. Com que então, vamos pagar. Multibanco…, portanto, não tenho que pagar, tenho de sair… próximo nível.

**Mod. – Vou pedir que experimente o nível dois.**

**M. –** Tomates, sim senhora. Agora quero… isto é alhos?

**Mod. – Experimente.**

**M. –** Alhos, sim senhora. O meu som… calhou há bocado.

**Mod. – Não é o mesmo.**

**M. –** Ah… não me apercebi, não tinha um som a confirmar… há bocado deu. Eu assumi que fosse o… realizado com sucesso. Vamos ver a lista. Eu não cliquei em tangerinas, nem meloas, nem mangas.

**Mod. – Isto é a lista que está associada a este nível, ou seja, tem de comprar estes elementos.**

**M. –** Ah, ok. mangas, meloas e tangerinas. Então neste nível não posso comprar aquilo que quero, só tenho estes três produtos…

**Mod. – Sim.**

**M. –** Vou procurar, mangas… meloas… e tangerinas. Não vejo aqui nem mangas, nem meloas nem tangerinas. Se calhar esta parte ajuda…

**Mod. – Tem a secção dos legumes.**

**M. –** Vamos lá ver o que é isto.

**Mod. – Esses aí ainda não devem estar disponíveis.**

**M. –** Este aqui é…

**Mod. – É um placard, sim.**

**M. –** É um placard. Então, estão aqui… frutas… frutas… disponíveis para eu comprar hoje e elas não aparecem aqui no…

**Mod. – Consegue ver algum elemento que lhe permita avançar e ver as outras secções do supermercado.**

**M. –** Eu posso ir para as outras secções, mas estou nas frutas. Eu suponho que ao mudar para outra secção, vou sair das frutas. Então… eu pensei que ia sair tangerinas, vamos a elas.

**Mod. – Têm que ser estas que estão mais perto.**

**M. –** Estas? Estas são laranjas.

**Mod. – Experimente. Se quiser sair daí como é que… fechar esse separador.**

**M. –** Era isto que eu estou à procura. Isto está fechado, ok. Aquele botão, não sabia que era para este coiso…

**Mod. – Se era para o separador ou…**

**M. –** Isto é tangerinas, ok. era tangerinas e era mais o quê? Meloas, ok… e era… mangas. Ok. transformou-se no cesto, aqui estão, mangas, meloas, tangerinas. Ok… finalizar... continuar…

**Mod. – Agora estamos na situação em que temos que ir até à caixa.**

**M. –** À caixa.

**Mod. – Pois... E aqui novamente dava-lhe jeito ter o botãozinho, pagamento, aparecia automaticamente…**

**M. –** Se fosse compras online, sinceramente…

**Mod. – Ok. Então, este já percebeu mais ou menos a mecânica.**

**M. –** mecânica.

**Mod. – Exatamente. Vou-lhe pedir se olhando para o ecrã se consegue perceber os outros módulos de jogo que nós disponibilizamos, há pouco falei de três, correto, já experienciou o primeiro. Se na plataforma… é fácil perceber estes outros dois módulos de jogo, como é que faria para chegar até eles?**

**M. –** Agora é escolher outro nível e avançar.

**Mod. – Olhando para o menu principal, esta barra. Temos o supermercado, não é, onde nós estamos e tem depois as receitas e a lista de compras. Estes são os vários níveis dentro do supermercado. As receitas é outro módulo e o terceiro será a lista de compras. Vou-lhe pedir que clique na lista de compras…**

**M. –** Criar uma lista…

**Mod. – Hmm hmm.**

**M. –** Eu posso… tenho uma lista, à qual dei um nome. Eu posso ir agora para o supermercado e…

**Mod. – Ainda tem duas fases intermédias antes de conseguir chegar ao supermercado. A primeira delas será colocar os produtos, para ver os produtos, nós ainda não temos todos os produtos disponíveis, temos frutas, temos massas, arroz…**

**M. –** Massas… se eu quisesse outra coisa diferente de massas… azeite?

**Mod. – Por exemplo. Mas se reparar, vou-lhe pedir que tire o S, só o último S…**

**M. –** Massa…

**Mod. – Agora falou-me em azeite, experimente.**

**M. –** Ah, ok. Tem aqueles azeites todos.

**Mod. – A forma como está, não é percetível que… ao começar a escrever há um menu que aparece com os vários itens disponíveis.**

**M. –** OK.

**Mod. – Agora vamos supor que são só esses três. A seguir como é que faríamos?**

**M. –** Talvez… queria validar isto.

**Mod. – OK.**

**M. –** Assim, assim…

**Mod. – Ok.**

**M. –** Tenho que ir ao supermercado?

**Mod. – Exatamente. Não acontece nada?**

**M. –** Não acontece… o que é que eu não estou a fazer para não acontecer.

**Mod. – Aqui nesta fase… lendo a descrição por baixo de produtos…**

**M. –** Por baixo destas coisas, clico as três. Ou não posso? Não posso…, portanto, agora aqui queria perceber se isto significa estar no carrinho de compras… se tenho que ir dar uma volta ao supermercado…, mas não está ativo.

**Mod. – Nesta fase, o que estamos a pedir é que insira cada um dos produtos dentro da categoria a que eles pertencem. Tem aqui os produtos, os três produtos que pôs na sua lista. E pedimos-lhe que coloque dentro de cada uma das categorias.**

**M. –** Ah, aqui. Este para aqui. Não tinha percebi isso… e agora vou ao supermercado?

**Mod. – E agora é que vamos ao supermercado.**

**M. –** E agora o que é que vou fazer ao supermercado se já fiz a lista, tenho que clicar nos produtos… que estavam na minha lista?

**Mod. – Hm, hm**

**M. –** Criei uma seta para eu me…

**Mod. – Há um passo inicial antes de começarmos o jogo, que é começar. Dar a indicação que queremos…**

**M. –** Então era… azeite, massa e… qual é que era o outro? Posso clicar aqui, não posso?

**Mod. – Experimente.**

**M. –** Azeite, massa e nos legumes… as batatas.

**Mod. – Desculpe, deixe-me só voltar atrás. Aqui houve alguma coisa que não… foi** **percetível a lista de compras?**

**M. –** Foi…

**Mod. – Senti que ficou ali a pensar um bocadinho. Houve algum aspeto da lista de compras que não tenha…**

**M. –** Não entendi porque é que eu tenho de associar azeite a tudo o que o supermercado tem na secção dos azeites.

**Mod. – A categoria não lhe faz sentido?**

**M. –** A categoria. Se eu já disse azeite… que seja a máquina a dizer que indexe é que pertence, se é que a máquina precisa disso. Eu não preciso disso. Eu, comprador, não preciso disso… dizer em que secção é. É azeite… se a marca precisa para o controlo de saldos, meter aquilo numa determinada base de dados, não sou eu que…

**Mod. – Não tem que se preocupar com isso, já compra… estou a perceber, ok.**

**M. –** Eu posso progredir enquanto não disse que azeite era secção de óleo, azeite e enlatados. E deveria poder progredir. Tal como as batatas é legumes e hortícolas e as massas é das massas… isto são secções de supermercado. Mas como lhe digo, eu se calhar estou demasiado focado no supermercado. Se não é para estar, peço desculpa.

**Mod. – Essa é a sua perspetiva e é isso que é pretendido aqui.**

**M. –** Ok. eu agora… ao vir aqui parece-me… que tenho que ir aos sítios e escolher as coisas nos sítios, quando já escolhi na lista, já disse na lista o que é que eu quero.

**Mod. – Seria uma simulação do ir às compras. Fizemos a lista, organizámos a nossa lista e agora vamos às compras.**

**M. –** ah, ok, ok. A gente quer massa, batatas… batatas acho que estão aqui. Vou já aproveitar o facto de estar na secção das batatas e comprar destas. Ah ok, estas estão. A seguir foram… não tinha batatas na lista. Ah tenho, na frutas e legumes, tenho. Falta o azeite e a massa.

**Mod. – Eu aqui vou-lhe dar uma ajuda, como não conhece o supermercado, para não andar perdido. Vou-lhe pedir que siga em frente, novamente em frente… pode ver de um lado para o outro, se quiser, aqui em frente. Vai seguir esse corredor até ao fim, até ao fim, até meio…e agora aqui vou-lhe pedir que se vire para a esquerda, até encontrar a outra seta.**

**M. –** OK.

**Mod. – Clica. Agora vou pedir que se vire para a direita. E que entre nesse corredor. E aqui vai poder encontrar o azeite e a massa.**

**M. –** O azeite está aqui. Não sei se está aqui o submetido, que lá mencionei…

**Mod. – Experimente chegar-se um pouco mais à frente. Exato… agora vou só fazer assim.**

**M. –** Ah, aqui estão. O que tinha escolhido era este. Se calhar é este.

**Mod. – Isto está com um bugzinho, não me está a permitir… não me deixa selecionar nenhum e não era suposto. Azeite… ok, então temos aqui estes… selecionar… vamos ver se ele agora nos deixa… não, não está.**

**M. –** Não.

**Mod. – Bem, vamos ver… ele está aqui com um problema, não me deixa… deixa-me selecionar as massas, mas…, mas não me deixa…**

**M. –** Olhe, não…

**Mod. – Não, isto devia estar tudo… afinal.**

**M. –** Azeite…

**Mod. – E agora estamos novamente na situação que se quiséssemos pagar teríamos de voltar…**

**M. –** Ir à procura da caixa.

**Mod. – Exato. Agora vou-lhe pedir que saia… e agora… rapidamente ver o último módulo que nós temos, que é o módulo receitas.**

**M. –** ah, ok. posso clicar?

**Mod. – Pode.**

**M. –** Ah, é tudo isto, tudo isto é… só falta é a carne. Ah, tem aqui. Portanto, clico nisto tudo. No fundo, penso que a aplicação me está a sugerir os ingredientes de uma receita, posso querê-los todos ou não. Como quero todos, valido todo, não tenho nada, não tenho polpa de tomate em casa, nem nada. Para esta receita. É este o propósito?

**Mod. – Não tínhamos pensado assim, mas é uma possibilidade. Neste momento é fazer um bocadinho a tarefa que fizemos na lista de compras, organização. mas aqui com…**

**M. –** Eu ao me listar isto tudo, que são as componentes totais da receita… tenho que os validar um a um, eu estou a pensar na utilidade de haver um que não precise. Tenho lá polpa de tomate em casa, não quero mais, quero tudo menos a polpa de tomate. Gostaria de funcionar assim. Azeite, para aqui… queijo ralado… continuo a dizer que esta parte da operação não me faz muito sentido. Lacticínios, massa, esparguete, está aqui, carne picada… está no talho, está certo. Os tomates no… aqui. As cebolas também seria ali, ou não? Ok… o sal…sal, arroz, farinha… não aparece… então, o sal não tem secção?

**Mod. – Será na secção dos óleos e do azeite, porque neste supermercado é onde de facto o sal está.**

**M. –** Ah, ok.

**Mod. – Mas talvez fosse mais intuitivo, ao invés da secção do supermercado fosse a categoria do produto e assim iria aparecer especiarias e condimentos.**

**M. –** Alhos... só pode ser… só pode ser aqui. Polpa de tomate… há-de ser (…) enlatados. Pronto… está certo. Agora temos tudo classificado. Vamos para o supermercado.

**Mod. – Vamos para o supermercado e agora iriamos seguir a mesma lógica de ir um a um,** **selecionar os produtos.**

**M. –** Um a um.

**Mod. – Então, terminámos esta primeira parte, vou-lhe agora pedir que me diga qual a sua impressão geral da plataforma, o que é que gostou, o que é que não gostou, já nos deu muitas sugestões enquanto foi utilizando, mas de uma forma geral, como é que foi a sua experiência ao utilizar esta plataforma?**

**M. –** Eu vou voltar àquilo que já disse. Se o propósito é eu me situar num supermercado e na possibilidade de fazer compras online…não vejo isto com a simplicidade que me seria útil, que eu gostaria. Vejo um pouco complexo. O facto de…, portanto, eu acho que…, portanto, seria simpático para mim ter duas opções, eu circulo pelo supermercado ou não círculo pelo supermercado, vou direitinho às compras e tinha de comprar aquilo que eu quero. Esta coisa das receitas está muito inteligente, muito útil. Uma pessoa quer fazer em casa uma bolonhesa e está a sugestão dos ingredientes, compra tudo ou compra só parte. Muito eficiente e muito útil. O facto de classificar os produtos, nas zonas do supermercado onde eles estão, ou nas suas categorias, não me parece que seja um trabalho que eu devesse fazer. Parece-me que… devia haver duas opções… eu já tenho uma lista das minhas compras já feitas e chego ali… vou objetivamente lá comprar. Ou… eu crio aqui a lista. E se eu crio aqui a lista… quanto muito, eu digo bananas, a aplicação devia-me levar a sítios onde haja bananas de duas ou três qualidades para eu as escolher. Eu disse bananas, mas não disse que é da madeira, nem sei se as outras que o supermercado tem, além de ser da madeira, se são do equador ou se são não sei de onde, levava-me lá, bananas neste supermercado tens estas opções. Madeira, equador, brasil…

**Mod. – Sim, sim.**

**M. –** E basicamente é isso. E teria mais facilidade em sair, em pagar para largar isto.

**Mod. – Ah, ok. E a nível da navegação dentro do supermercado, a utilização das setas, a possibilidade de se poder…**

**M. –** Não, isso está bem. Se eu quisesse usar a opção de circular pelo supermercado, isso parece-me que está eficiente.

**Mod. – A nível da caixa dos diálogos, a linguagem era clara, a instrução era dada de forma clara ou nem por isso?**

**M. –** Sim, sim, tendo em conta a forma como está feita, que não é a mais simpática para mim, para o meu gosto, parece-me bem. Obviamente que se tivéssemos outras opções, que não têm aquilo que eu acho uteis, há certas coisas que não fazia sentido. Andar a circular… se eu disser que quero massa, andar a circular pelo supermercado, que eu não quero, não faz sentido. Faz sentido se essa… é… opinável se for essa a escolha, se não for a escolha, não se chega a usar.

**Mod. – Ok.**

**M. –** Mas sim, mas está… se fosse esse o caminho, está eficiente, acho bem.

**Mod. – Quando mexeu na plataforma e virava a imagem ou avançava no supermercado, alguma vez sentiu, por exemplo, náusea ou tontura, ou sentir que a visão ficou desfocada? Sentiu algum efeito adverso enquanto navegava…**

**M. –** Sim, sim, estou a entender, isso pode acontecer. Não creio que seja relevante. Não creio que seja relevante, estas coisas também depende um bocado da vontade de cada um. Há pessoas que têm pressa, querem agir de depressa, outras reagirão com mais lentidão porque o muito depressa pode fazer tudo turvo. Mas a mim não me fez…, mas algumas viravam com certa pressa, mas não me fez impressão.

**Mod. – E o feedback que é dado durante o jogo, por exemplo os sons, algumas mensagens que aparece… como por exemplo, este produto não pertence à sua lista de compras ou consulte a sua lista de compras para saber que produtos tem de comprar. Acha esse feedback útil e necessário ou…**

**M. –** Sim, sim, sim. Acho que numa situação destas, a gente acaba por associar um determinado som… a uma ação. E esse som… faz-nos, intuitivamente que a gente entenda que está validado ou que aquilo não está validado. Nesse aspeto achei que sim, que estava bem.

**Mod. – Tem mais alguma sugestão que nos gostasse de deixar?**

**M. –** Não, acho que já disse tudo o que me parecia.

**Mod. – Ok, então vou agradecer a sua participação e o seu tempo.**

**Table S2.** Modifications Introduced in the NeuroVRehab.PT as result of the interviews.

| **Screen/game mode** | | **Modifications in the game according to type of information** |
| --- | --- | --- |
| Log-in | *Direct feedback* | |
|  | - All written content was translated to Portuguese (e.g. log-in, username, password) | |
|  | *Indirect feedback:* | |
|  | - Option to see the password instead of dots | |
| Shopping list | *Direct feedback:* | |
|  | - Main activity of the platform (see Figure 1(b)) - Shopping list organization is optional (previously it was a mandatory action) - Possibility to determine the product’s quantity (e.g. grams, kilos, liters, number of units) - Users can share/print shopping lists | |
|  | *Indirect feedback:* | |
|  | - Interface redesign and task segmentation (e.g. products selection, shopping list organization, being able to create a new shopping list or add the products to a previous one, go to the virtual supermarket) with each task presented on a different page (see Figures 4(b) and 5(b)) - Drop-down menu with all products that share the description entered by users. A name and photo of each product is available - Users can add new products to the list of products available in the virtual supermarket - The total number of points earned is shown in a pop-up window at the end of each task - Inclusion of a message at the beginning and end of all activities alerting participants that all activities performed were inconsequential (i.e. a theoretical exercise) | |

**Table S2.** Continue

| **Screen/game mode** | **Modifications in the game according to type of information** |
| --- | --- |

| Recipes | *Direct feedback:* |
| --- | --- |
|  | - Possibility to remove/delete ingredients that users cannot or usually do not consume (see Figure 4(b)) - Use of more intuitive icons (e.g. use of a bin to delete unwanted ingredients instead of a red cross) (see Figure 4(b)) - New recipes available periodically - Possibility to post recipes and cooking tips |
|  | *Indirect feedback:* |
|  | - Task segmentation into different steps, with each one presented in a different screen (e.g. choose a recipe, select the ingredients wanted, organize the shopping list) (see Figure 4(b)) - Inclusion of an icon indicating the level of difficulty of each recipe |
| Challenges  (Former supermarket game mode) | *Direct feedback:* |
|  | - The initial levels are devoted to getting familiarized with the game controls and the interface (onboarding phase) (see Figure 1(b)) |
|  | *Indirect feedback:* |
|  | - Secondary activity (former the principal activity of the platform) (see Figure 1(b)) |
| Game-elements | *Direct feedback:* |
|  | - A label explaining how many points are associated with different game actions was included on the star icon (see Figure 1(b)) - A label explaining how users can use points to access new contents was included on the crown icon (see Figure 1(b)) |

**Table S2.** Continue

| **Screen/game mode** | **Changes included according to type of information** |
| --- | --- |
| Supermarket environment | *Direct feedback:* |
|  | - Game controls (arrows) are located on the supermarket’s floor in red colour to improve identification (see Figure 3(b)) - Signboards with the section name in Portuguese were included at the beginning and the end of each supermarket aisle/section (see Figure 3(b)) - One-column shopping list (previously a two-column list), with each product’s category identified by a different colour (see Figure 2(b)) - Skip secondary activities (i.e. shopping list organization activity) and non-interactive moments (i.e. cash register icon was included that enable users to “jump” to the checkout counters regardless of their location within the supermarket) (see Figure (3)) |
|  | *Indirect feedback:* |
|  | - Map of the different supermarket sections and user’s current position (see Figure 3(b)) - Shopping strategies are available at any time by clicking on the lamp icon (see Figure 3(b)) - Users can introduce shopping strategies (see Figure 3(b)) - When paying in cash, users can select the exact amount from a board with coins and bills (not included in the previous version of the platform) |

1. This is a free translation of the original script used for the semi-structured interviews. [↑](#footnote-ref-1)
